# Pd NPs decorated on crosslinked sodium alginate modified iron-based metal–organic framework Fe(BTC) as a green multifunctional catalyst for the oxidative amidation †

**DOI:** 10.1039/d4na00151f

**Published:** 2024-05-20

**Authors:** Samaneh Koosha, Ramin Ghorbani-Vaghei, Sedigheh Alavinia, Rahman Karimi-Nami, Idris Karakaya

**Affiliations:** a Department of Organic Chemistry, Faculty of Chemistry and Petroleum Sciences, Bu-Ali Sina University 6517838683 Hamadan Iran rgvaghei@yahoo.com ghorbani@basu.ac.ir; b Department of Chemistry, Faculty of Science, University of Maragheh Maragheh Iran; c Department of Chemistry, College of Basic Sciences, Gebze Technical University 41400 Gebze Turkey

## Abstract

The primary objective of this investigation was to develop a new nanocatalyst that could produce amides by oxidative amidation of benzyl alcohol, thereby reducing its environmental harm. To achieve this, Pd nanoparticle-immobilized crosslinked sodium alginate-modified iron-based metal–organic framework Fe(BTC) (Fe(BTC)@SA/ED/Pd), with excellent activity and selectivity in modified oxidative amidation of benzyl alcohol with amines, has been described. Crosslinked sodium alginate was modified on iron-based metal–organic framework Fe(BTC). It is worth noting that Pd nanoparticles were immobilized for the first time on a novel nanocomposite based on the Fe(BTC) MOF and crosslinked sodium alginate for tandem oxidative amidation to improve the eco-friendliness and economic efficiency of the process. The synergic effects of Fe(BTC), sodium alginate, and Pd NPs are important factors influencing the catalytic activity. Easy and green synthesis methods, availability of materials, high Pd loading, available catalytic sites, high surface area, high selectivity, and simple separation from the reaction medium are effective properties in catalytic activity.

## Abstract 2

The primary objective of this investigation was to develop a new nanocatalyst that could produce amides by oxidative amidation of benzyl alcohol, thereby reducing its environmental harm.

## Graphical abstract

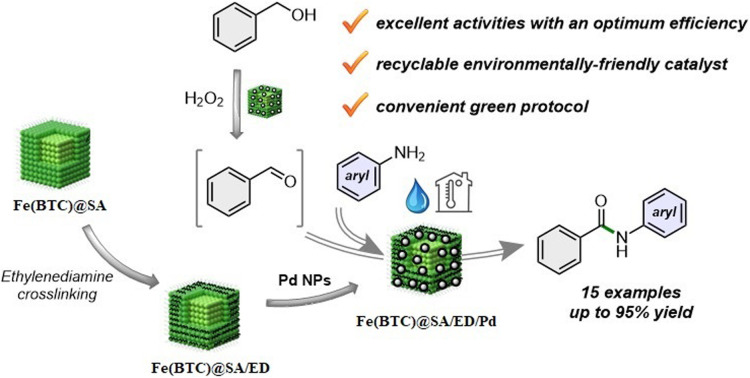

## 1. Introduction

Amide functionalities are commonly found in a range of organic compounds, polymers, natural substances, and pharmaceuticals.^1,2^ Traditional methods for synthesizing amides have several drawbacks, including the use of toxic reagents, precious metal catalysts, long reaction times, and acyl halide sources.^3,4^ Therefore, the development of atom-efficient methods is essential for achieving more sustainable and environmentally friendly chemical processes. One promising alternative for amide synthesis is the use of alcohols. Alcohols can be converted to amides through a tandem oxidation process under oxidative conditions, either *in situ via* carboxylic acids or aldehydes.^4,5^ These oxidative amidation reactions from alcohols provide attractive pathways to reduce the consumption of organic compounds, synthetic costs, and the need for complex synthetic methods.^6^ While alternative methods have been explored, oxidative amidations have gained significant attention due to their simplicity, cost-effectiveness, and reliance on easily accessible starting materials.^7^ To enable efficient amide syntheses, various homogeneous and heterogeneous catalysts have been developed, and researchers are continuously working towards creating recyclable, efficient, and environmentally friendly catalysts to meet the requirements of green chemistry.^5,8–11^

In the current scientific and technological landscape, there is a strong emphasis on the use of sustainable processes and materials.^12–16^ Metal–organic frameworks (MOFs) are nanoscale materials that have shown hope-giving results in various catalytic applications due to their strong Brønsted acidity, high thermal stability, high surface area, and numerous active sites. MOFs are composed of metal ions or clusters and custom-designed organic ligands, offering a vast array of structural motifs and SBUs that can be incorporated into materials.^17–19^ These features make MOFs promising candidates in the field of materials science and related applications.^20,21^ These materials can have tunable pore dimensions, high surface-to-volume ratios, dispersed catalytic sites in the matrix, tunable metal concentration, and a well-defined crystal medium.^22^ Notable features of MOFs include their ability to select and design suitable ligands with various types of metal knots with suitable and predictable geometry, robustness, and different coordination modes.^23,24^ Furthermore, their consistent pore structure, expansive surface area (around 4000 m^2^ g^−1^), and adjustable composition render them highly suitable for various applications such as drug delivery, gas detection, separation processes, and heterogeneous nanocatalysis.^25–28^

Fe-based MOFs exhibit high porosity and surface area, which make them promising materials for applications such as gas storage and separation, catalysis, sensing, proton conduction, and drug delivery.^29^ Fe-based MOFs have been widely and successfully applied as catalysts for reactions requiring acidic or redox centers.^30,31^ Surface modification of MOFs enhances their compatibility and reactivity, leading to improved interfacial interactions with the polymer matrix and enhanced performance in various applications.^17^

Sodium alginate, a naturally occurring biopolymer and linear heteropolysaccharide, finds broad applications in the food industry as a thickening and stabilizing agent.^32^ Besides, it is also utilized in drug delivery, catalysis, and photocatalysis.^33,34^ The immobilization of sodium alginate on a heterogeneous support has been proposed as a potentially favorable method to improve the catalytic efficacy of these materials.^35–38^ This approach offers the added benefits of mitigating cost and environmental concerns, making it an attractive avenue for further research and development. Despite the potential advantages MOFs offer, their restricted chemical degradation, unwanted oxidation, and aggregation limitations have been identified as significant challenges.^39–42^ The immobilization of sodium alginate into MOFs offers the potential for sustainable and environmentally friendly catalytic processes.

**Scheme 1:**
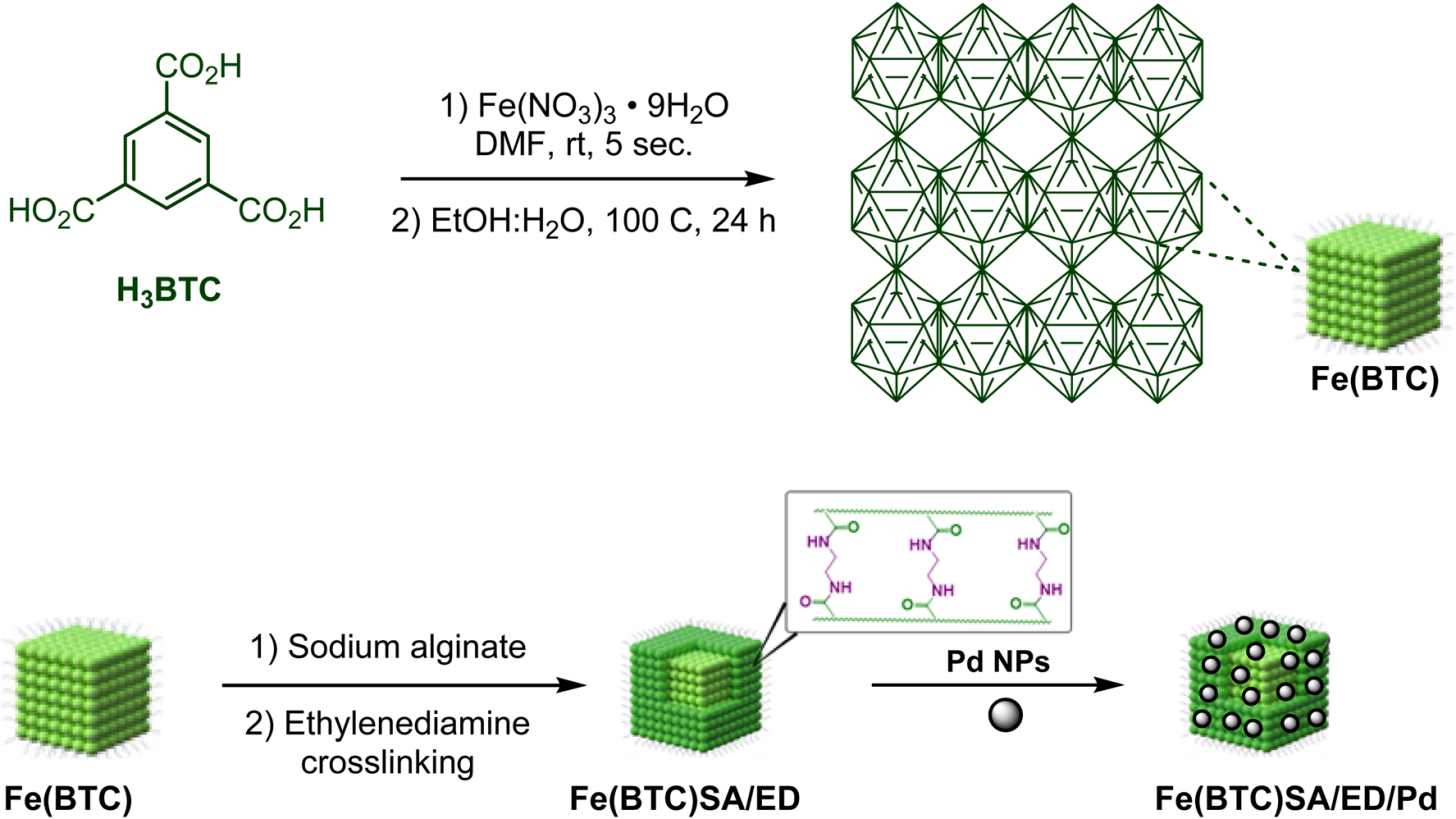
The synthesis of the Fe(BTC)@SA/ED/Pd nanocomposite.

**Scheme 2:**
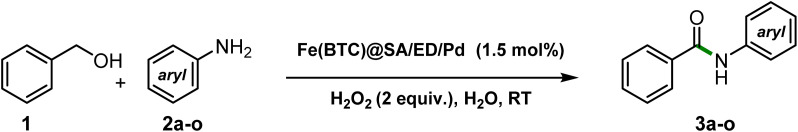
General procedure for the one-pot synthesis of secondary amides using the Fe(BTC)@SA/ED/Pd nanocomposite.

In this study, we introduce a novel bio-derived nanocatalyst composed of crosslinked sodium alginate modified on the surface area of Fe(BTC). To enhance the chemical and thermal stability of sodium alginate (SA), ethylene diamine was used as a chemical crosslinker. Fe(BTC) and crosslinked sodium alginate are essential in Pd loading (Scheme 1). We then focused on synthesizing secondary amides through a tandem green oxidative amidation using the Fe(BTC)@SA/ED/Pd nanocomposite. The reaction demonstrated a broad substrate scope for a variety of aniline derivatives with electron withdrawing/donating substituents with yields between 83 and 95% at room temperature (Scheme 2). Finally, the catalyst's high stability was demonstrated by its successful recovery after several cycles without reducing efficiency. The present methodology is the first report of a composite based on Fe(BTC) and crosslinked sodium alginate for the selective synthesis of amides using the oxidative amidation reaction.

## 2. Preparation of the Fe(BTC)@SA/ED/Pd nanocomposite

### 2.1. Preparation of Fe(BTC)@SA/ED

The synthesis of Fe(BTC) was achieved through the reaction of trimesic acid (H_3_BTC) (8.3 mmol) and iron nitrate hexahydrate (8.7 mmol) in DMF (30 mL). Subsequently, the mixture of H_2_O : EtOH (30 mL, 1 : 1) was added and sonicated for 35 more minutes. Finally, the mixture was heated in a sand bath for 24 h at 100 °C.^43^ Then, sodium alginate (0.5 g) and Fe(BTC) (0.5 g) were stirred with a magnetic stirrer in the presence of DCC (*N*,*N*′-dicyclohexylcarbodiimide) (0.2 g) in DMF (30 mL) solution at 60 °C for 1 hour. Subsequently, ethylene diamine (5 mL) was added to the reaction mixture at 100 °C and stirred for 24 hours. The obtained solid product was washed with ethanol and dried at 50 °C (Scheme 1).

### 2.2. Preparation of the Fe(BTC)@SA/ED/Pd nanocomposite

0.315 g of the prepared Fe(BTC)@SA nanocomposite support was dispersed in ethanol (40 mL) solvent for 30 minutes, and then PdCl_2_ (0.157 g) was added to the reaction mixture under a N_2_ atmosphere and stirred for 24 hours. Next, to reduce PdCl_2_, NaBH_4_ (0.141 g) was slowly added to the mixture and, placed under a N_2_ atmosphere and stirred for 2 hours. Finally, the Fe(BTC)@SA/ED/Pd nanocomposite was washed with DI water and ethanol and dried under vacuum at 40 °C (Scheme 1). The results from ICP-OES analysis revealed that the percentage of Pd is 17.89%.

### 2.3. General procedure for the one-pot synthesis of secondary amides

In a standard experimental procedure, the following components were combined in a 25 mL round-bottomed flask: 1.0 mmol of benzyl alcohol, 2.0 mmol of aqueous H_2_O_2_ (30%), and 1.0 mol% of basolite@SA/ED/Pd NP catalyst in 3 mL of H_2_O. The mixture was then stirred at room temperature for 2 hours. After this initial reaction period, 1.0 mmol of aniline was introduced into the reaction mixture. Upon completion of the reaction, the catalyst was separated by using centrifugation, and the organic residue was subsequently extracted with ethyl acetate. The solvent was then removed under reduced pressure. Finally, the resulting product was subjected to purification through column chromatography (Scheme 2).

## 3. Results and discussion

### 3.1. Fe(BTC)@SA/ED/Pd nanocomposite characterization

**Fig. 1:**
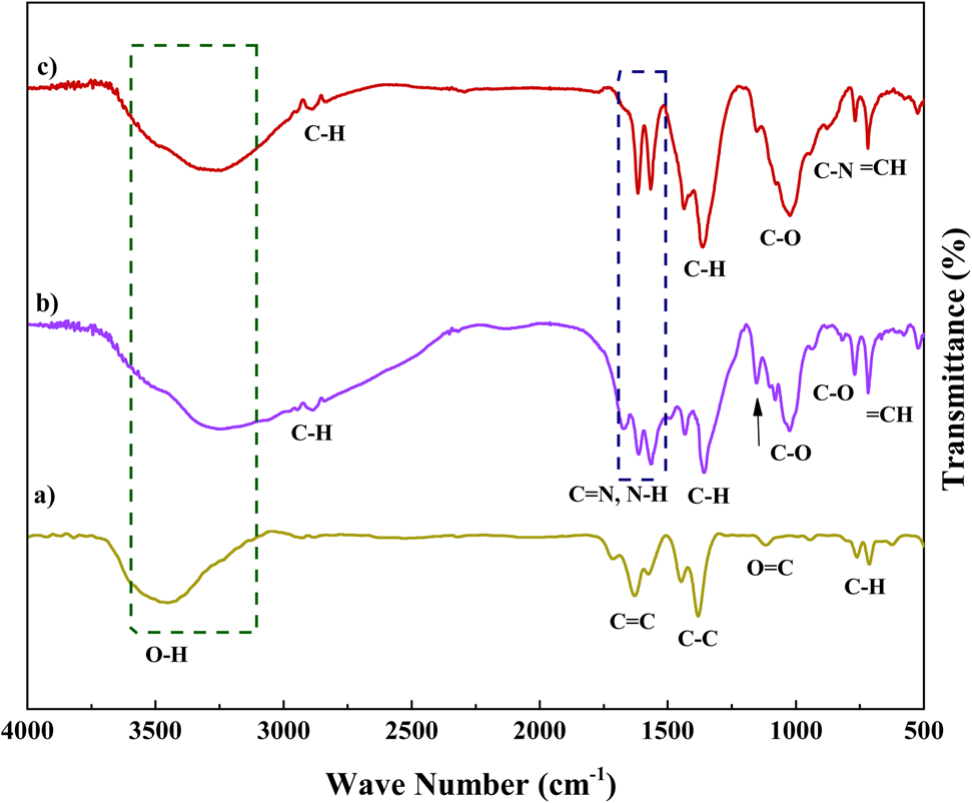
FTIR spectra of Fe(BTC) (a), Fe(BTC)@SA/ED (b), and Fe(BTC)@SA/ED/Pd (c).

Fig. 1 displays the FT-IR absorption spectra of Fe(BTC), Fe(BTC)@SA/ED, Fe(BTC)@SA/ED/Pd, and the recycled Fe(BTC)@SA/ED/Pd nanocomposite. The symmetric modes of the O–H bonds attached to the Fe atoms are indicated by the stretching vibrations at 3400 cm^−1^ (Fig. 1a–c). The successful functionalization is confirmed by comparing the spectra of Fe(BTC) and the Fe(BTC)@SA/ED nanocomposite (Fig. 1b with Fig. 1a). The presence of a new peak at 1630 cm^−1^ strongly suggests amide vibration. Furthermore, the interaction between Fe(BTC)@SA/ED and Pd NPs is evident in the FT-IR spectra of the Fe(BTC)@SA/ED/Pd nanocomposite catalyst, as the C=O vibration of amide shifts from 1630 cm^−1^ to 1615 cm^−1^ (Fig. 1c).

**Fig. 2:**
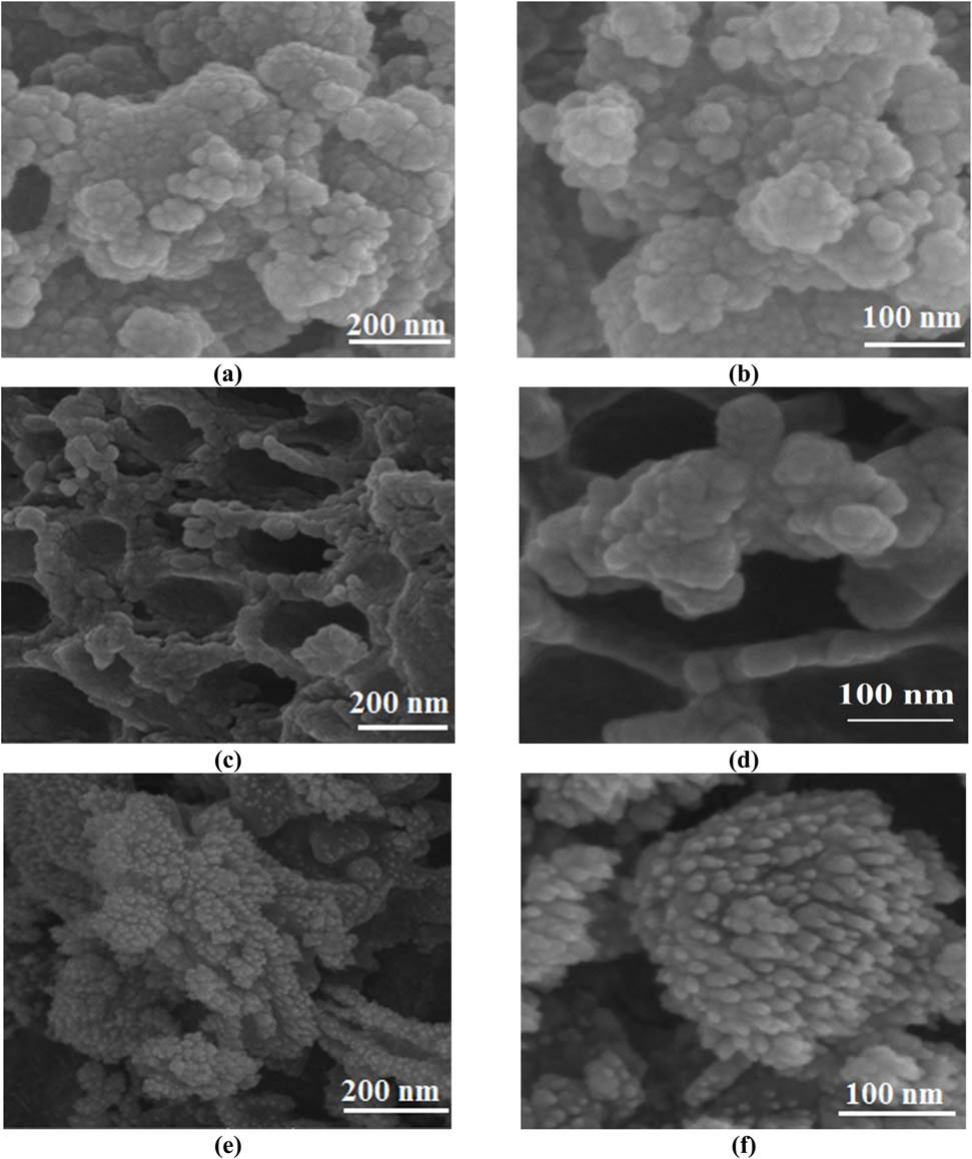
FESEM images of Fe(BTC) (a and b), Fe(BTC)@SA/ED (c and d), and Fe(BTC)@SA/ED/Pd (e and f).

FESEM images present good information about the morphology and particle size of Fe(BTC), Fe(BTC)@SA, and the Fe(BTC)@SA/ED/Pd nanocomposite (Fig. 2). The FESEM image of Fe(BTC) indicates almost a spherical structure morphology (Fig. 2a and b). In addition, according to Fig. 2c and d, there is no significant change even after the immobilization of crosslinked sodium alginate. It is worth noting that Fe(BTC)@SA/ED successfully retained Pd NP species within the pores, preventing their aggregation (Fig. 2e and f).

**Fig. 3:**
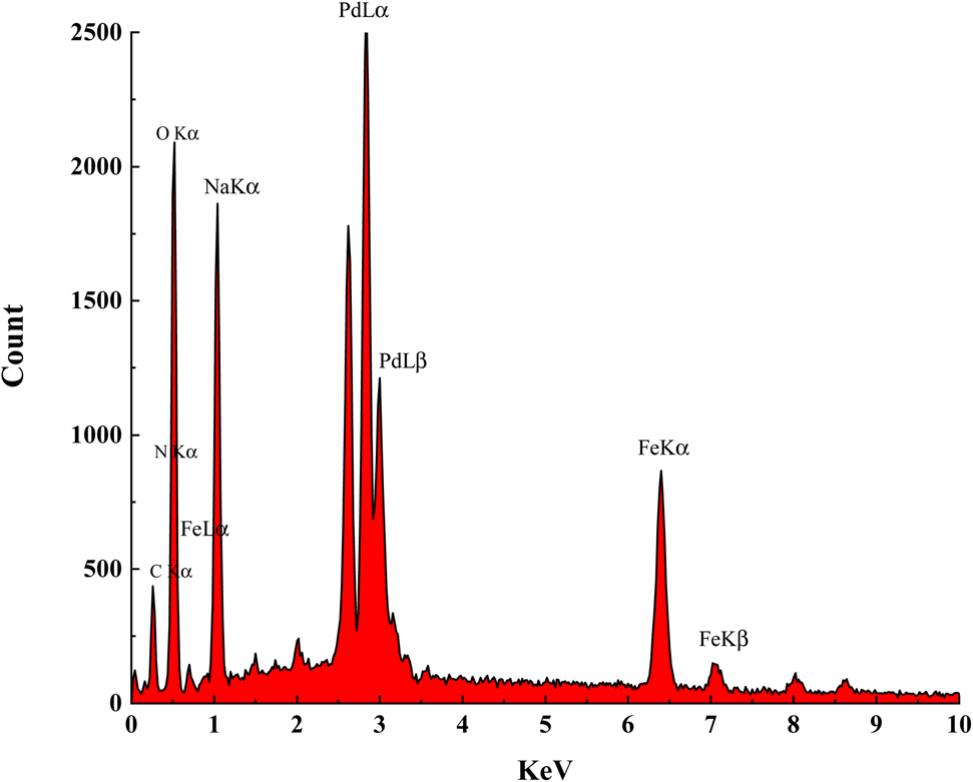
EDS analysis of Fe(BTC)@SA/ED/Pd.

The elemental composition of Fe(BTC)@SA/ED/Pd was studied using EDS analysis in a selected zone (Fig. 3). Through elemental mapping, it was confirmed that all elements, including Fe, O, C, Pd, N, and Na, were present and evenly distributed. This analysis provided validation for the successful fabrication of the Fe(BTC)@SA/ED/Pd nanocomposite. The results indicated that the catalyst contained 0.96 N, 6.65 C, 4.35 Na, 13.72 O, 36.46 Fe, and 37.86 Pd.

**Fig. 4:**
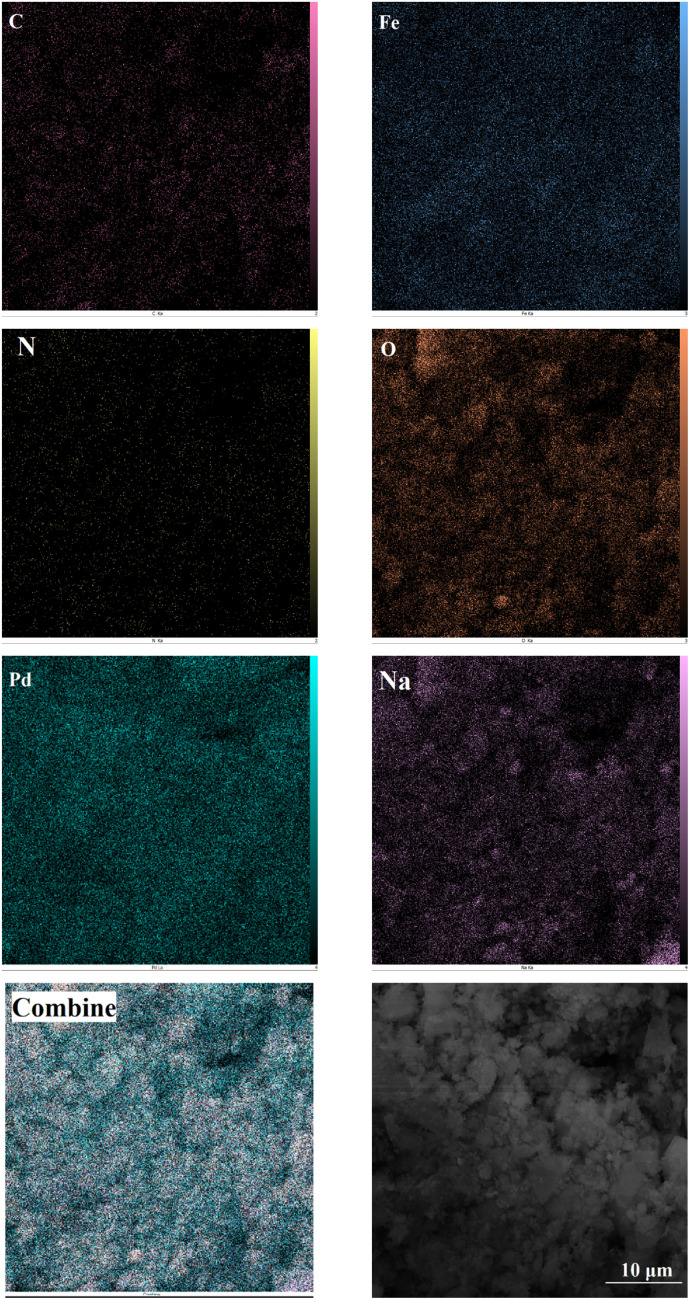
Elemental mapping of the C, N, O, S, Pd, Na, and Fe atoms in the Fe(BTC)@SA/ED/Pd nanocomposite.

The elemental mapping analysis of Fe(BTC)@SA/ED/Pd shows that the distribution patterns of carbon, nitrogen, oxygen, iron, palladium, and sodium elements are consistent, indicating that the amide groups of the crosslinked sodium alginate ligand act as donor nitrogen atoms for the coordination of Pd NPs (Fig. 4). An increase in the number of active sites is expected to result in a higher production rate of organic products due to the uniform dispersion of copper as the active component in this heterogeneous catalyst.

**Fig. 5:**
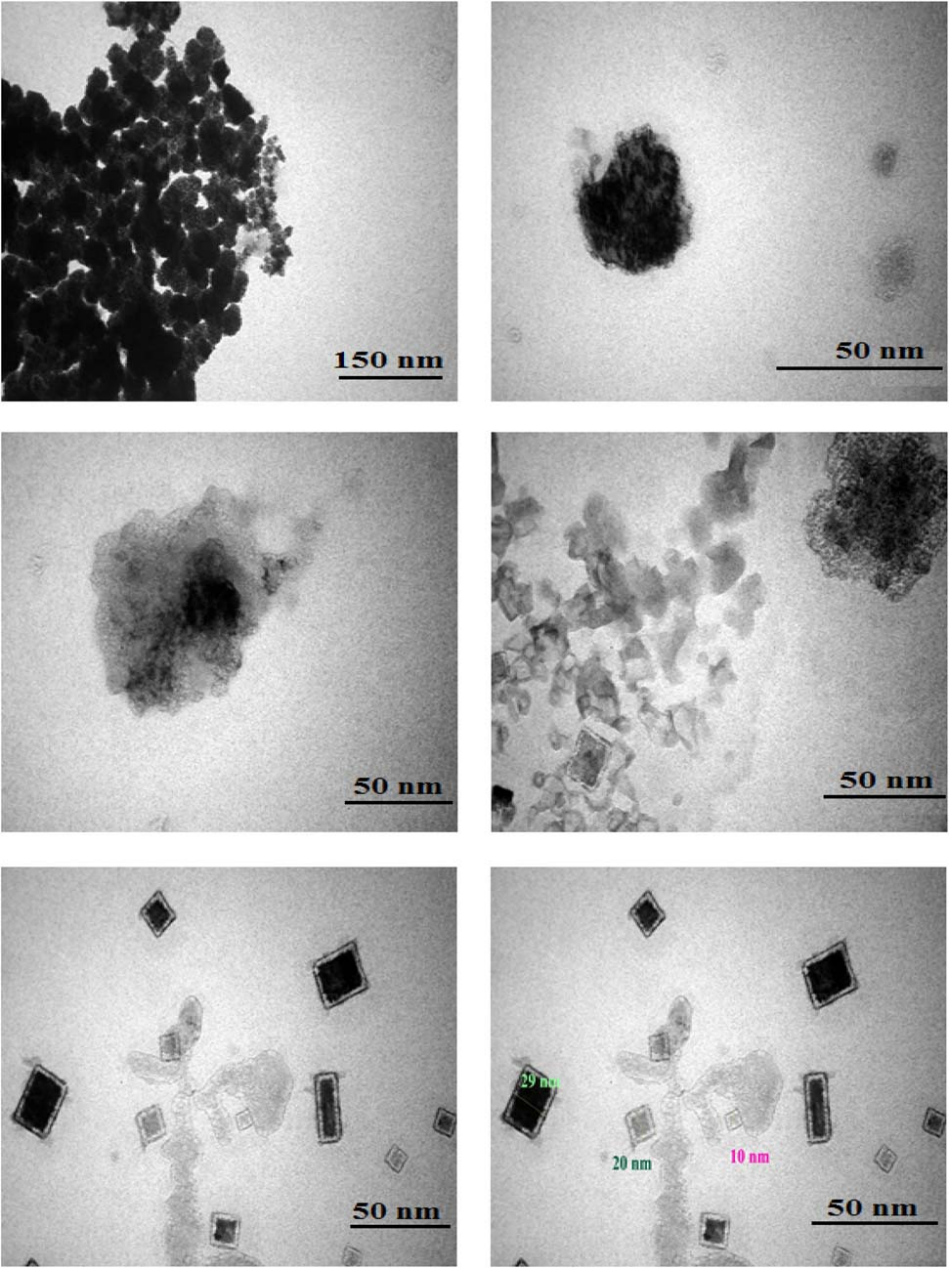
TEM images of Fe(BTC)@SA/ED/Pd at different scale bares.

The configuration and distribution of particles were examined through TEM analysis. According to the TEM images of Fe(BTC)@SA/ED/Pd, various sizes and morphologies of SA, Pd NPs, and Fe(BTC) particles can be observed. Fig. 5 depicts the amalgamation and merging of quasi-spherical entities of sodium alginate. Notably, when Pd NPs are immobilized on Fe(BTC)@SA/ED, it leads to the synthesis of spherical Pd NPs. The Fe-BTC MOF is characterized by quasi-spheric amorphous nanoparticles with average sizes of 10 to 50 nm. At higher magnification, the nanopore network is clearly visible and shows a darker contrast than the surrounding material. The morphological characteristics of sodium alginate, Fe(BTC), and Pd NP samples are clearly consistent with the findings derived from the analysis conducted in previous sections.

**Fig. 6:**
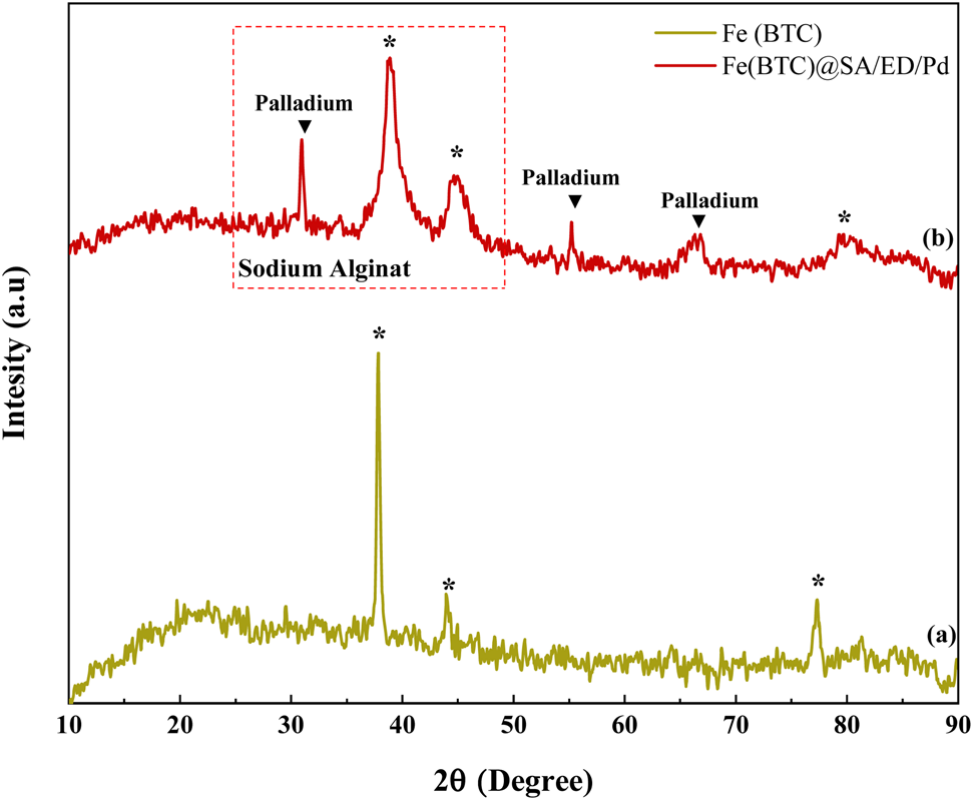
XRD patterns of Fe(BTC) (a) and Fe(BTC)@SA/ED/Pd samples (b).

The crystal structure of Fe(BTC) and Fe(BTC)@SA/ED/Pd samples was analyzed using XRD analysis (Fig. 6). The diffraction peaks of Fe(BTC) were observed at 2*θ* angles of 37.87°, 43.92°, and 77.30°, which match the pattern reported in the literature (Fig. 6a). In the XRD spectrum of Fe(BTC)@SA/ED/Pd, slight shifts in position and width were observed, along with a few intense peaks in the 2*θ* angles of 25°–45°, indicating the presence of sodium alginate in the nanocatalyst.^44^ Additionally, sharp peaks at 2*θ* angles of 30.96°, 55.17°, and 66.30°confirm the successful synthesis of Pd NPs on the surface of Fe(BTC)@SA/ED^45,46^ (Fig. 6b). The peak shift in the XRD pattern of the composite is related to the structural changes of the synthesized product, and it could be an effect of preferential orientation on the basal spacing^47–49^

**Fig. 7:**
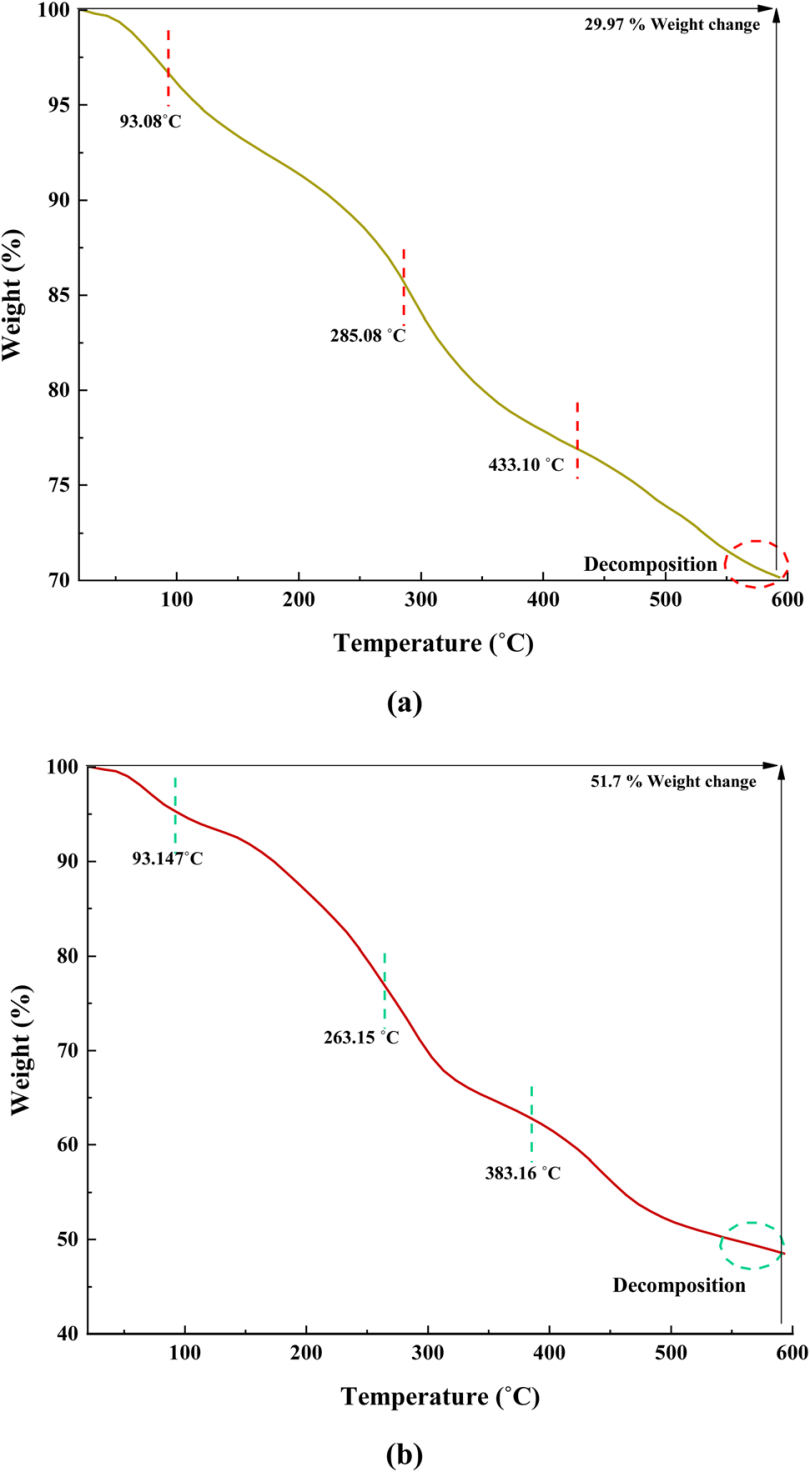
TGA curves of Fe(BTC) (a) and Fe(BTC)@SA/ED/Pd (b).

Fig. 7 illustrates the TGA curves of Fe(BTC) and the Fe(BTC)@SA/ED/Pd nanocomposite, depicting the residual masses within the temperature range of 25 to 600 °C. The initial portion of the TGA curve shows a negligible weight loss between 90 and 180 °C, confirming the evaporation of absorbed solvent present on the surface of Fe(BTC) and the Fe(BTC)@SA/ED/Pd nanocomposite. The weight loss continues until 600 °C, signifying the decomposition of sodium alginate and the carbon chain backbone of Fe(BTC). The TGA curve of Fe(BTC) exhibits a three-step degradation pattern, occurring at 93.08 °C, 285.08 °C, and 433.10 °C, resulting in a weight loss of 29.92% (Fig. 7a). Similarly, the Fe(BTC)@SA/ED/Pd nanocomposite undergoes three stages of decomposition at 93.14 °C, 263.15 °C, and 383.16 °C, leading to a weight loss of 51.7% (Fig. 7b).

**Fig. 8:**
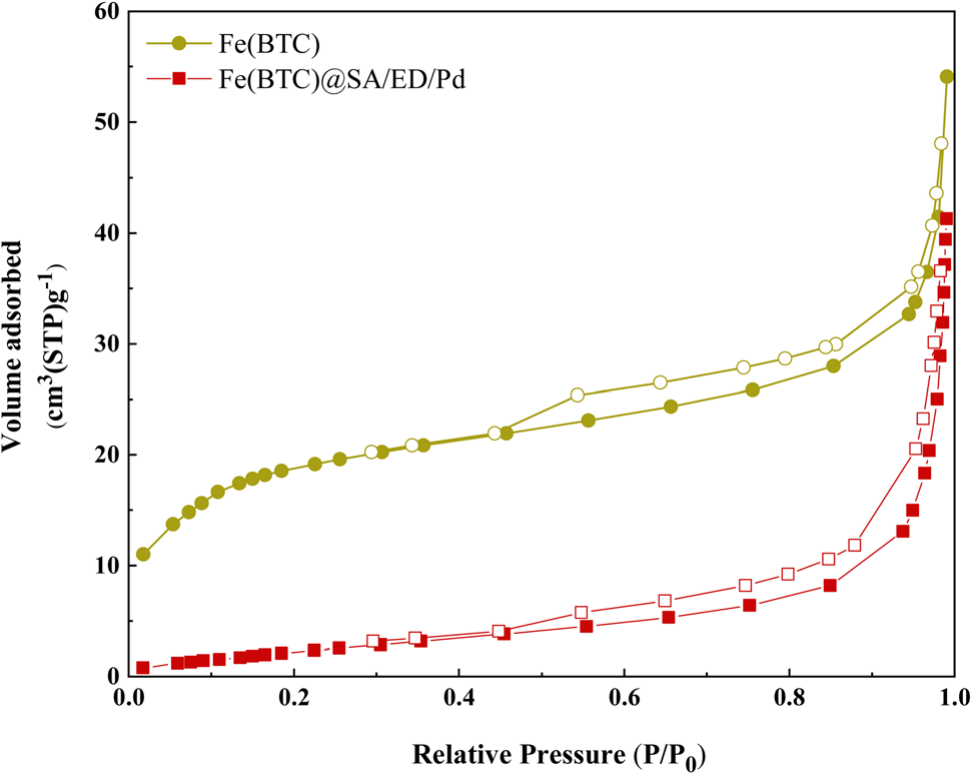
N_2_ adsorption isotherms by the BET analysis of Fe(BTC) (a) and Fe(BTC)@SA/ED/Pd (b).

To confirm the porous nature and estimate the surface area of the catalyst, nitrogen adsorption–desorption isotherm measurements were conducted on Fe(BTC) (Fig. 8a) and Fe(BTC)@SA/ED/Pd (Fig. 8b). These isotherms displayed typical type V isotherms associated with mesopores, along with type H3 hysteresis loops. The specific surface areas of Fe(BTC) and Fe(BTC)@SA/ED/Pd were determined using the Langmuir adsorption isotherm as 77.65 and 10.2 m^2^ g^−1^, respectively. Table 1 shows the comparison between Fe(BTC)@SA/ED/Pd and Fe(BTC) MOF. The decrease in BET surface area can be explained by the physical and chemical interactions between Fe(BTC) with crosslinked sodium alginate and Pd NPs.^50^

**Table 1:** Results of the Langmuir and BET measurements of Fe(BTC) and Fe(BTC)@SA/ED/Pd

Parameter	Fe(BTC)	Fe(BTC)@SA/ED/Pd
*a*_s_ (m^2^ g^−1^)	77.65	10.2
*V*_m_ (cm^3^(STP) g^−1^)	15.47	1.52
*V*_p_ (cm^3^ g^−1^)	0.064	0.060
*r*_p_ (nm)	2.20	1.21
*a*_p_ (m^2^ g^−1^)	25.33	13.47

### 3.2. Model reaction optimization

Different parameters were examined to identify the most suitable conditions for the reaction. Various factors were taken into account, such as the quantity of catalyst, the type of oxidizing agents, the choice of solvent, the duration of the reaction, and the temperature conditions. The investigation was conducted using *p*-methylaniline and benzyl alcohol as model substrates (Table 2). The oxidation of benzyl alcohol did not occur when H_2_O_2_ was used without the addition of the Fe(BTC)@SA/ED/Pd catalyst (entry 1). Therefore, the use of the Fe(BTC)@SA/ED/Pd catalyst is essential for this conversion. Following that, the experiment was conducted using different quantities of catalyst to establish the most effective catalyst proportion (Table 2, entries 2–4). The best result was obtained when using 10 mg of Fe(BTC)@SA/ED/Pd catalyst (entry 2). Increasing the catalyst amount beyond 10 mg did not enhance the reaction efficiency (entry 3). In the absence of H_2_O_2_, it was noted that the reaction did not proceed (entry 5). Results from experiments using various oxidants like SeO_2_, NaClO, and MnO_2_ indicated that H_2_O_2_ remained the superior oxidant (entries 6–8). Furthermore, we conducted experiments exploring various solvents for the oxidative amidation of benzyl alcohol and aniline (entries 9–13). Water as a solvent yielded better product yields due to the amphiphilic properties of the Fe(BTC)@SA/ED/Pd nanocomposite. Ethanol exhibited lower solubility for the synthesized catalyst, resulting in a reduced yield. Notably, the presence of DMF, chloroform, PEG, and toluene solvents decreased the reaction efficiency. Finally, the efficiency of the Fe(BTC)@SA/ED/Pd catalyst was compared with the catalyst precursors Fe(BTC)@SA/ED, Fe(BTC)/Pd, and Fe(BTC) and, the best result was obtained in the presence of Fe(BTC)@SA/ED/Pd (Table 2, entries 14–16). In the presence of Fe(BTC) and Fe(BTC)@SA/ED, no amide product was obtained, and just oxidation of alcohol to aldehyde occurred. Increasing the reaction time had no effect on increasing the efficiency (Table 2, entry 17).

**Table 2:** Model reaction optimization^a^

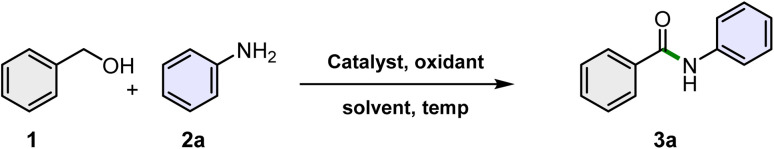						
Entry	Cat. (mg)	Oxidant	Solvent	Yield (%)^b^	TOF	TON
1	—	H_2_O_2_	H_2_O	N.R.	—	—
2	Fe(BTC)@SA/Pd (10)	H_2_O_2_	H_2_O	95	47.5	95
3	Fe(BTC)@SA/Pd (15)	H_2_O_2_	H_2_O	95	31.6	63.33
4	Fe(BTC)@SA/Pd (5)	H_2_O_2_	H_2_O	75	75	150
5	Fe(BTC)@SA/Pd (10)	—	H_2_O	Trace	—	—
6	Fe(BTC)@SA/Pd (10)	MnO_2_	H_2_O	80	40	80
7	Fe(BTC)@SA/Pd (10)	SeO_2_	H_2_O	65	32.5	65
8	Fe(BTC)@SA/Pd (10)	NaClO	H_2_O	50	25	50
9	Fe(BTC)@SA/Pd (10)	H_2_O_2_	EtOH	52	26	52
10	Fe(BTC)@SA/Pd (10)	H_2_O_2_	DMF	39	19.5	39
11	Fe(BTC)@SA/Pd (10)	H_2_O_2_	CHCl_3_	37	18.5	37
12	Fe(BTC)@SA/Pd (10)	H_2_O_2_	PEG	58	29	58
13	Fe(BTC)@SA/Pd (10)	H_2_O_2_	Toluene	39	19.5	39
14	Fe(BTC) (10)	H_2_O_2_	H_2_O	25	12.5	25
15	Fe(BTC)@SA (10)	H_2_O_2_	H_2_O	30	15	30
16	Fe(BTC)@ Pd (10)	H_2_O_2_	H_2_O	52	26	52
17	Fe(BTC)@SA/Pd (10)	H_2_O_2_	H_2_O	95^c^	31.6	95

a Reaction conditions: benzaldehyde (1 mmol), aniline (1.5 mmol), oxidant (2–4 mmol), and Fe(BTC)@SA/ED/Pd nanocomposite (1.0 mol%) in 3 mL solvent at room temperature for 2 h.

b Isolated yield.

c The reaction was investigated at 3 h.

**Scheme 3:**
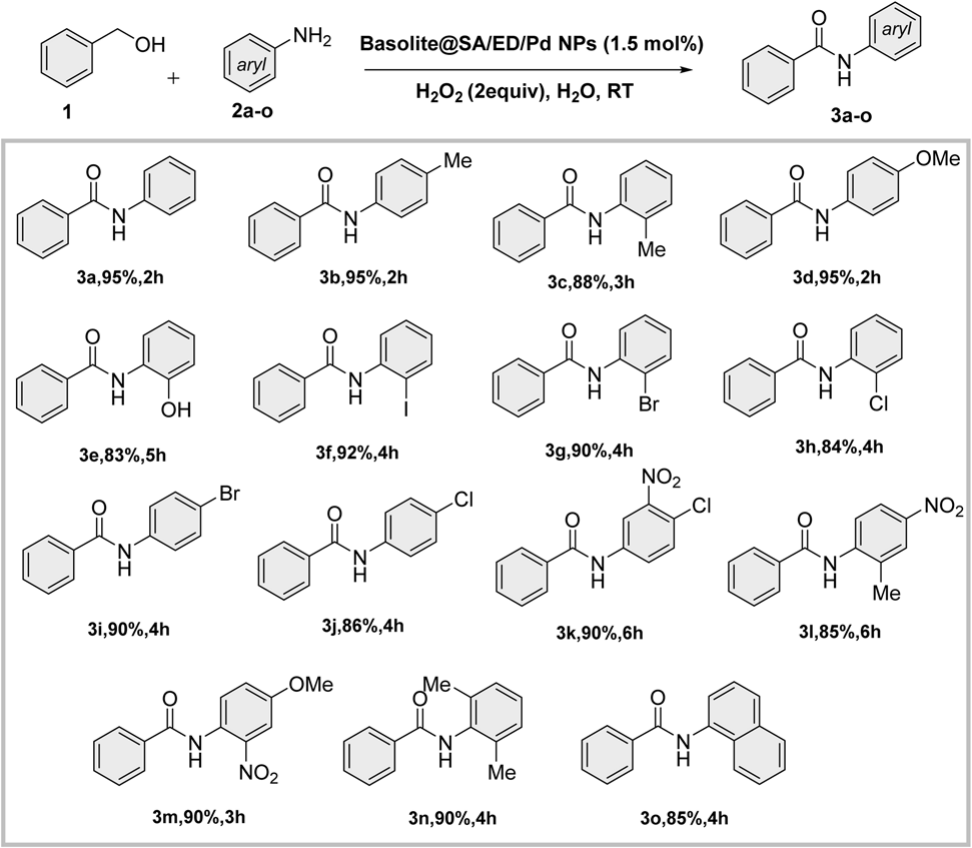
Tandem oxidative amidation of benzylic alcohol with various anilines.

The reaction between primary aromatic alcohols and various amines was further investigated after achieving optimal conditions. The goal was to understand the general mechanism and scope of the reaction. This investigation revealed that amide formation occurs through the sequential oxidation of alcohol to aldehyde, followed by amine-mediated amide formation in a one-pot process. The reactivity of different amine derivatives with various substituents (Cl, Br, NO_2_, OCH_3_, CH_3_, I, OH) was found to be moderate to good for synthesizing the desired amides. Aromatic amines with electron-donating groups on the aromatic ring showed higher yields. Additionally, satisfactory yields of the desired products (3c, 3e, 3f, 3g, 3h, 3k, 3l, 3m, and 3n) were obtained when substrates with *meta*-substituted and *ortho*-substituted aryl ring anilines were present (Scheme 3).

**Scheme 4:**
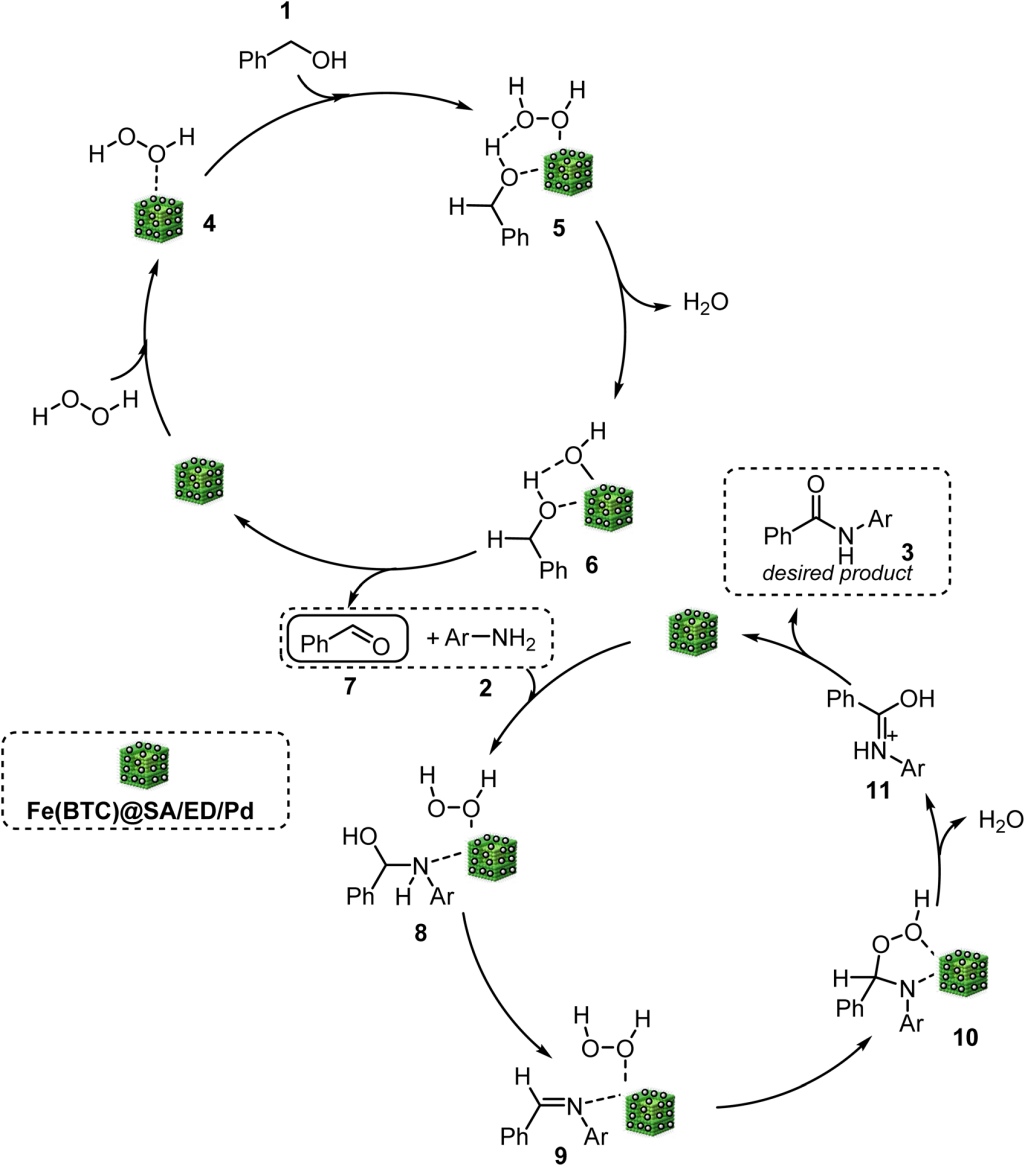
General mechanism of tandem oxidative amidation.

According to the literature, the first step was oxidation of benzyl alcohol through the interaction between Pd NP active sites and H_2_O_2_, resulting in the creation of a peroxo complex (5).^5^ This complex then reacts with benzyl alcohol, forming an intermediate (6), leading to the production of benzaldehyde through dehydration (7). Furthermore, the oxidation of benzyl alcohol to benzaldehyde is facilitated by the dual-functional catalyst Fe(BTC) MOF. The formation of secondary amide occurs when an aldehyde reacts with aniline in the presence of Pd NPs on the surface of the synthesized nanocatalyst. During the oxidation and/or rearrangement process, hydroperoxide intermediates are formed from hemiaminal and/or imine intermediates. Finally, the oxidation of intermediate (8) in the presence of Pd NPs and Fe(BTC) leads to obtaining the final product (Scheme 4).

**Fig. 9:**
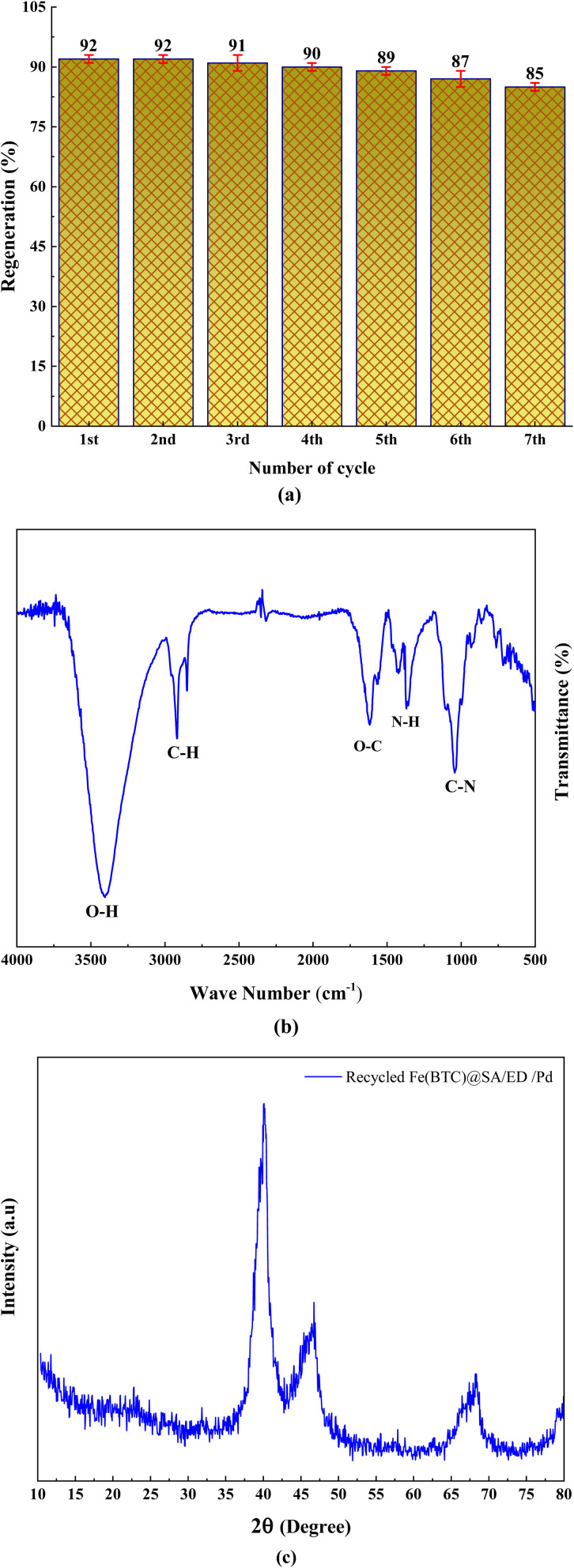
Recyclability of the Fe(BTC)@SA/ED/Pd nanocatalyst for the reaction of aniline and benzyl alcohol (a), FTIR spectrum of recycled Fe(BTC)@SA/ED/Pd (b), and XRD pattern of the recycled Fe(BTC)@SA/ED/Pd nanocomposite (c).

In order to further assess the performance of the Fe(BTC)@SA/ED/Pd nanocomposite, we conducted a study on its stability and ability to be reused in the amidation reaction using a model reaction. Once the reaction was finished, we separated the insoluble catalyst from the product through centrifugation. The catalyst was then washed and dried at 70 °C for 6 hours before being utilized again in the next recycling process. Impressively, we discovered that the Fe(BTC)@SA/ED/Pd nanocatalyst displayed both remarkable stability and reusability, as it maintained its effectiveness without any noticeable decline for up to 6 consecutive cycles (Fig. 9). The FT-IR analysis of the recycled catalyst demonstrates its excellent chemical stability (Fig. 9b). The XRD spectrum of the catalyst after seven consecutive runs is depicted in Fig. 9c. As can be seen, there was no significant change in the XRD spectrum of the reused catalyst, indicating that the catalytic reaction did not alter the chemical structure of the catalyst.

**Fig. 10:**
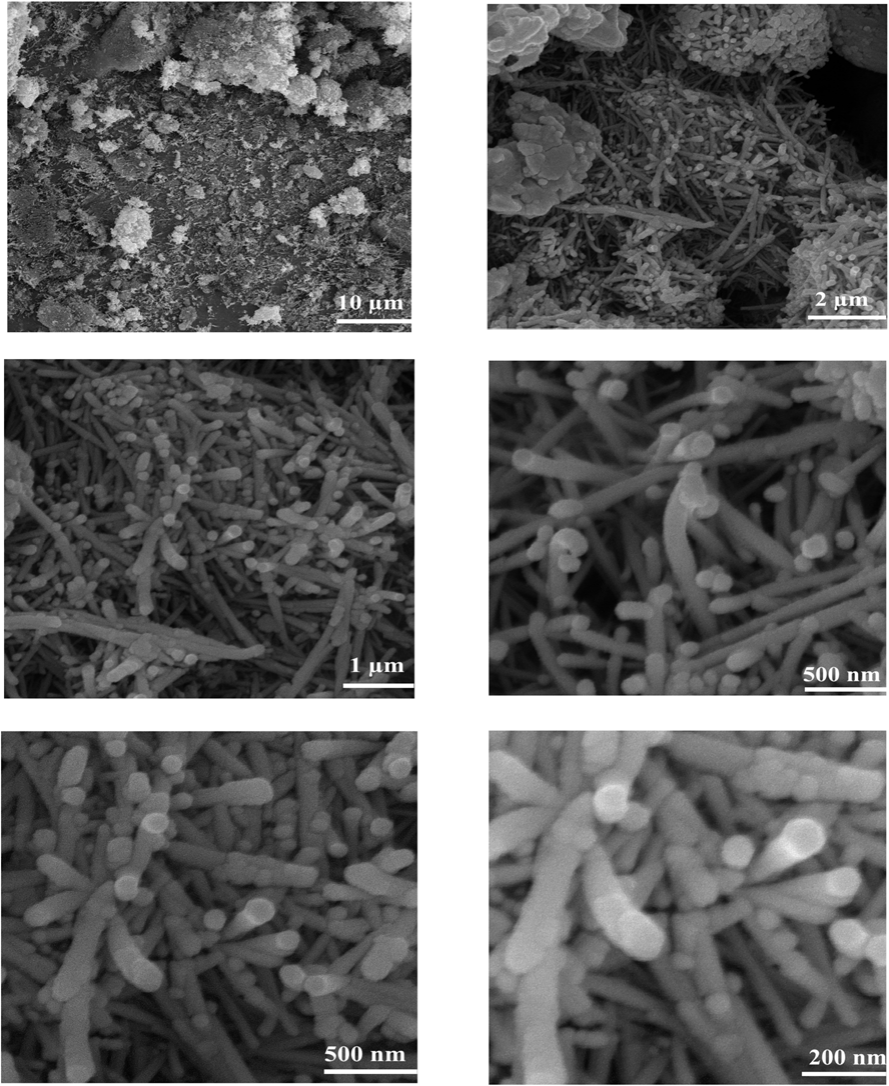
FESEM images of the recycled Fe(BTC)@SA/ED/Pd nanocomposite at different scale bars.

FESEM analysis, illustrated in Fig. 10, validated the integrity of palladium species, affirming their non-aggregation during the catalytic cycles The FESEM image obtained after seven consecutive reactions with the catalyst illustrated no significant changes in the configuration and distribution of particles. This observation underscores the stability and sustained particle retention within the composite substrate after seven consecutive catalytic reactions.

**Fig. 11:**
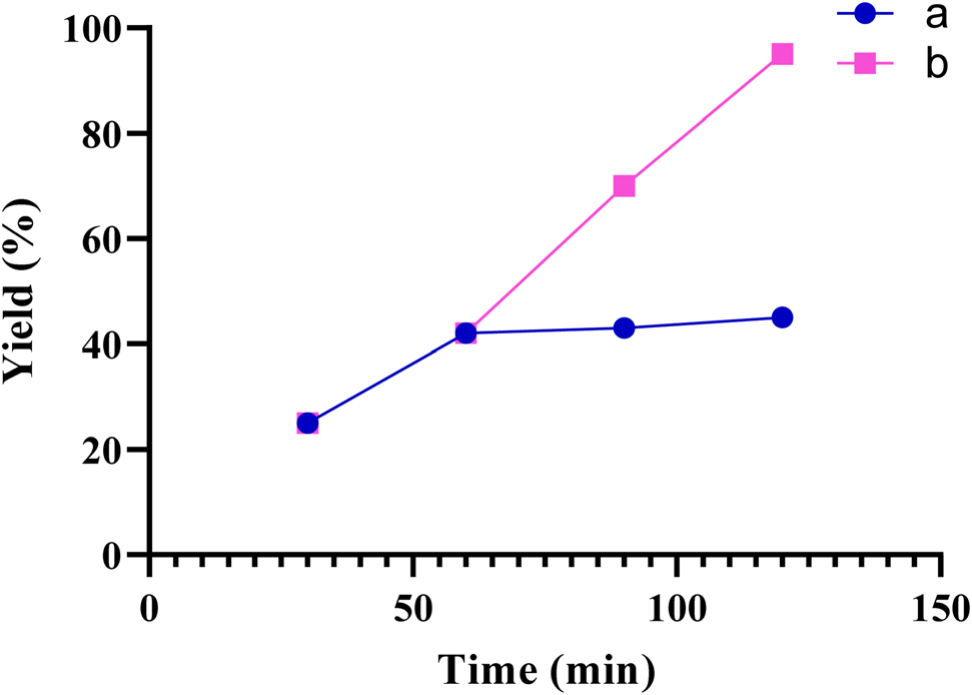
Hot-filtration test for the synthesis of product 3a. (a, the catalyst was filtered at 60 min, b, normal reaction).

To determine the heterogeneous nature of the catalyst in the model reaction (Table 2, entry 3), the hot filtration method was employed. When the catalyst reached 50% conversion (*t* = 60 min), it was separated from the reaction vessel. The resulting filtrate was then reintroduced into the vessel and allowed to react for an additional 60 min. However, no notable advancement was observed. Consequently, the hot filtration test confirmed that the Fe(BTC)@SA/ED/Pd nanocomposite nanoparticles were genuinely heterogeneous (Fig. 11).

Next, we compared the Fe(BTC)@SA/ED/Pd nanocatalyst with other reported catalysts in order to investigate its merits (Table 3). The results clearly demonstrate that the Fe(BTC)@SA/ED/Pd nanocomposite (1.5 mol%) outperforms the rest in terms of both reaction time and yield. This study presents an environmentally friendly approach, where the reaction occurs in water at a mild temperature, ensuring quick completion. The catalyst, made up of sodium alginate, polysulfonamide, and Fe(BTC), is both biodegradable and environmentally friendly. Furthermore, the catalyst synthesized in this study exhibits a high loading capacity, reducing the amount of catalyst needed for the reaction (entry 8).

**Table 3:** Comparison of the present work with previous studies

Entry	Catalyst	Oxidant	Solvent	*T* (^o^C)	Time (h)	Yield (%)	Ref.
1	MnO_2_	O_2_	Toluene	100	24	57	51
2	GO/PdNPs	H_2_O_2_	H_2_O	RT	15–24	94	52
3	FeCl_3_	EDC	TEMPO	90	8–32	75–92	53
4	MnO_2_/GO	O_2_	H_2_O	150	3–30	98	54
5	PS-BHA-Cu	TBHP	DMF–H_2_O	80	5	99	55
6	AuPd/resin	O_2_	H_2_O	40	12	49–99	56
7	CuSO_4_·5H_2_O	TBHP	CH_3_CN	80	24	46–93	57
8	Fe(BTC)@SA/ED/Pd	H_2_O_2_	H_2_O	RT	2–6	84–95	This Work

## 4. Conclusion

In summary, a novel environmentally-friendly Fe(BTC)@SA/ED/Pd nanohybrid containing sodium alginate, crosslinked sodium alginate, Fe(BTC), and PdNPs was synthesized to improve the catalytic activity for the selective aerobic oxidative synthesis of primary aryl amides from benzylic alcohol. Various aniline derivatives with electron-withdrawing and electron-donating groups successfully produced the desired amides with excellent selectivity under mild conditions.

## Conflicts of interest

There are no conflicts to declare.

## Supplementary Material

NA-006-D4NA00151F-s001
