# Supplementary material for: Pd NPs decorated on crosslinked sodium alginate modified iron-based metal–organic framework Fe(BTC) as a green multifunctional catalyst for the oxidative amidation
Source: Nanoscale Adv. 2024 May 20;6(14):3612–23. doi: 10.1039/d4na00151f (PMC11232548; doi:10.1039/d4na00151f)
Supplement: NA-006-D4NA00151F-s001 [file NA-006-D4NA00151F-s001.pdf]

Electronic Supplementary Material (ESI) for Nanoscale Advances.
This journal is © The Royal Society of Chemistry 2024

Supporting Information

Pd NPs decorated on crosslinked sodium alginate modified Iron-based metal-organic framework Fe(BTC) as a green multifunctional catalyst for the oxidative amidation

Samaneh Koosha[a], Ramin Ghorbani-Vaghei[a]*, Sedigheh Alavinia[a], Rahman Karimi-Nami[b], Idris Karakaya[c]

[a]Department of Organic Chemistry, Faculty of Chemistry and Petroleum Sciences, Bu-Ali Sina University, 6517838683, Hamadan, Iran.

[b]Department of Chemistry, Faculty of Science, University of Maragheh, Maragheh, Iran.

[c]Department of Chemistry, College of Basic Sciences, Gebze Technical University, 41400 Gebze, Turkey.

Corresponding Author: rgvaghei@yahoo.com & ghorbani@basu.ac.ir

General procedure for the one-pot synthesis of secondary amides

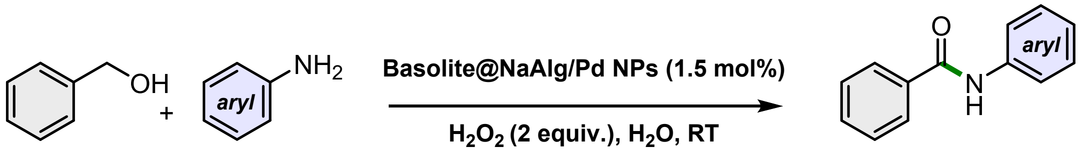

1 2a-o 3a-o

In a standard experimental procedure, the following components were combined in a 25 mL round-bottomed flask: 1.0 mmol of benzyl alcohol, 2.0 mmol of aqueous H_2O_2 (30%), and 1.5 mol% of basolite@SA/ED /Pd NP catalyst in 3 mL of H_2O. The mixture was then stirred at room temperature for 2 hours. After this initial reaction period, 1.0 mmol of aniline was introduced into the reaction mixture. Upon completion of the reaction, the catalyst was separated by centrifugation, and the organic residue was subsequently extracted with ethyl acetate. The solvent was then removed under reduced pressure. Finally, the resulting product was subjected to purification through column chromatography.

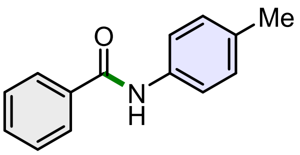

3b

***N*-(*p*-tolyl)benzamide (3b):** ^{1}H NMR (500 MHz, DMSO-d_6, TMS) δ 7.94 (s, 1H), 7.84 (d, J = 7.5 Hz, 2H), 7.55 – 7.48 (m, 3H), 7.44 (t, J = 7.6 Hz, 2H), 7.14 (d, J = 8.2 Hz, 2H), 2.33 (s, 3H). ^{13}C NMR (126 MHz, DMSO-d_6, TMS) δ 166.03, 135.54, 135.45, 135.03, 134.22, 131.68, 130.16, 129.53, 129.39, 128.66, 128.45, 127.14, 120.60, 21.08. FTIR (cm^{-1}) 3310.34 (NH), 1646.90 (C=O).

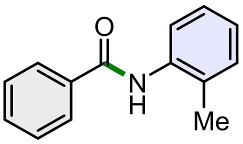

3c

***N*-(*o*-tolyl)benzamide (3c):** ^{1}H NMR (500 MHz, $CDCl_3$, TMS) δ 8.00 – 7.84 (m, 3H), 7.76 (s, 1H), 7.55 (dd, J = 28.5, 5.7 Hz, 3H), 7.32 – 7.21 (m, 2H), 7.15 (d, J = 6.1 Hz, 1H), 1H NMR (500 MHz, CDCl3) δ 7.94 (s, 1H), 7.84 (d, J = 7.5 Hz,2H), 7.56 – 7.48 (m, 3H), 7.44 (t, J = 7.6 Hz, 2H), 7.14 (d, J = 8.2 Hz,2H), 2.36 (s, 3H); ^{13}C NMR (126 MHz, $CDCl_3$, TMS) δ 165.83, 135.79, 134.97, 131.85, 130.50, 129.67, 128.95, 128.83, 128.41, 127.13, 126.87, 125.49, 123.44, 17.87; FTIR (cm^{-1}) 3246.88 (NH), 1649.86 (C=O).

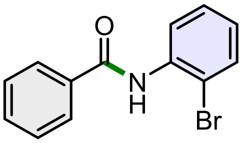

3g

***N*-(2-bromophenyl)benzamide (3g):** ^{1}H NMR (500 MHz, $CDCl_3$, TMS) δ 8.56 (d, J = 8.1 Hz, 1H), 8.47 (s, 1H), 7.94 (d, J = 7.5 Hz, 2H), 7.55 (dd, J = 24.2, 7.4 Hz, 4H), 7.38 (t, J = 7.7 Hz, 1H), 7.02 (t, J = 7.6 Hz, 1H); FTIR (cm^{-1}) 3219.29 (NH), 1651.60 (C=O).

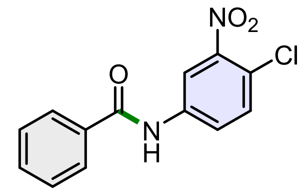

3k

***N*-(4-chloro-3-nitrophenyl)benzamide (3k):** 1H NMR (500 MHz, $CDCl_3$) δ 13.98 (s, 1H), 8.31 (s,1H), 8.07 (s, 1H), 7.98 – 7.75 (m, 3H), 7.57 (dd, J = 29.3, 10.4 Hz, 3H); ^{13}C NMR (126 MHz, DMSO-d_6, TMS) δ 165.89, 147.96, 137.58, 133.69, 132.65, 132.28, 129.06, 127.12, 124.29, 121.85, 116.80; FTIR (cm^{-1}) 3406.99 (NH), 1683.95 (C=O).

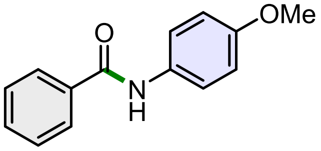

3d

***N*-(4-methoxyphenyl)benzamide (3d):** ^1H NMR (500 MHz, $CDCl_3$, TMS) δ 8.12 (s, 1H), 7.85 (s,2H), 7.53 (d, J = 0.8 Hz, 2H), 7.42 (d, J = 28.3 Hz, 3H), 6.82 (d, J = 4.1 Hz, 2H), 3.74 (s, 3H); ^{13}C NMR (126 MHz, $CDCl_3$, TMS) δ 165.71, 156.54, 135.00, 131.64, 131.22, 128.67, 127.18, 122.25, 114.16, 55.34; FTIR (cm^{-1}) 3330.69 (NH), 1646.99 (C=O).

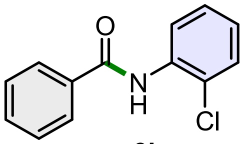

3h

***N*-(2-chlorophenyl)benzamide (3h):** ^1H NMR (500 MHz, $CDCl_3$, TMS) δ 8.45 (d, *J* = 8.2 Hz, 1H), 8.36 (s, 1H), 7.82 (d, *J* = 7.5 Hz, 2H), 7.44 (dt, *J* = 15.0, 7.3 Hz, 3H), 7.30 (d, *J* = 8.0 Hz, 1H), 7.22 (t, *J* = 7.8 Hz, 1H), 6.97 (t, *J* = 7.7 Hz, 1H); FTIR (cm^{-1}) 3226.77 (NH), 1653.24 (C=O).

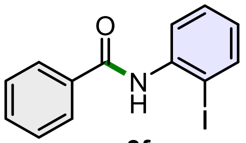

3f

***N*-(2-iodophenyl)benzamide (3f):** 1H NMR (500 MHz, $CDCl_3$) δ 8.37 (d, J = 7.5 Hz, 1H), 8.22 (s, 1H), 7.89 (d, J = 7.4 Hz, 2H), 7.73 (d, J = 7.9 Hz, 1H), 7.50 (t, J = 7.4 Hz, 1H), 7.44 (t, J = 7.5 Hz, 2H), 7.32 (t, J = 7.7 Hz, 1H), 6.80 (t, J = 7.6 Hz, 1H);^{13}C NMR (126 MHz, $CDCl_3$, TMS) δ

165.38, 138.87, 138.31, 134.48, 132.03, 129.42, 128.99, 127.25, 126.18, 122.05, 90.63; FTIR (cm^{-1}) 3215.80 (NH), 1649.33 (C=O).

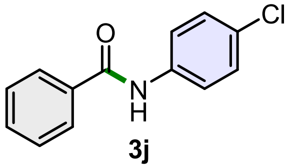

3j

***N*-(4-chlorophenyl)benzamide (3j):** ^{1}H NMR (500 MHz, $CDCl_3$, TMS) δ 7.79 (s, 2H), 7.51 (d, *J* = 20.1 Hz, 1H), 7.43 (m, 3H), 7.27 (d, *J* = 3.7 Hz, 3H), 7.19 (s,1H); FTIR (cm^{-1}) 3349.44 (NH), 1654.64 (C=O).

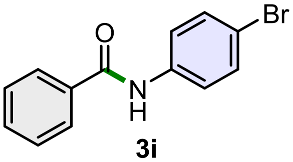

3i

***N*-(4-bromophenyl)benzamide (3i):** ^{1}H NMR (500 MHz, $CDCl_3$, TMS) δ 7.79 (d, *J* = 7.5 Hz, 2H), 7.75 (s, 1H), 7.54 – 7.46 (m, 2H), 7.42 (t, *J* = 9.1 Hz, 3H), 7.19 (s, 1H); ^{13}C NMR (126 MHz, DMSO-d_6, TMS) δ 164.62, 135.99, 133.61, 131.06, 130.88, 127.86, 125.98, 125.83, 120.68; FTIR (cm^{-1}) 3333.19 (NH), 1648.04 (C=O).

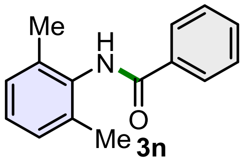

3n

***N*-(2,6-dimethylphenyl)benzamide (3n):** ^{1}H NMR (500 MHz, $CDCl_3$, TMS) δ 7.82 (d, *J* = 7.4 Hz, 2H), 7.47 (t, *J* = 7.3 Hz, 2H), 7.39 (t, *J* = 7.6 Hz, 2H), 7.06 (dd, *J* = 8.9, 5.6 Hz, 1H), 7.03 – 7.01 (m, 2H), 2.18 (s, 6H); ^{13}C NMR (126 MHz, $CDCl_3$, TMS) δ 165.98, 135.60, 134.45, 133.91, 131.76, 128.72, 128.25, 127.40, 127.24, 18.45; FTIR (cm^{-1}) 3274.83 (NH), 1644.05 (C=O).

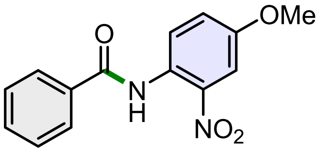

3m

***N*-(4-chloro-3-nitrophenyl)benzamide (3m):** ^{1}H NMR (500 MHz, $CDCl_3$, TMS) δ 11.01 (s, 1H), 8.81 (d, *J* = 9.3 Hz, 1H), 7.90 (d, *J* = 7.5 Hz, 2H), 7.64 (d, *J* = 2.6 Hz, 1H), 7.51 (t, *J* = 7.2 Hz,

1H), 7.45 (t, *J* = 7.5 Hz, 2H), 7.21 (dd, *J* = 13.9, 7.3 Hz, 1H), 3.80 (s, 3H); ^{13}C NMR(126 MHz, $CDCl_3$, TMS) δ 165.54, 155.02, 137.07, 134.16, 132.48, 129.05, 129.01, 127.29, 123.68, 108.69, 17.93; FTIR (cm^{-1}) 3335.72 (NH), 1679.45 (C=O).

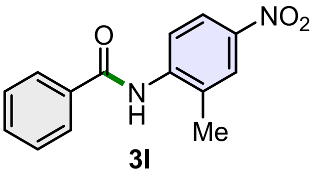

3l

***N*-(2-methyl-4-nitrophenyl)benzamide (3l):** ^{1}H NMR (500 MHz, $CDCl_3$, TMS) δ 8.40 (d, *J* = 8.9 Hz, 1H), 8.07 (dd, *J* = 13.8, 4.8 Hz, 2H), 7.82 (d, *J* = 7.4 Hz, 2H), 7.54 (t, *J* = 7.4 Hz, 1H), 7.46 (t, *J* = 7.6 Hz, 2H), 2.39 (s, 3H); ^{13}C NMR (126 MHz, $CDCl_3$, TMS) δ 165.51, 143.61, 134.21, 132.65, 129.15, 128.78, 127.09, 125.73, 123.03, 120.97, 113.14, 17.90; FTIR (cm^{-1}) 3309.58 (NH), 1651.80 (C=O).

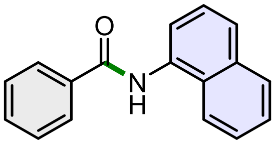

3o

***N*-(naphthalen-1-yl)benzamide (3o):** ^{1}H NMR (500 MHz, $CDCl_3$, TMS) δ 8.20 (s, 1H), 7.88 (d, *J* = 7.2 Hz, 3H), 7.84 – 7.77 (m, 2H), 7.65 (d, *J* = 8.2 Hz, 1H), 7.49 (t, *J* = 7.3 Hz, 1H), 7.46 – 7.36 (m, 4H), 7.17 (s, 1H); ^{13}C NMR (126 MHz, $CDCl_3$, TMS) δ 166.34, 134.85, 134.19, 131.97, 128.88, 128.85, 127.24, 126.43, 126.17, 126.08, 125.79, 121.38, 120.79; FTIR (cm^{-1}) 3235.26 (NH), 1647.10 (C=O).

Spectral Data

^{1}H NMR, ^{13}C NMR, mass and IR spectrum of *N*-(*o*-tolyl)benzamide (3b)

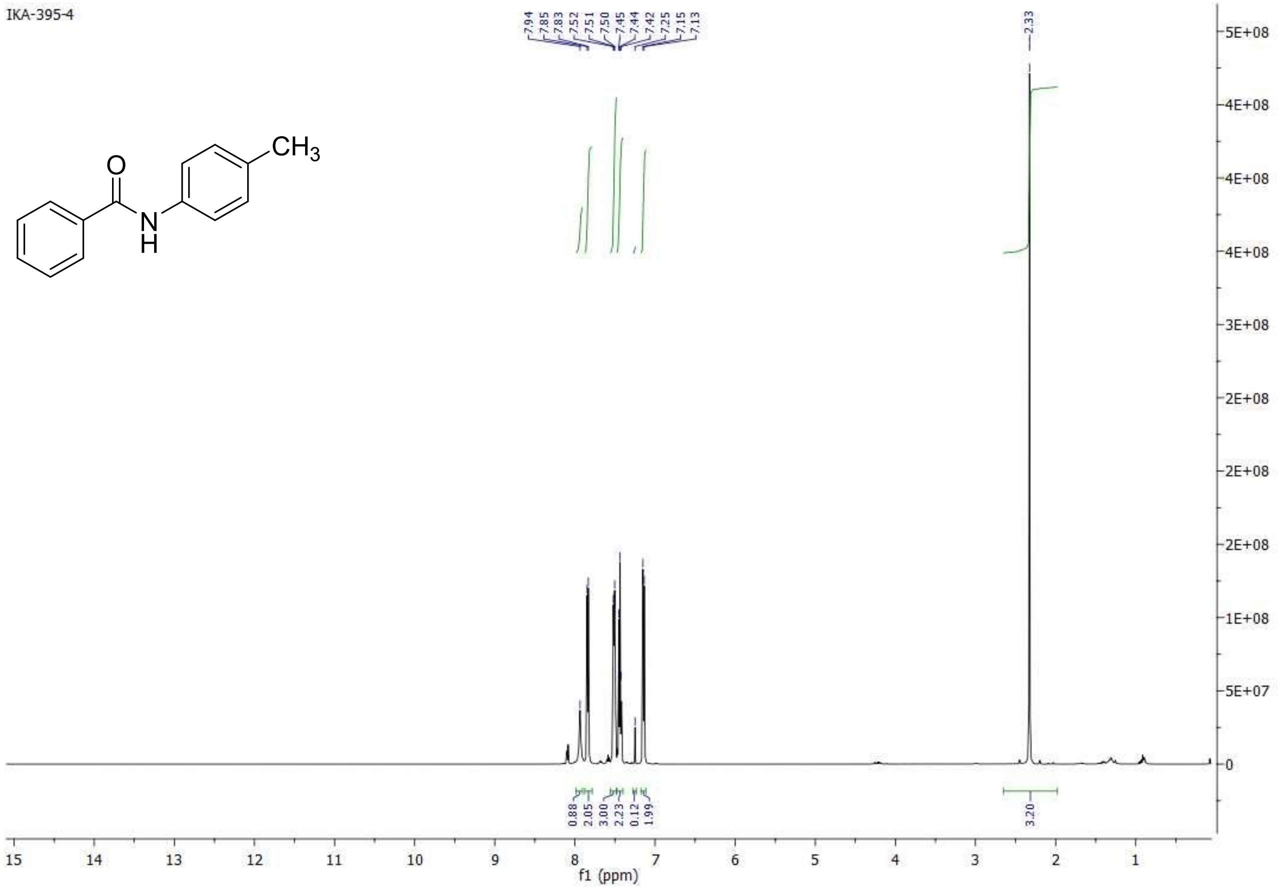

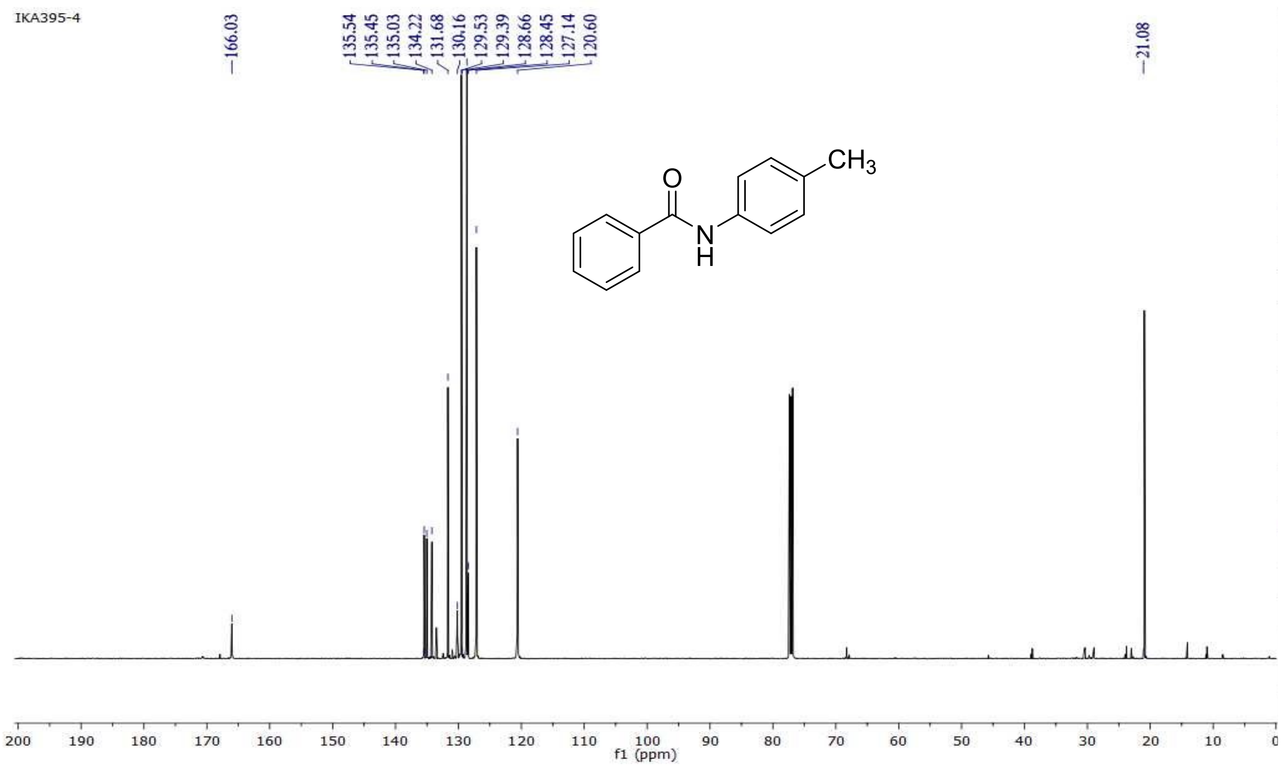
IKA395-4
166.03
135.54
135.45
135.03
134.22
131.68
130.16
129.53
129.39
128.66
128.45
127.14
120.60
21.08
O
N
H
CH3
f1 (ppm)

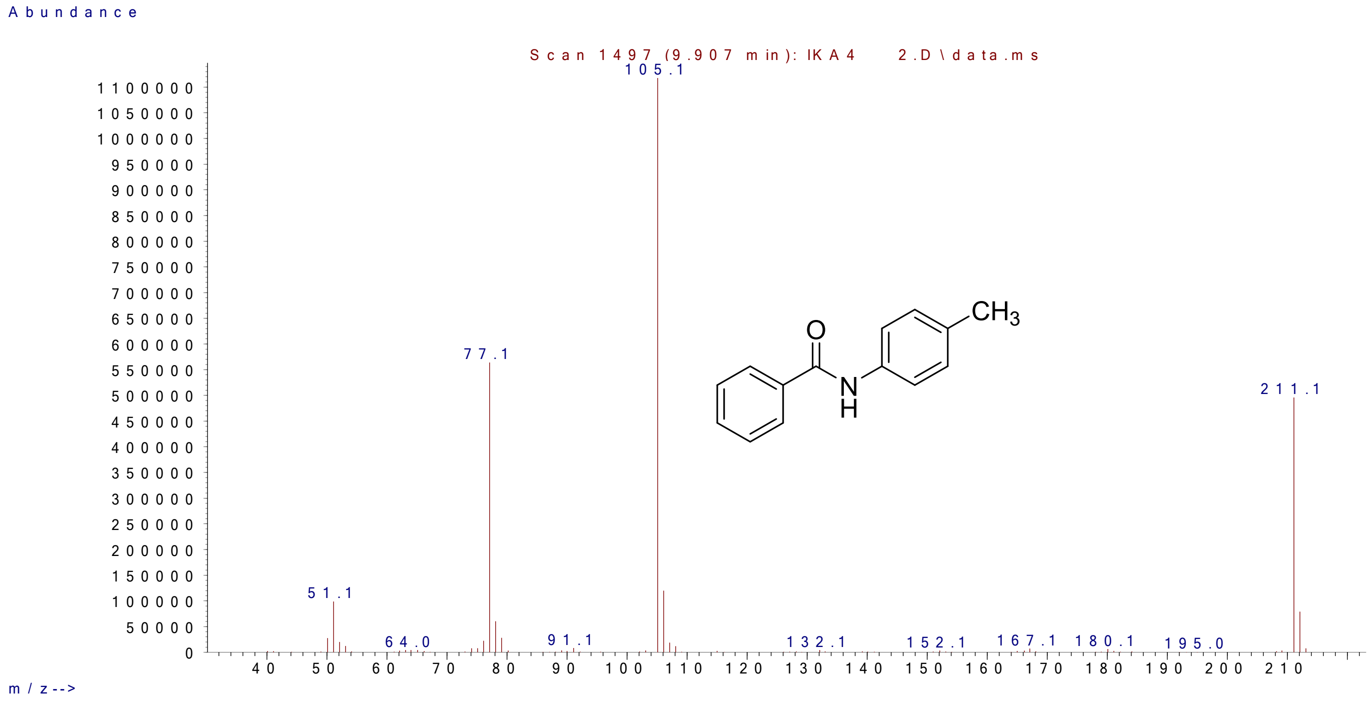
Abundance
Scan 1497 (9.907 min): IKA4 2.D\data.ms
105.1
77.1
211.1
51.1
64.0
91.1
132.1
152.1
167.1
180.1
195.0
m/z-->
O
N
H
CH3

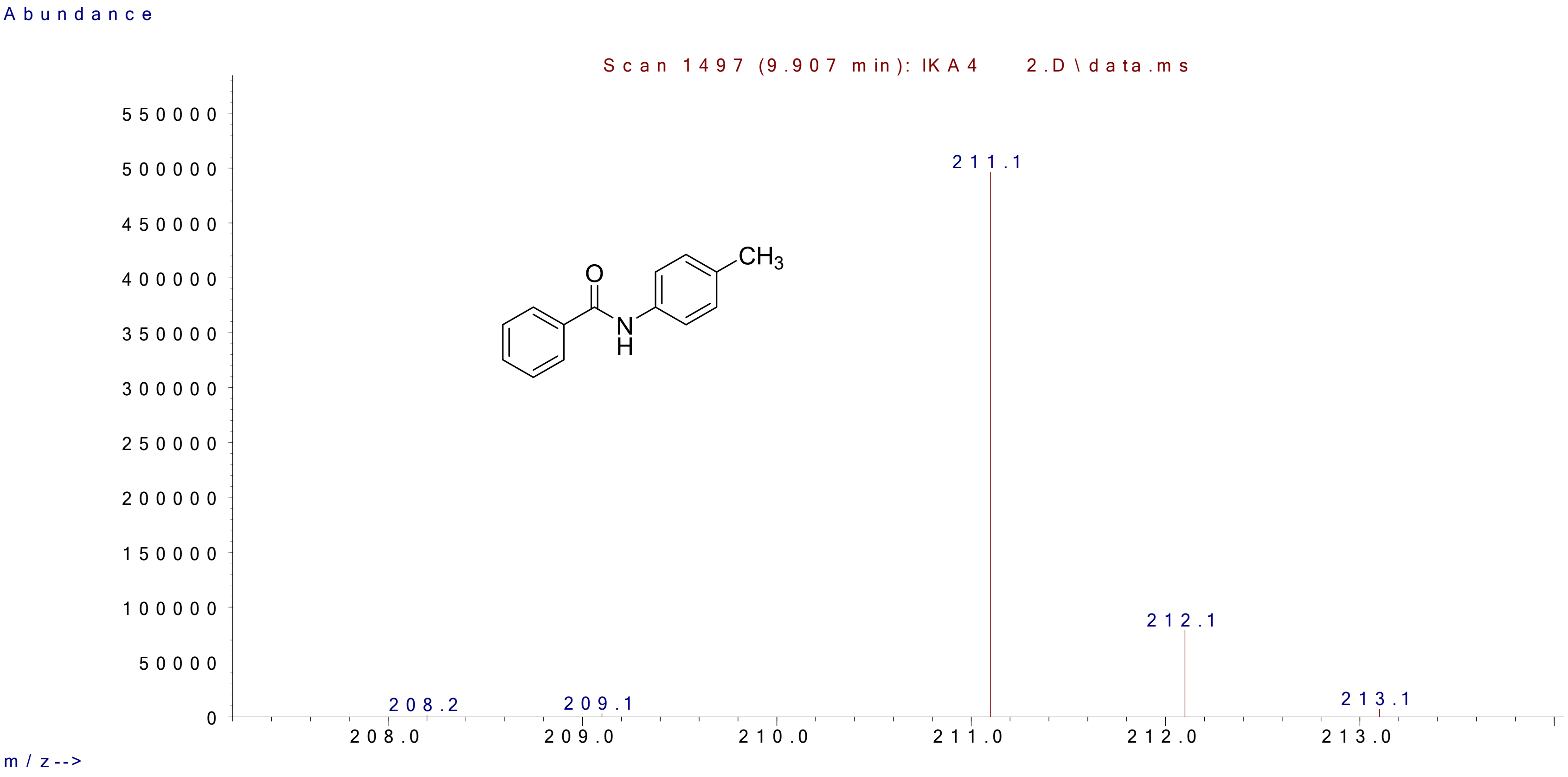
Abundance
Scan 1497 (9.907 min): IKA4 2.D\data.ms
550000
500000
450000
400000
350000
300000
250000
200000
150000
100000
50000
0
211.1
212.1
208.2
209.1
213.1
208.0
209.0
210.0
211.0
212.0
213.0
m/z-->
O
N
H
CH_3

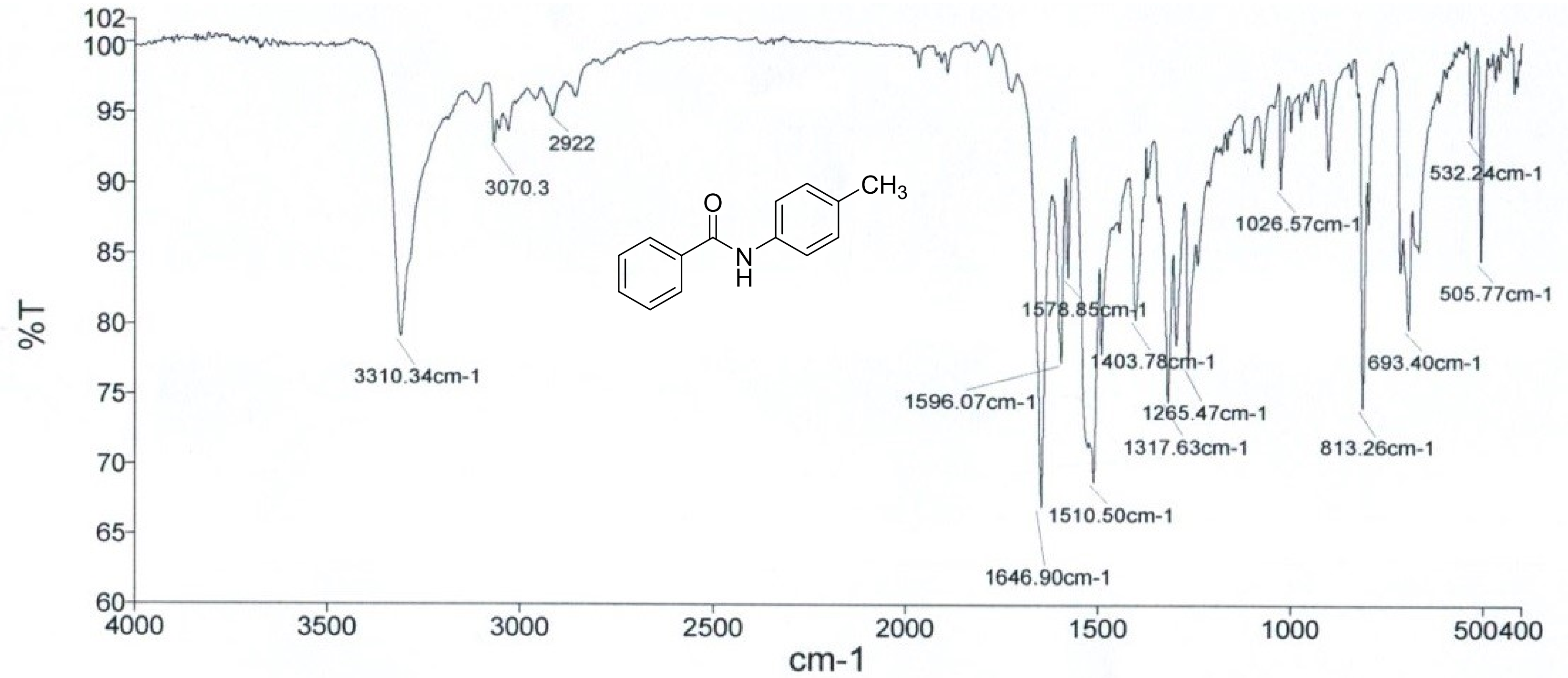

102
100
95
90
85
80
75
70
65
60
%T
4000
3500
3000
2500
2000
1500
1000
500400
cm-1
3310.34cm-1
3070.3
2922
CH3
O
N
H
1578.85cm-1
1596.07cm-1
1646.90cm-1
1510.50cm-1
1403.78cm-1
1265.47cm-1
1317.63cm-1
1026.57cm-1
813.26cm-1
693.40cm-1
532.24cm-1
505.77cm-1

^{1}H NMR, ^{13}C NMR, mass and IR spectrum of *N*-(*o*-tolyl)benzamide (3c)

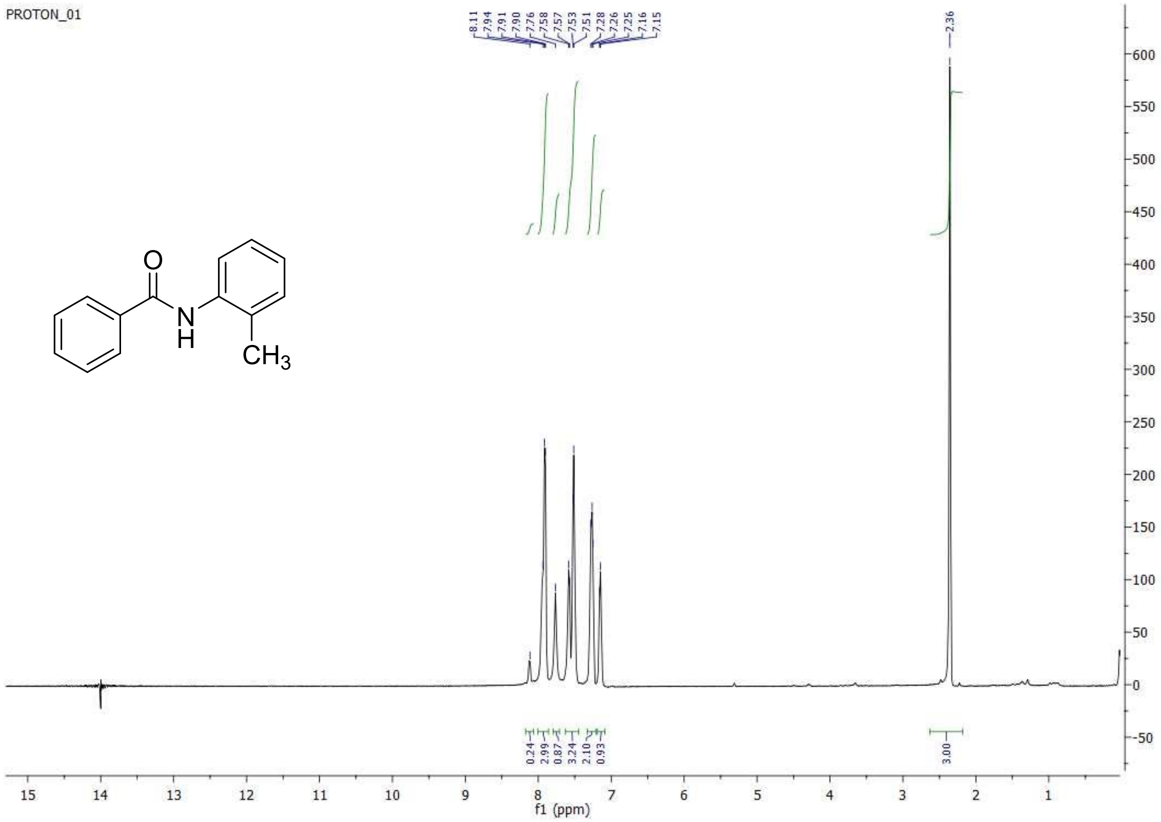

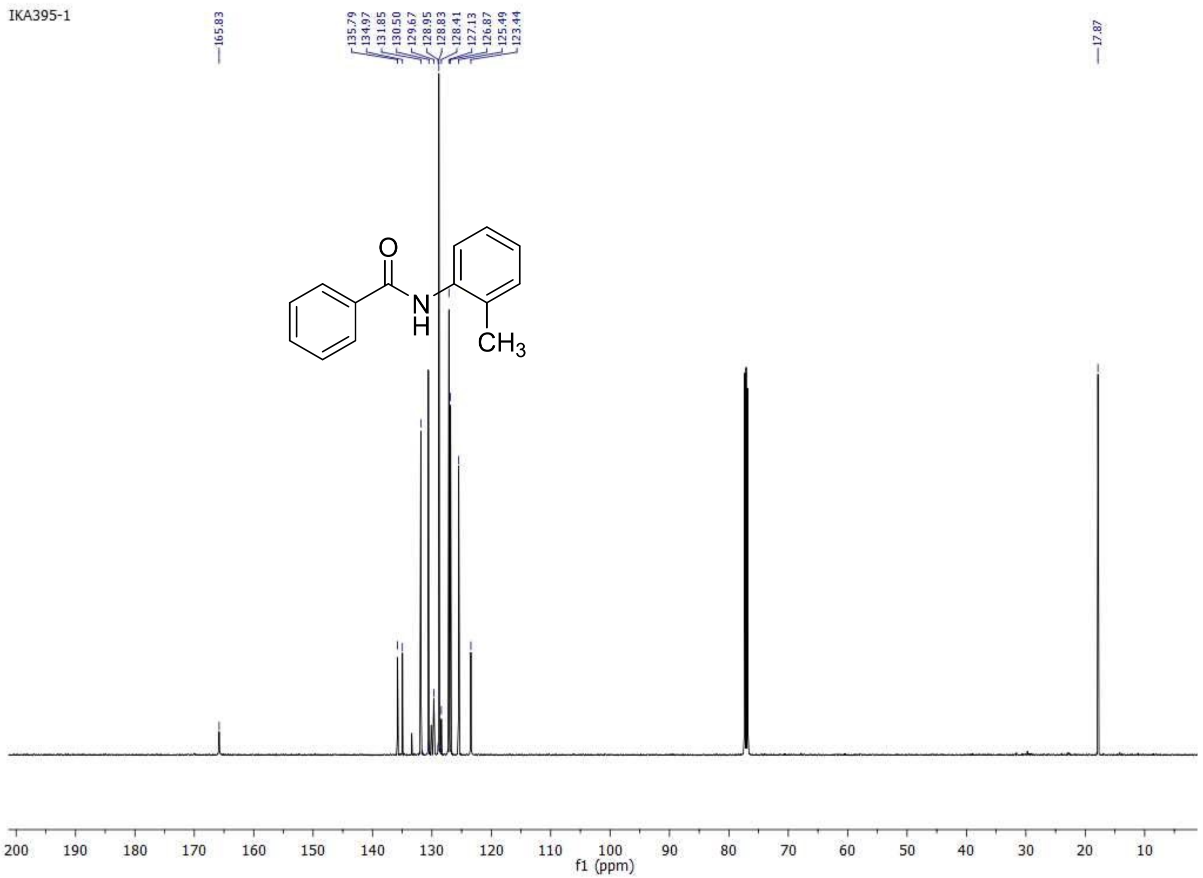
IKA395-1
165.83
135.79
134.97
131.85
130.50
129.67
128.95
128.83
128.41
127.13
126.87
125.49
123.44
17.87
O
N
H
CH3
200 190 180 170 160 150 140 130 120 110 100 90 80 70 60 50 40 30 20 10
f1 (ppm)

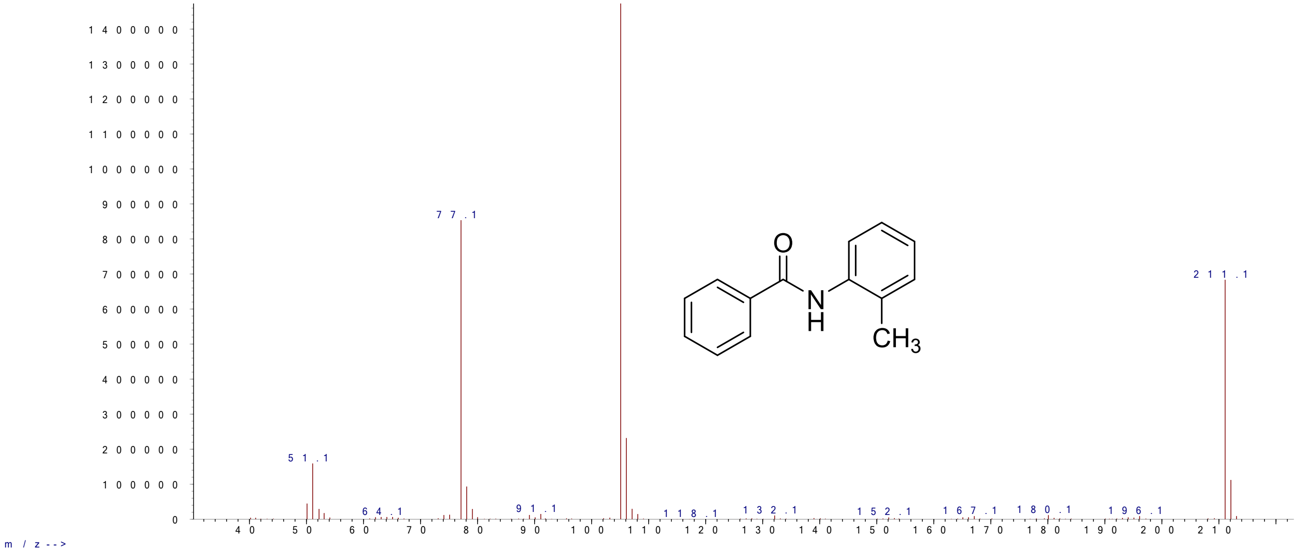
1400000
1300000
1200000
1100000
1000000
900000
800000
700000
600000
500000
400000
300000
200000
100000
0
51.1
64.1
77.1
91.1
118.1
132.1
152.1
167.1
180.1
196.1
211.1
O
N
H
CH3
40 50 60 70 80 90 100 110 120 130 140 150 160 170 180 190 200 210
m/z-->

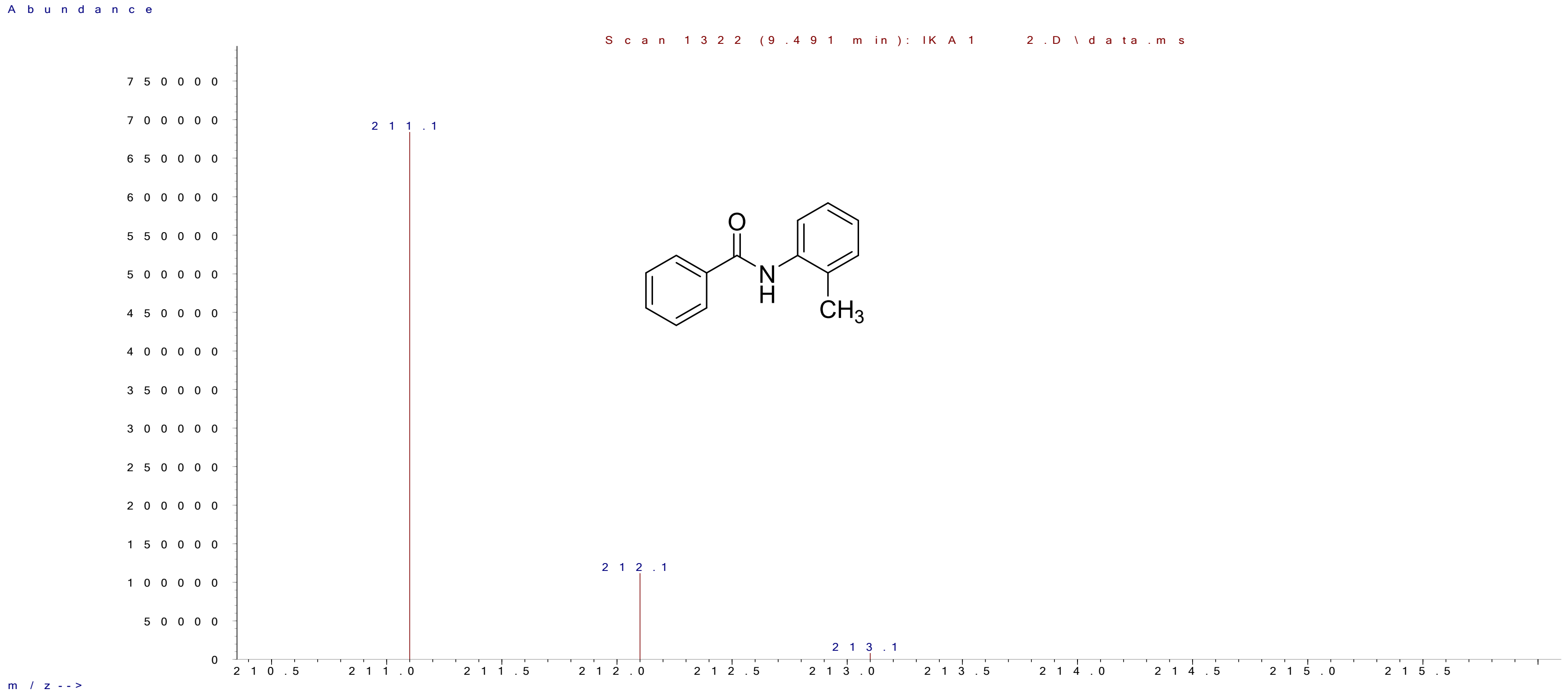
Abundance
Scan 1322 (9.491 min): IKA1 2.D\data.ms
750000
700000
650000
600000
550000
500000
450000
400000
350000
300000
250000
200000
150000
100000
50000
0
211.1
212.1
213.1
O
N
H
CH3
210.5
211.0
211.5
212.0
212.5
213.0
213.5
214.0
214.5
215.0
215.5
m/z-->

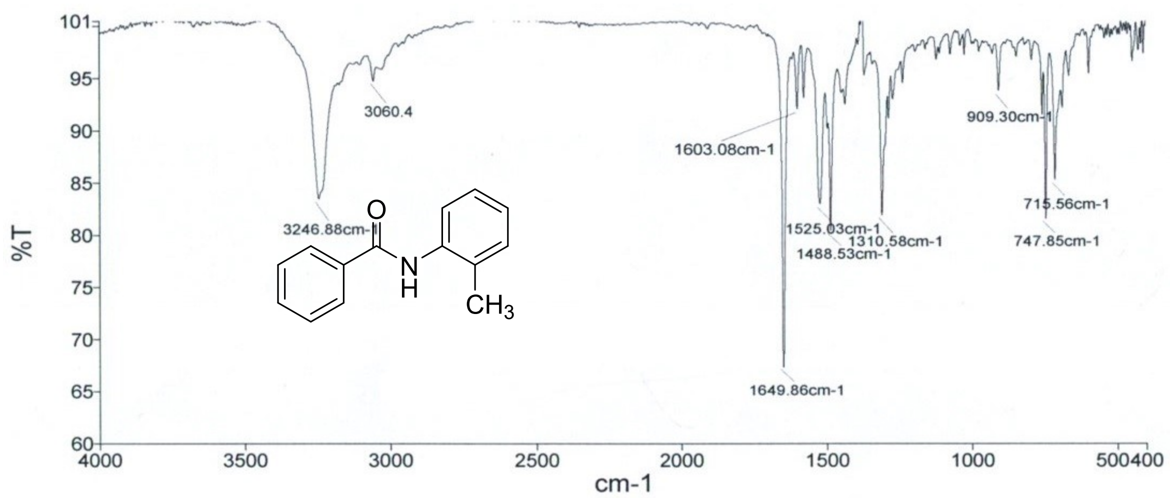
101
95
90
85
80
75
70
65
60
%T
4000
3500
3000
2500
2000
1500
1000
500400
cm-1
3246.88cm-1
3060.4
1603.08cm-1
1649.86cm-1
1525.03cm-1
1488.53cm-1
1310.58cm-1
909.30cm-1
715.56cm-1
747.85cm-1
O
N
H
CH3

^{1}H NMR, mass and IR spectrum of *N*-(2-bromophenyl)benzamide (3g)

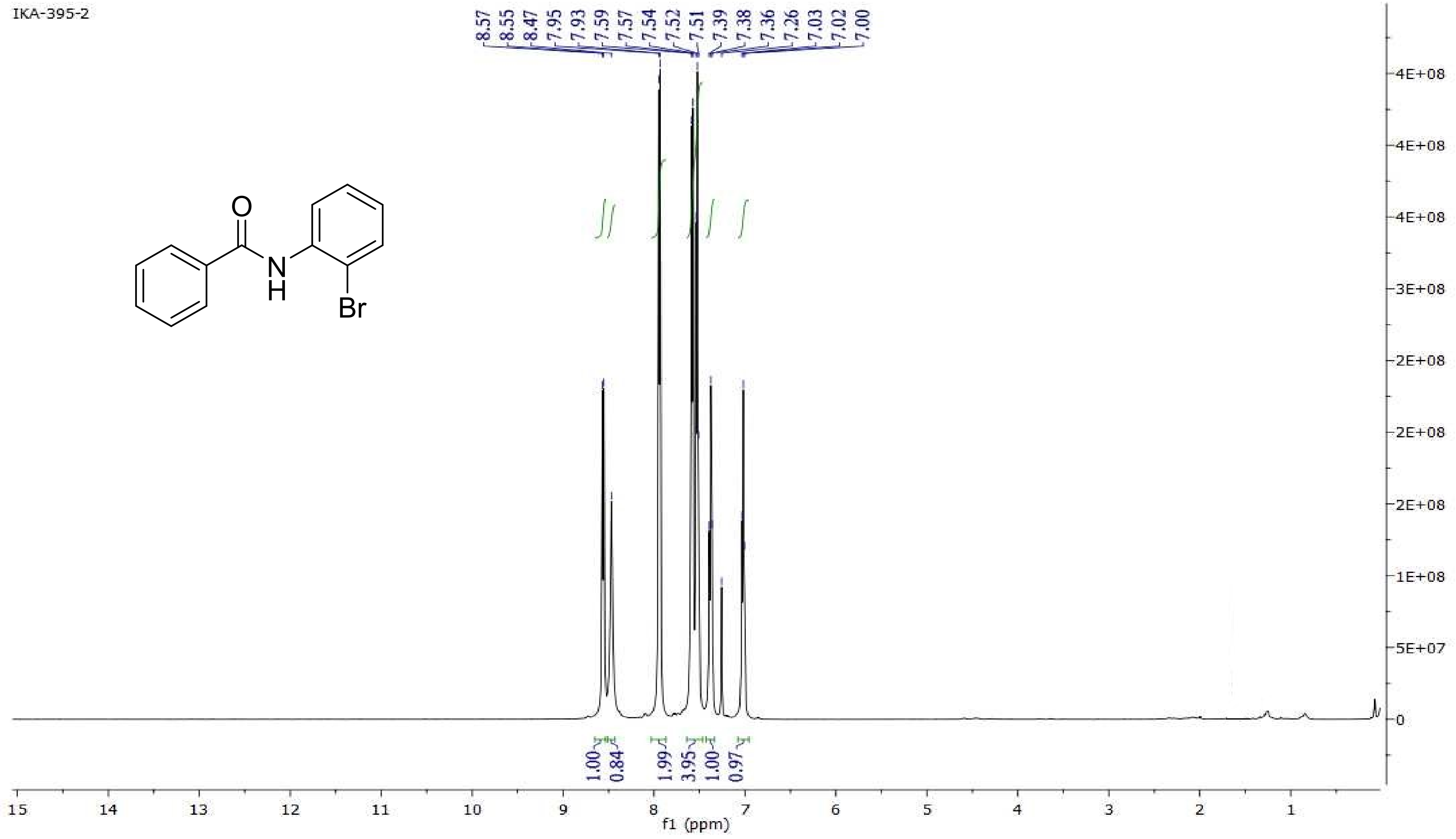

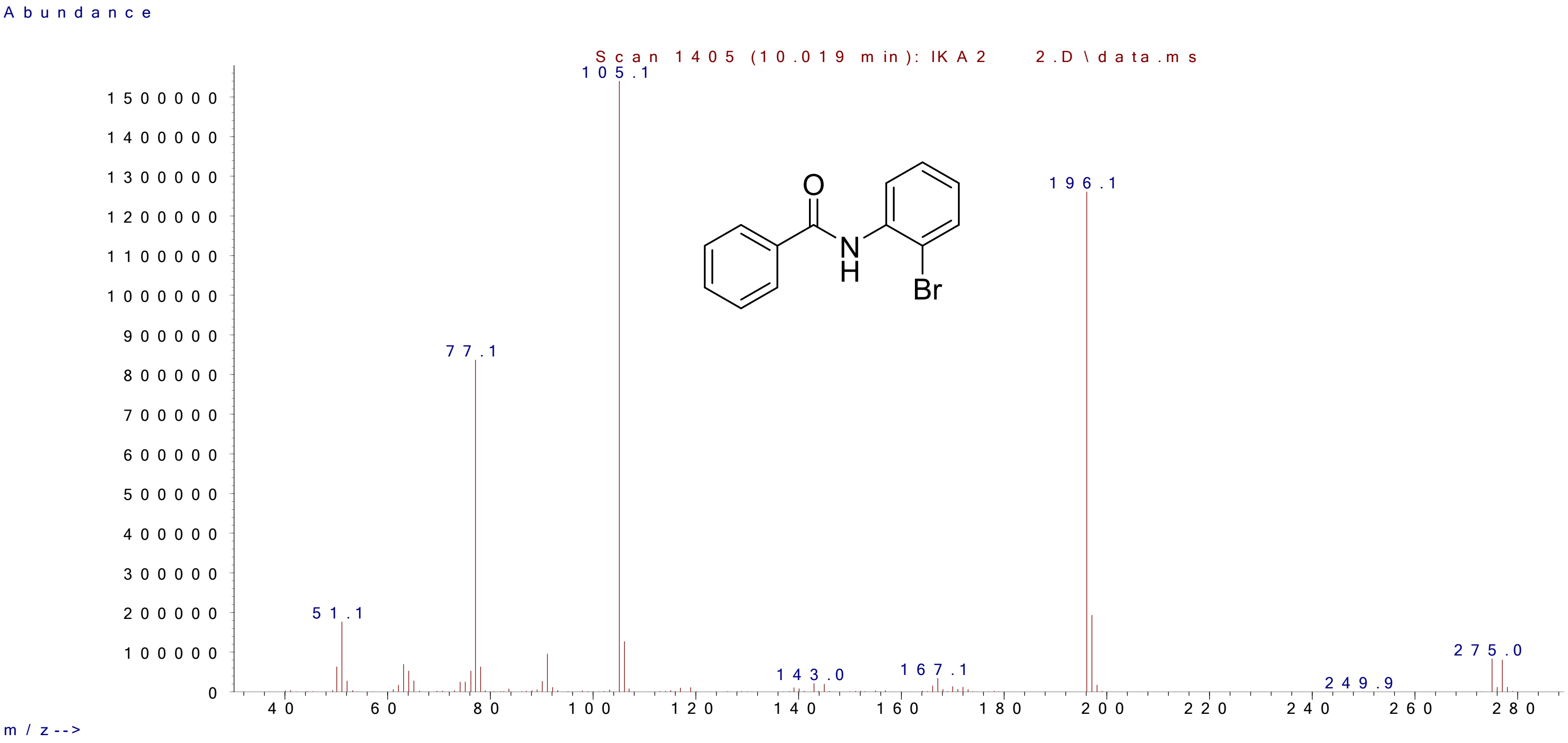
Abundance
Scan 1405 (10.019 min): IKA2 2.D\data.ms
1500000
1400000
1300000
1200000
1100000
1000000
900000
800000
700000
600000
500000
400000
300000
200000
100000
0
105.1
196.1
77.1
51.1
143.0
167.1
249.9
275.0
O
N
H
Br
40 60 80 100 120 140 160 180 200 220 240 260 280
m/z-->

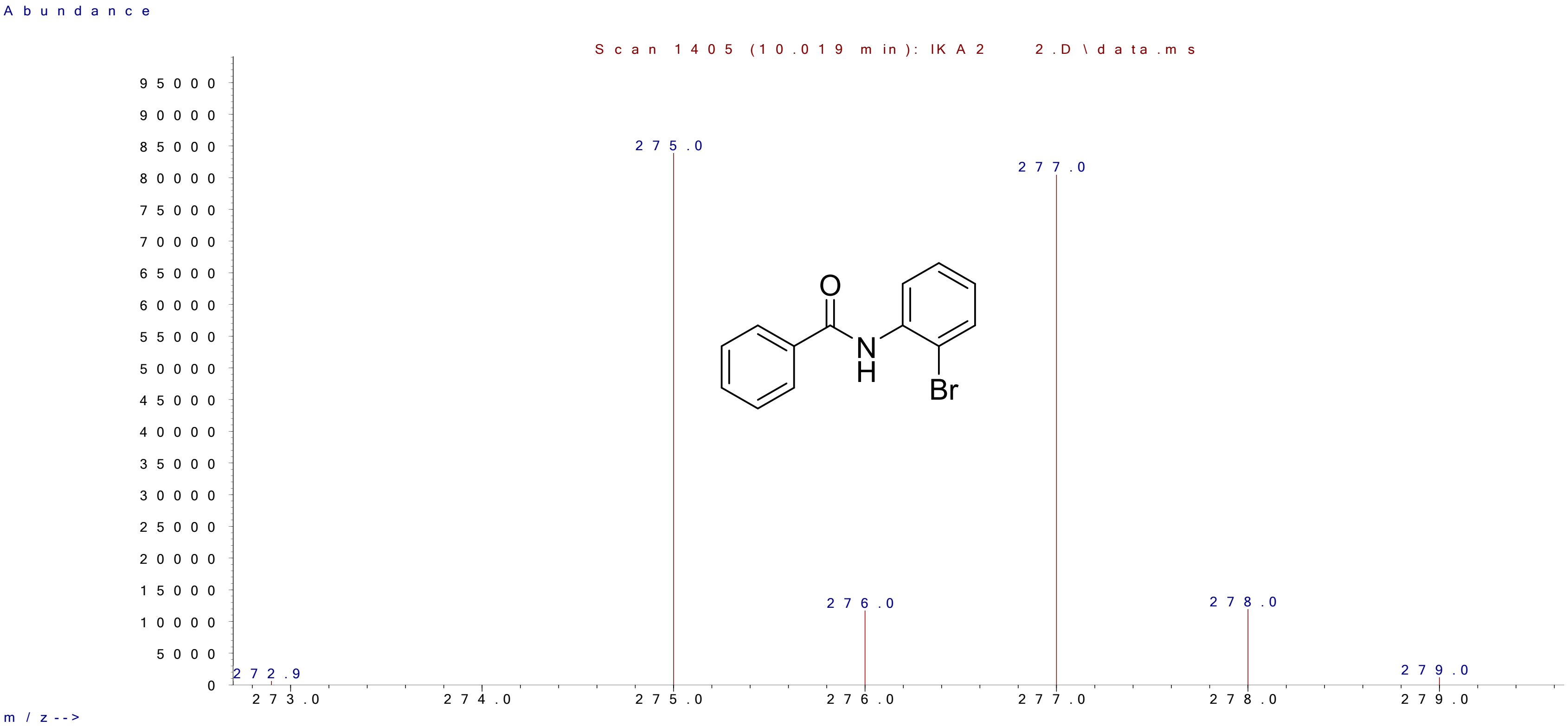
Abundance
Scan 1405 (10.019 min): IKA2 2.D\data.ms
95000
90000
85000
80000
75000
70000
65000
60000
55000
50000
45000
40000
35000
30000
25000
20000
15000
10000
5000
0
275.0
277.0
276.0
278.0
272.9
279.0
O
N
H
Br
273.0
274.0
275.0
276.0
277.0
278.0
279.0
m/z-->

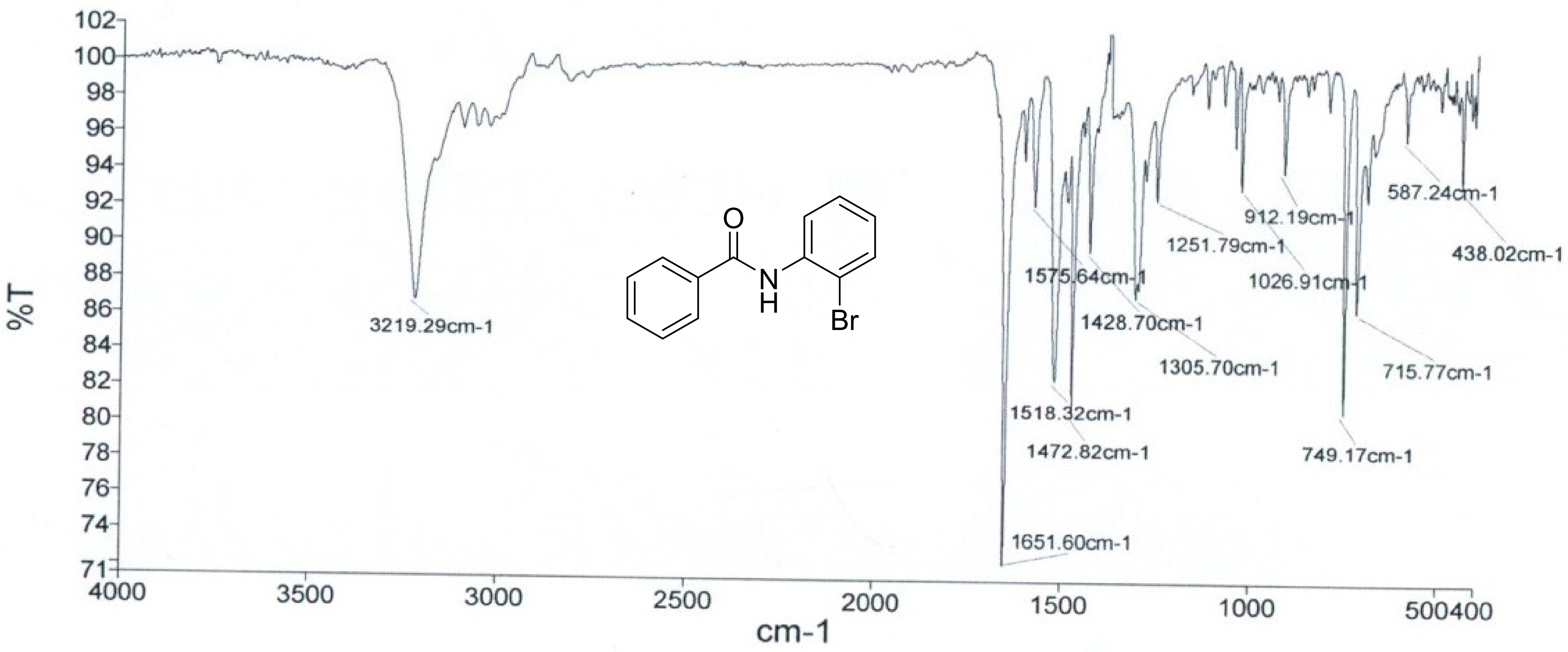

%T
cm-1
3219.29cm-1
1651.60cm-1
1575.64cm-1
1518.32cm-1
1472.82cm-1
1428.70cm-1
1305.70cm-1
1251.79cm-1
1026.91cm-1
912.19cm-1
749.17cm-1
715.77cm-1
587.24cm-1
438.02cm-1
O
N
H
Br

^{1}H NMR, ^{13}C NMR, mass and IR spectrum of *N*-(4-chloro-3-nitrophenyl)benzamide (3k)

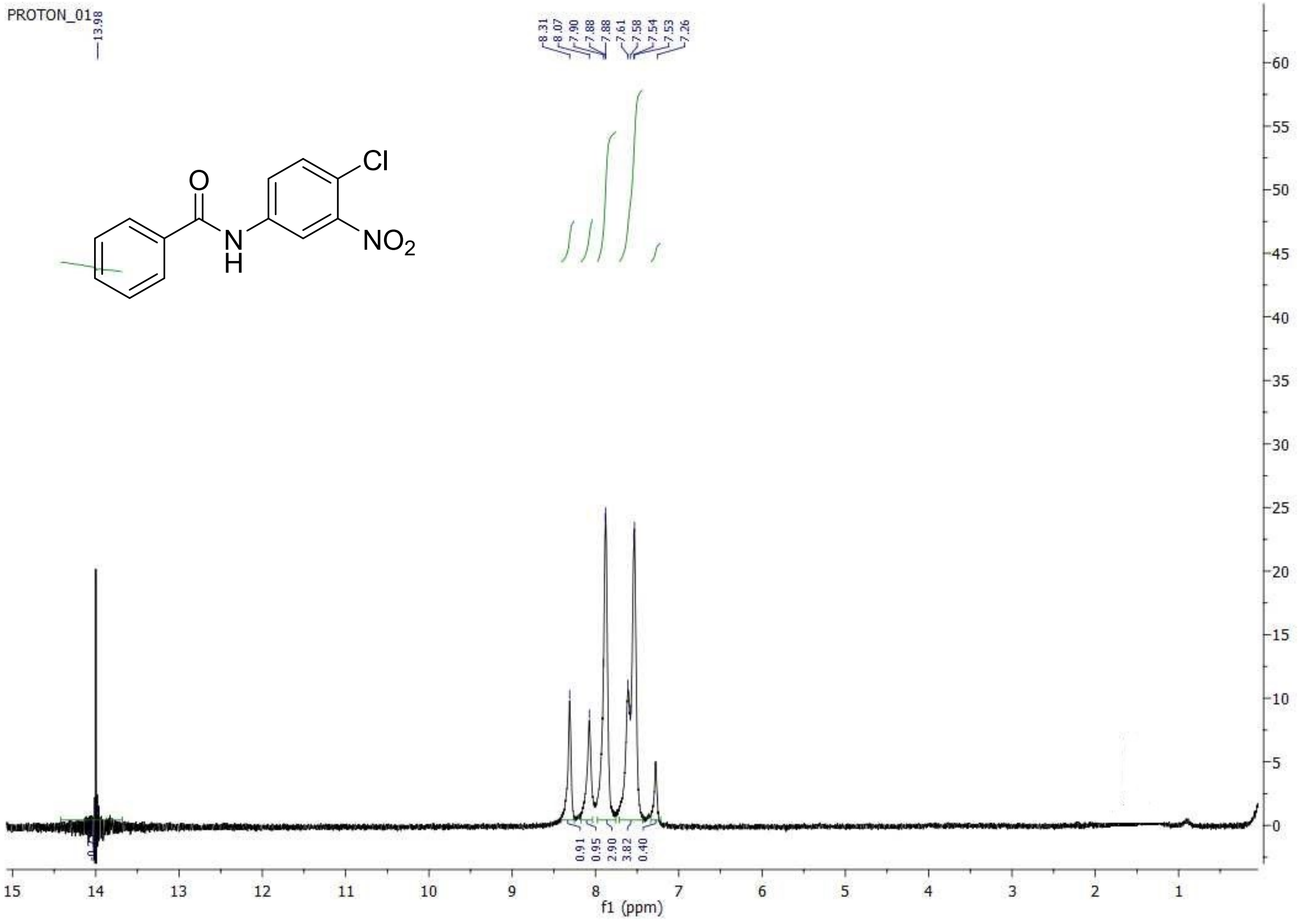

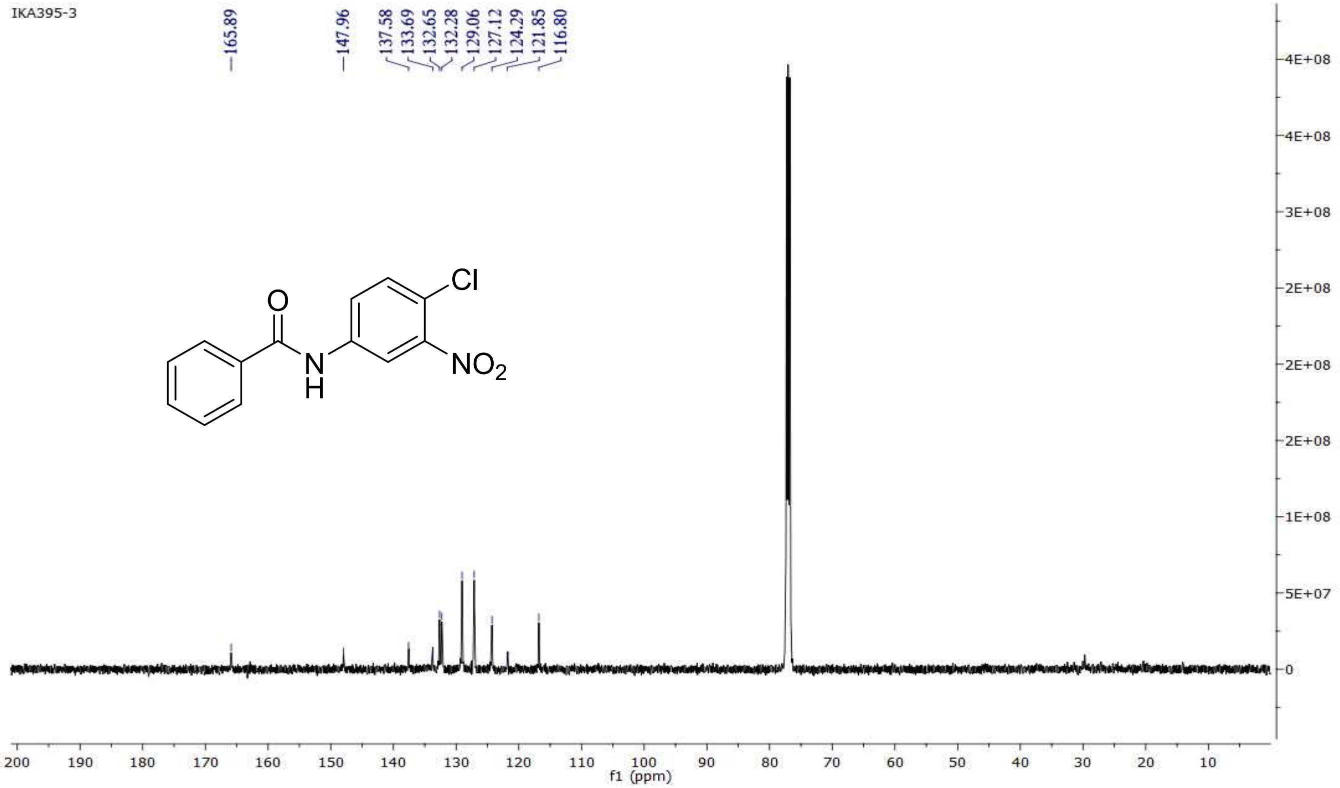
IKA395-3
165.89
147.96
137.58
133.69
132.65
132.28
129.06
127.12
124.29
121.85
116.80
Cl
O
N
H
NO2
4E+08
4E+08
3E+08
2E+08
2E+08
2E+08
1E+08
5E+07
0
200 190 180 170 160 150 140 130 120 110 100 90 80 70 60 50 40 30 20 10
f1 (ppm)

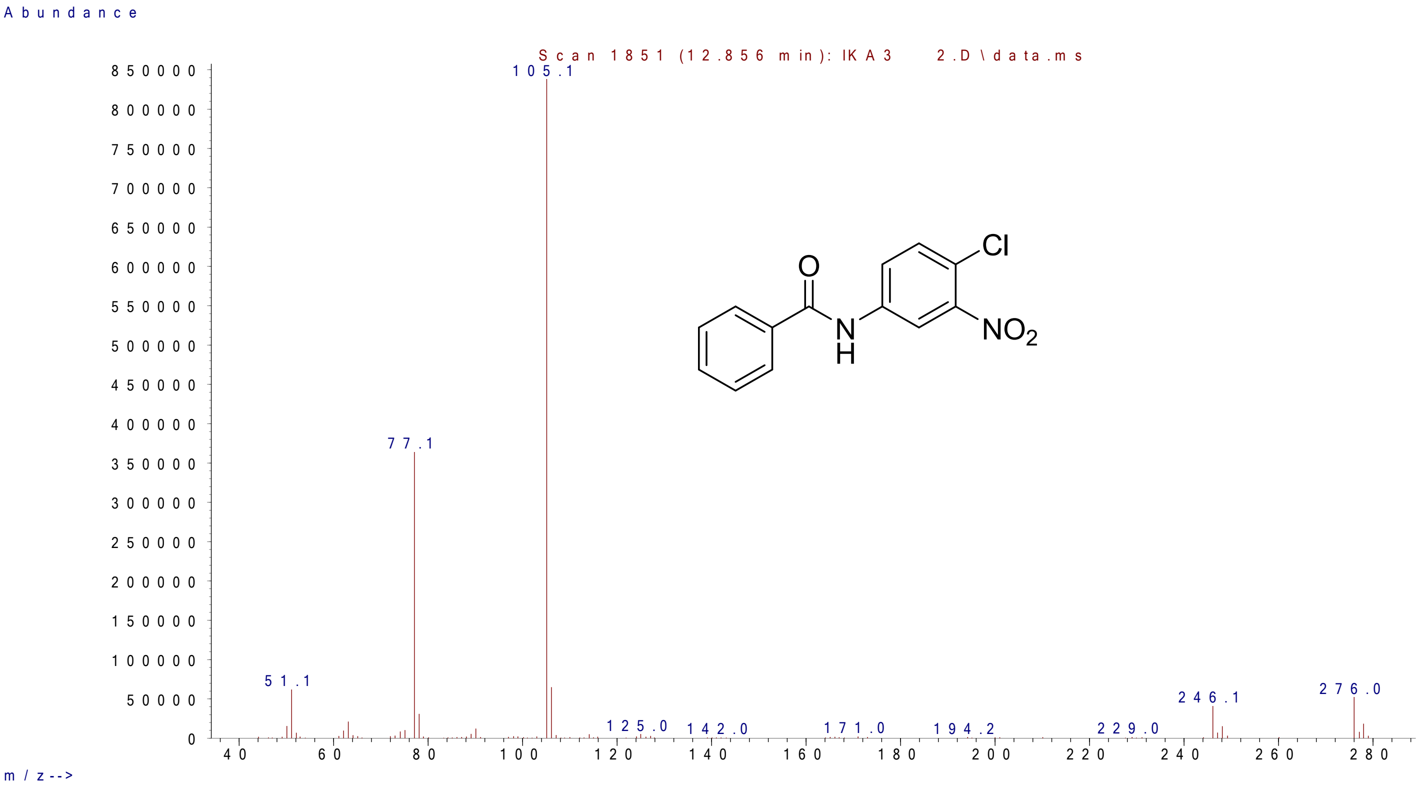
Abundance
Scan 1851 (12.856 min): IKA3 2.D\data.ms
105.1
77.1
51.1
125.0
142.0
171.0
194.2
229.0
246.1
276.0
850000
800000
750000
700000
650000
600000
550000
500000
450000
400000
350000
300000
250000
200000
150000
100000
50000
0
40
60
80
100
120
140
160
180
200
220
240
260
280
m/z-->
O
N
H
Cl
NO2

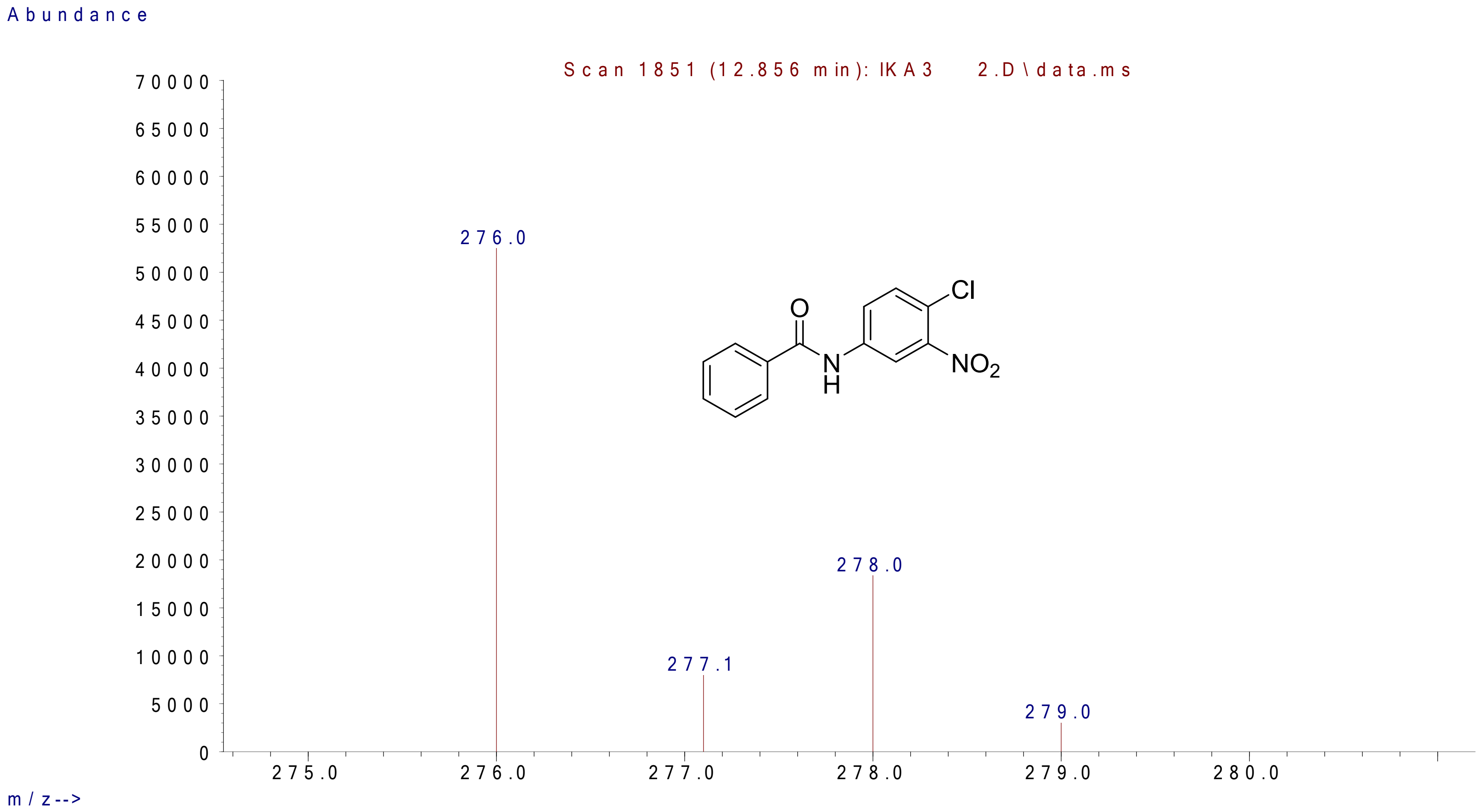

Abundance
Scan 1851 (12.856 min): IKA3 2.D\data.ms
70000
65000
60000
55000
50000
45000
40000
35000
30000
25000
20000
15000
10000
5000
0
276.0
277.1
278.0
279.0
O
N
H
Cl
NO2
275.0
276.0
277.0
278.0
279.0
280.0
m/z-->

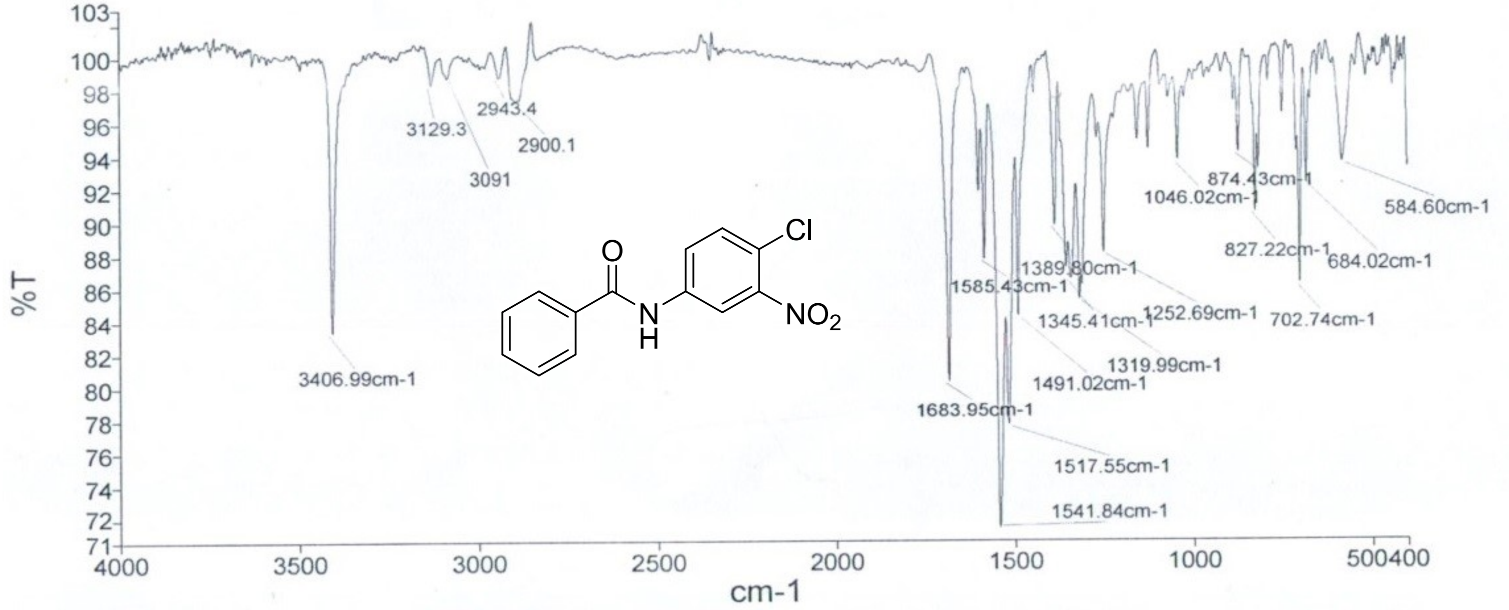

^{1}H NMR, ^{13}C NMR, mass and IR spectrum of *N*-(4-methoxyphenyl)benzamide (3d)

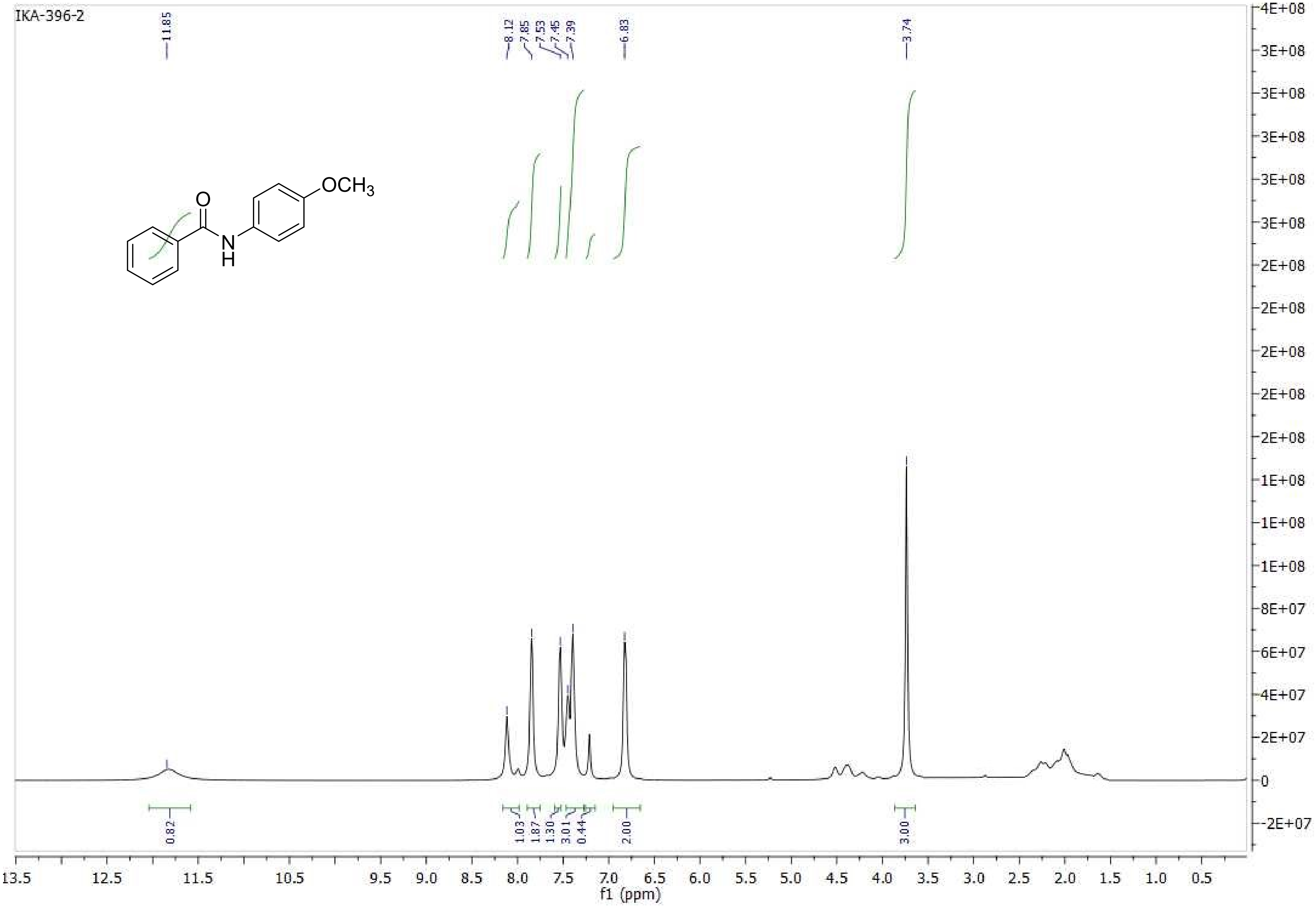
IKA-396-2
11.85
8.12
7.85
7.53
7.45
7.39
6.83
3.74
OCH3
O
N
H
0.82
1.03
1.87
1.30
3.01
0.44
2.00
3.00
f1 (ppm)

IKA396-2

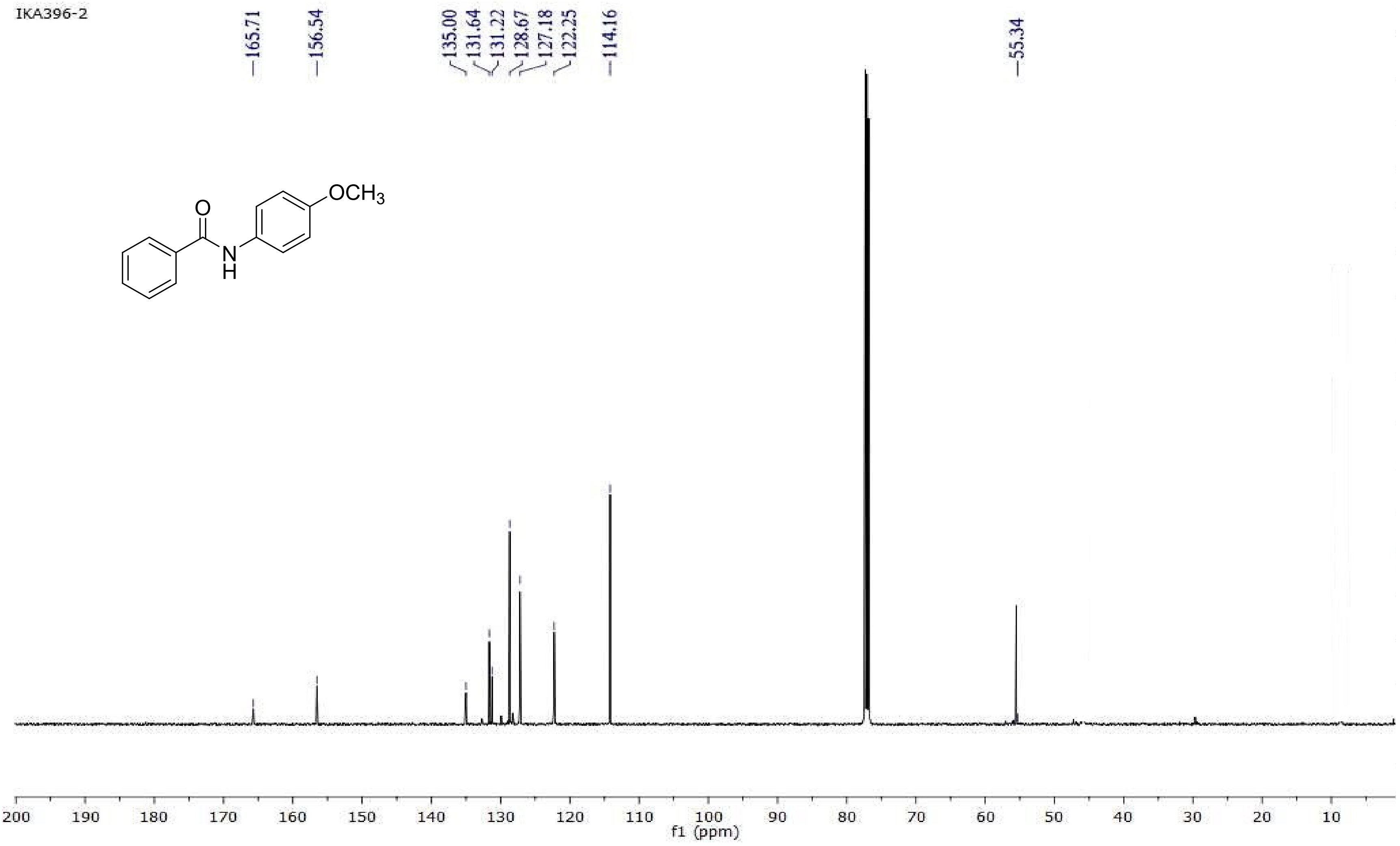

165.71
156.54
135.00
131.64
131.22
128.67
127.18
122.25
114.16
55.34
O
N
H
OCH3
200
190
180
170
160
150
140
130
120
110
100
90
80
70
60
50
40
30
20
10
f1 (ppm)

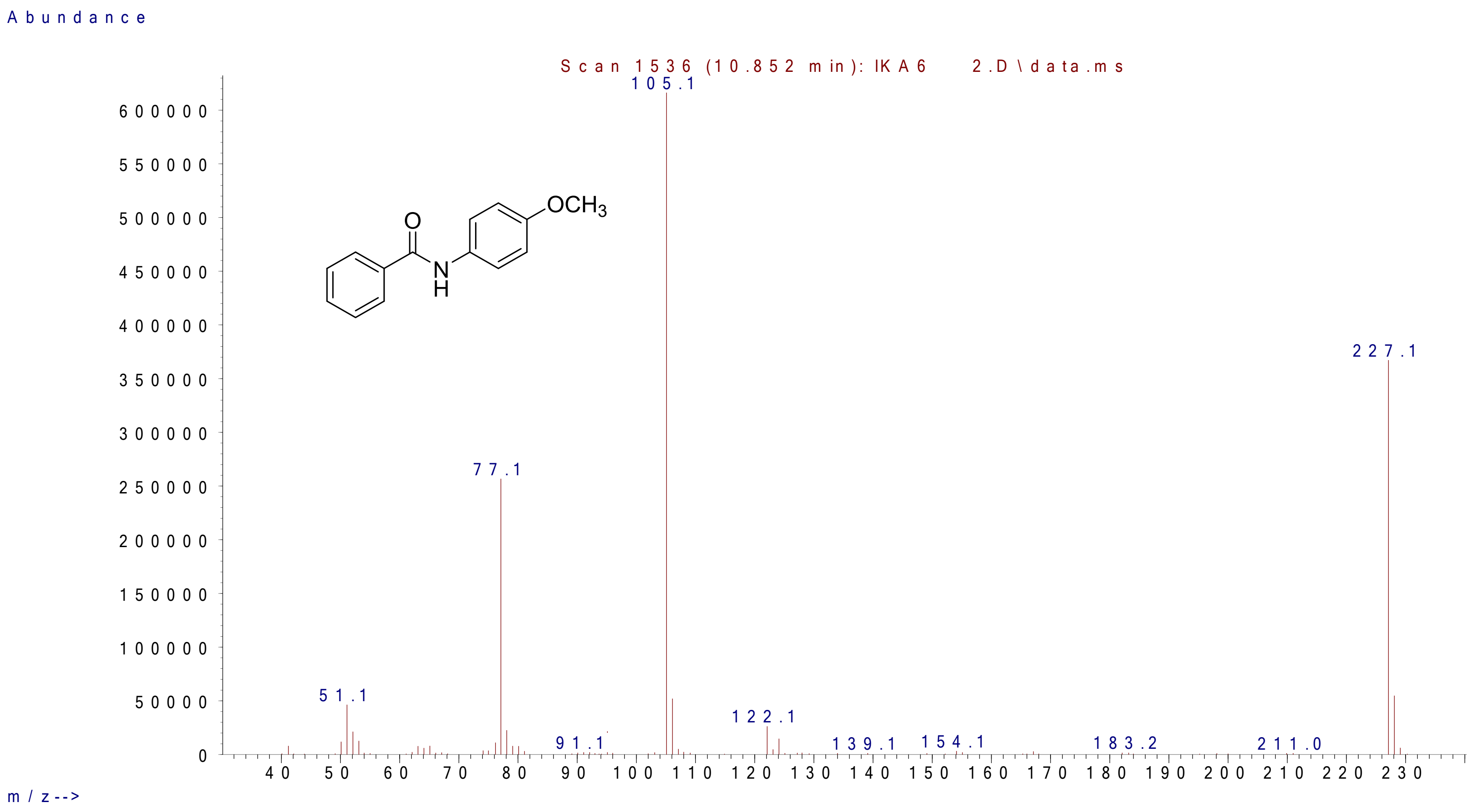
Abundance
Scan 1536 (10.852 min): IKA6 2.D\data.ms
600000
550000
500000
450000
400000
350000
300000
250000
200000
150000
100000
50000
0
OCH3
O
N
H
105.1
227.1
77.1
51.1
91.1
122.1
139.1
154.1
183.2
211.0
40 50 60 70 80 90 100 110 120 130 140 150 160 170 180 190 200 210 220 230
m/z-->

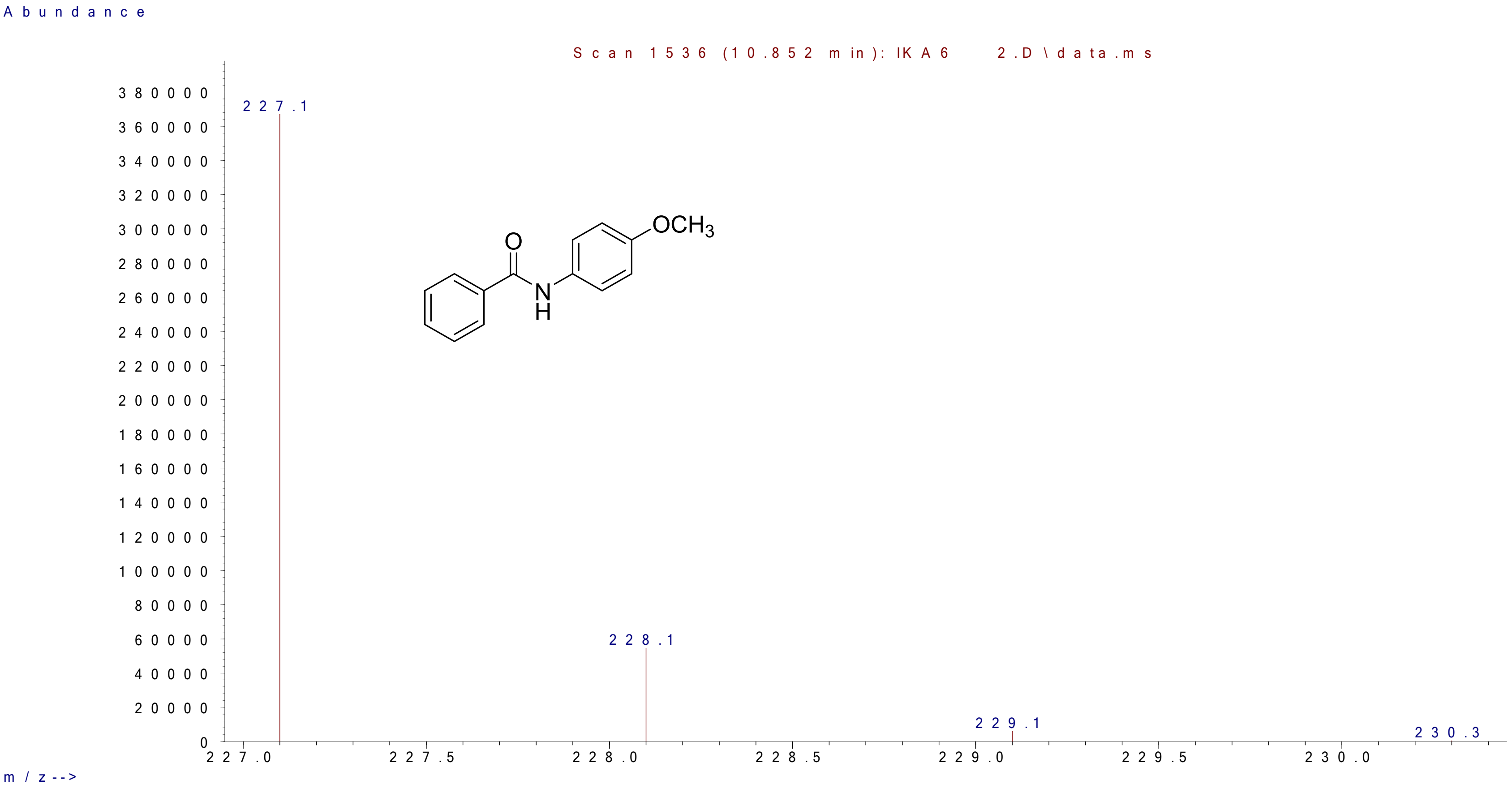

Abundance
Scan 1536 (10.852 min): IKA6 2.D\data.ms
380000
360000
340000
320000
300000
280000
260000
240000
220000
200000
180000
160000
140000
120000
100000
80000
60000
40000
20000
0
227.1
228.1
229.1
230.3
OCH3
O
N
H
227.0
227.5
228.0
228.5
229.0
229.5
230.0
m/z-->

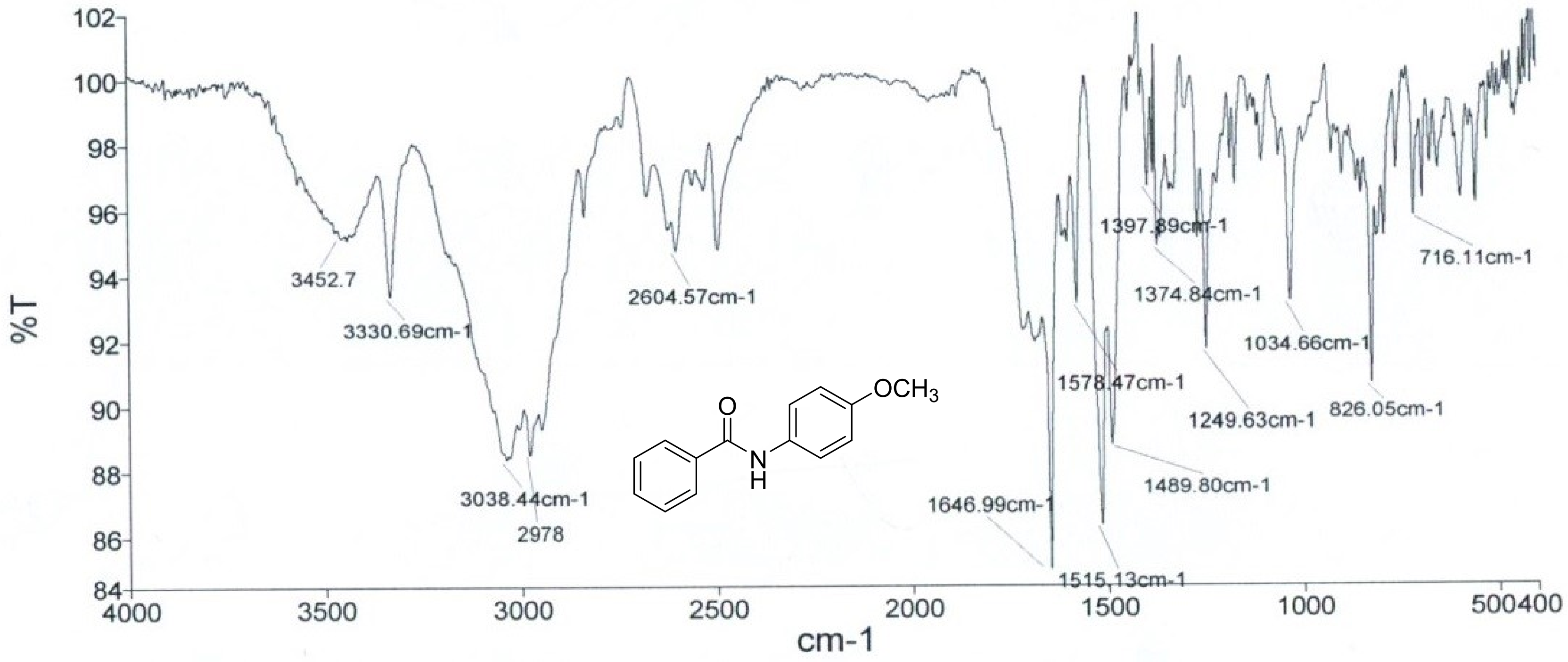

102
100
98
96
94
92
90
88
86
84
%T
4000
3500
3000
2500
2000
1500
1000
500400
cm-1
3452.7
3330.69cm-1
3038.44cm-1
2978
2604.57cm-1
1646.99cm-1
1515.13cm-1
1578.47cm-1
1489.80cm-1
1397.89cm-1
1374.84cm-1
1249.63cm-1
1034.66cm-1
826.05cm-1
716.11cm-1
O
N
H
OCH3

^{1}H NMR, mass and IR spectrum of *N*-(2-chlorophenyl)benzamide (3h)

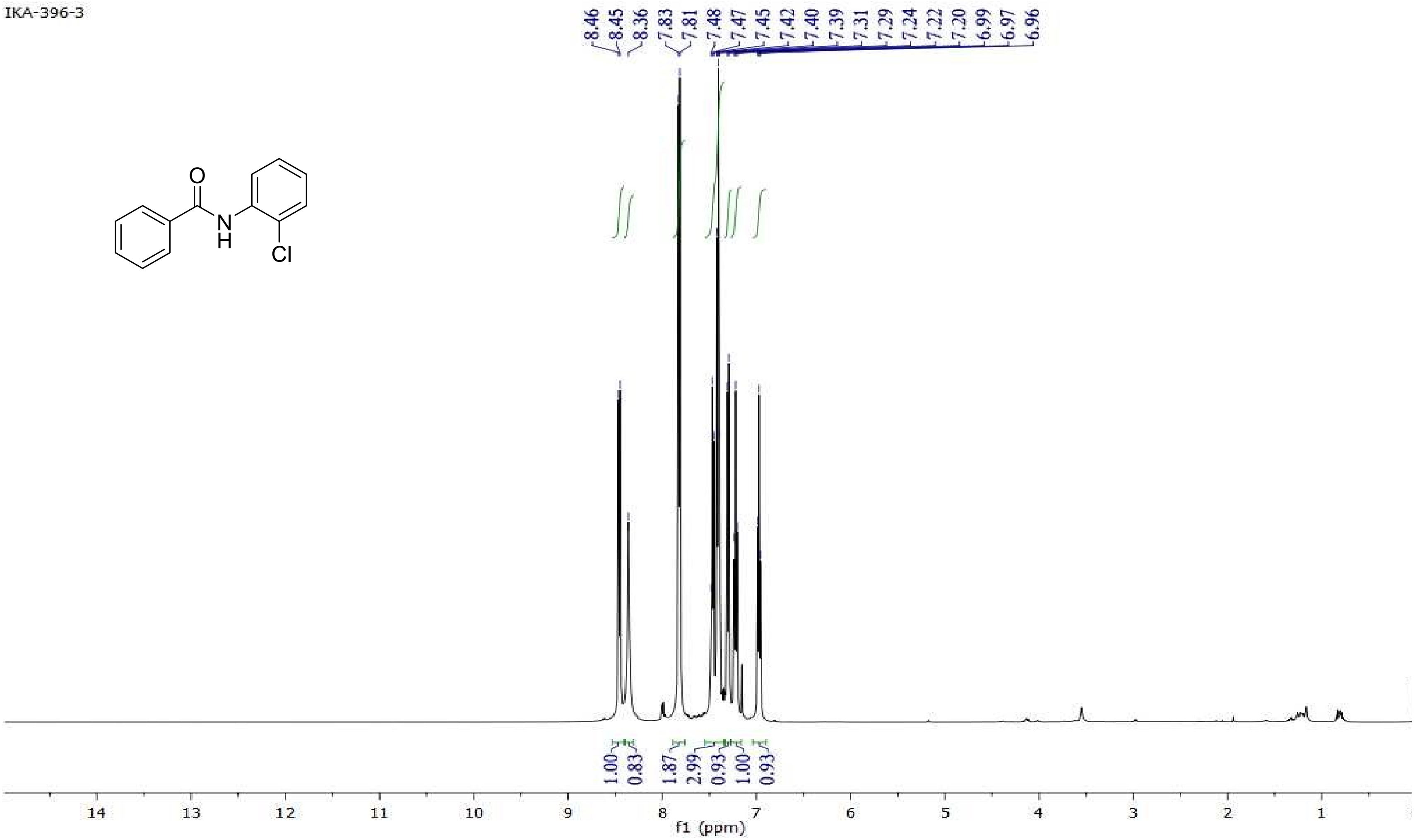

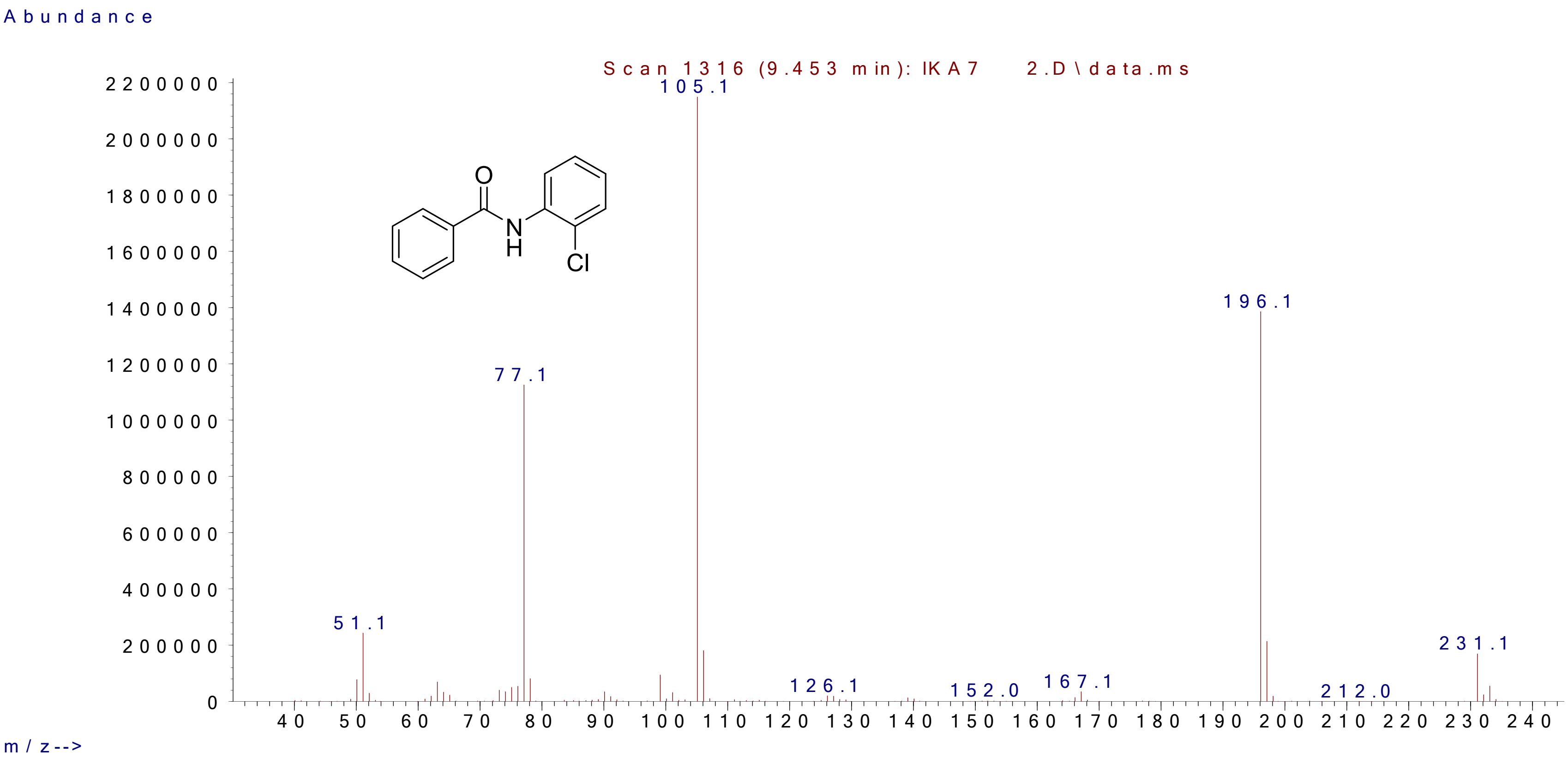

Abundance
Scan 1316 (9.453 min): IKA7 2.D\data.ms
O
N
H
Cl
105.1
196.1
77.1
51.1
231.1
126.1
152.0
167.1
212.0
m/z-->

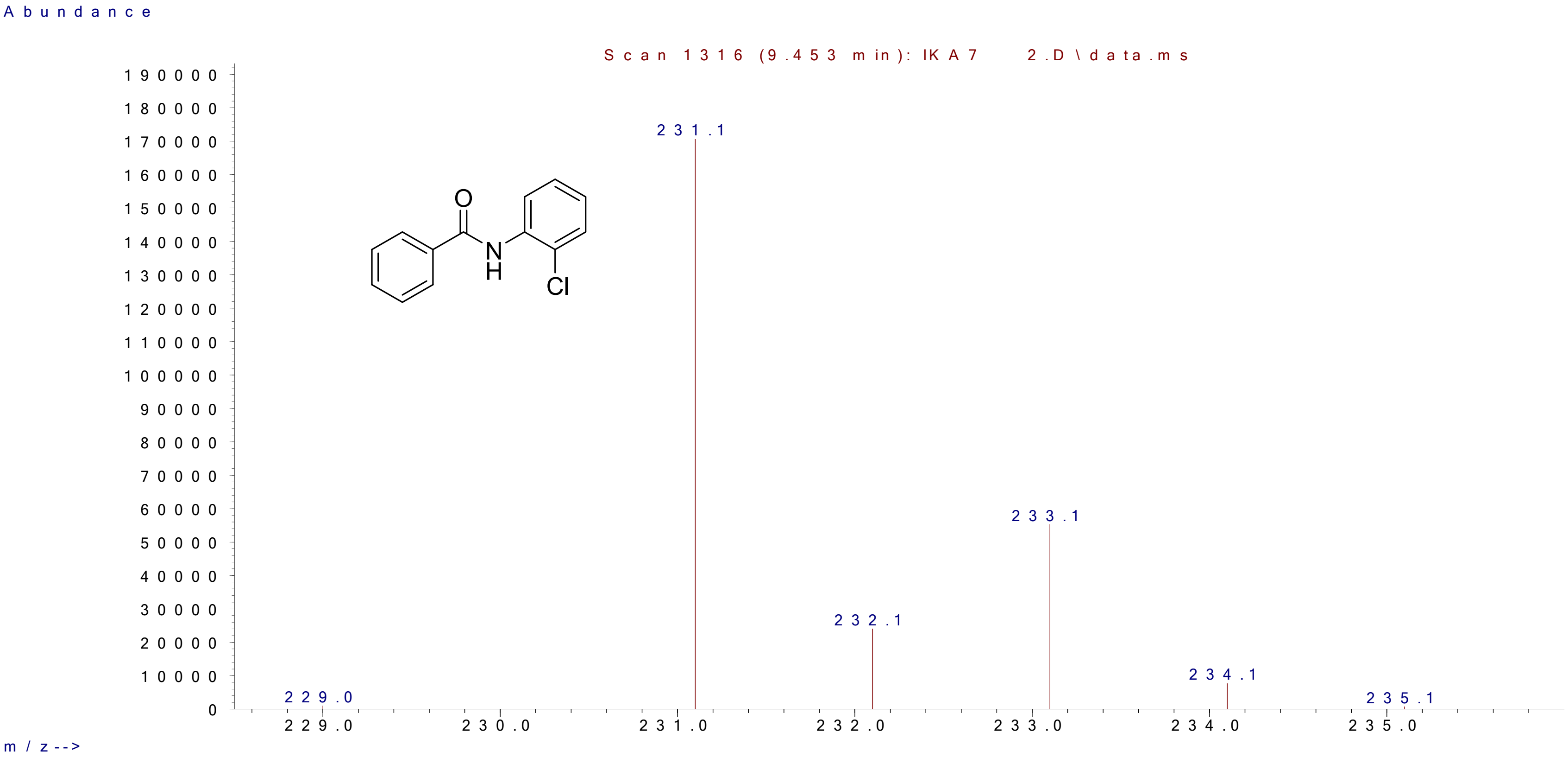
Abundance
Scan 1316 (9.453 min): IKA7 2.D\data.ms
190000
180000
170000
160000
150000
140000
130000
120000
110000
100000
90000
80000
70000
60000
50000
40000
30000
20000
10000
0
231.1
233.1
232.1
234.1
229.0
235.1
O
N
H
Cl
229.0
230.0
231.0
232.0
233.0
234.0
235.0
m/z-->

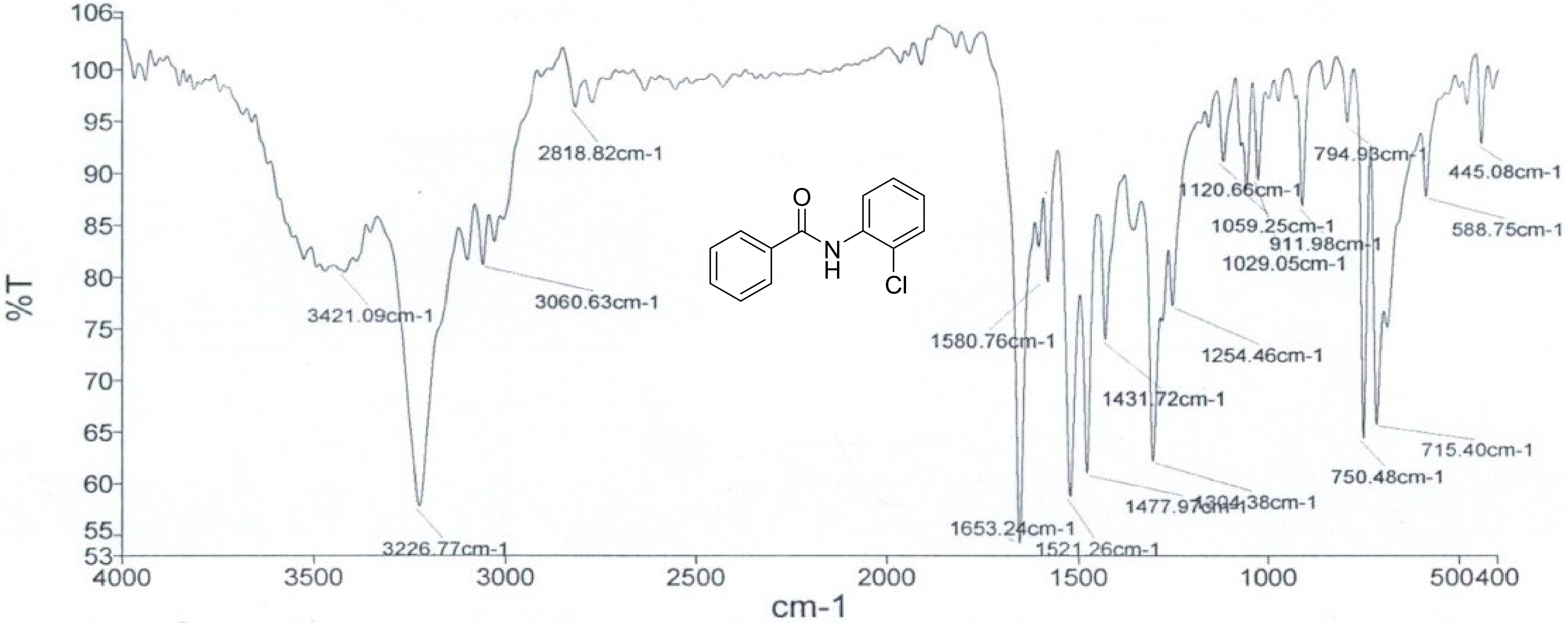

%T
cm-1
2818.82cm-1
3421.09cm-1
3060.63cm-1
3226.77cm-1
1580.76cm-1
1653.24cm-1
1521.26cm-1
1477.97cm-1
1304.38cm-1
1431.72cm-1
1254.46cm-1
1120.66cm-1
1059.25cm-1
1029.05cm-1
911.98cm-1
794.98cm-1
750.48cm-1
715.40cm-1
588.75cm-1
445.08cm-1
O
N
H
Cl

^{1}H NMR, ^{13}C NMR, mass and IR spectrum of *N-(*2-iodophenyl)benzamide (3f)

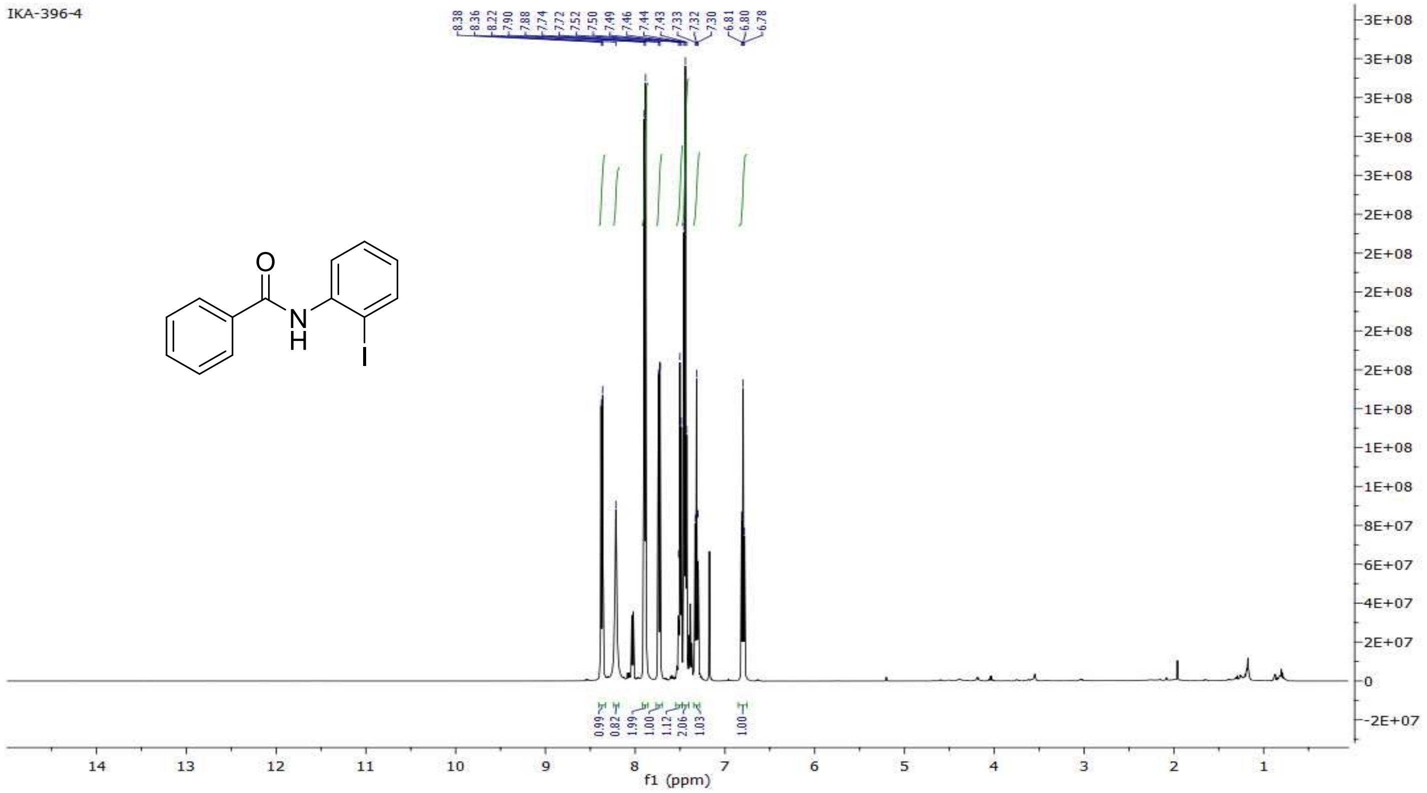

IKA396-4

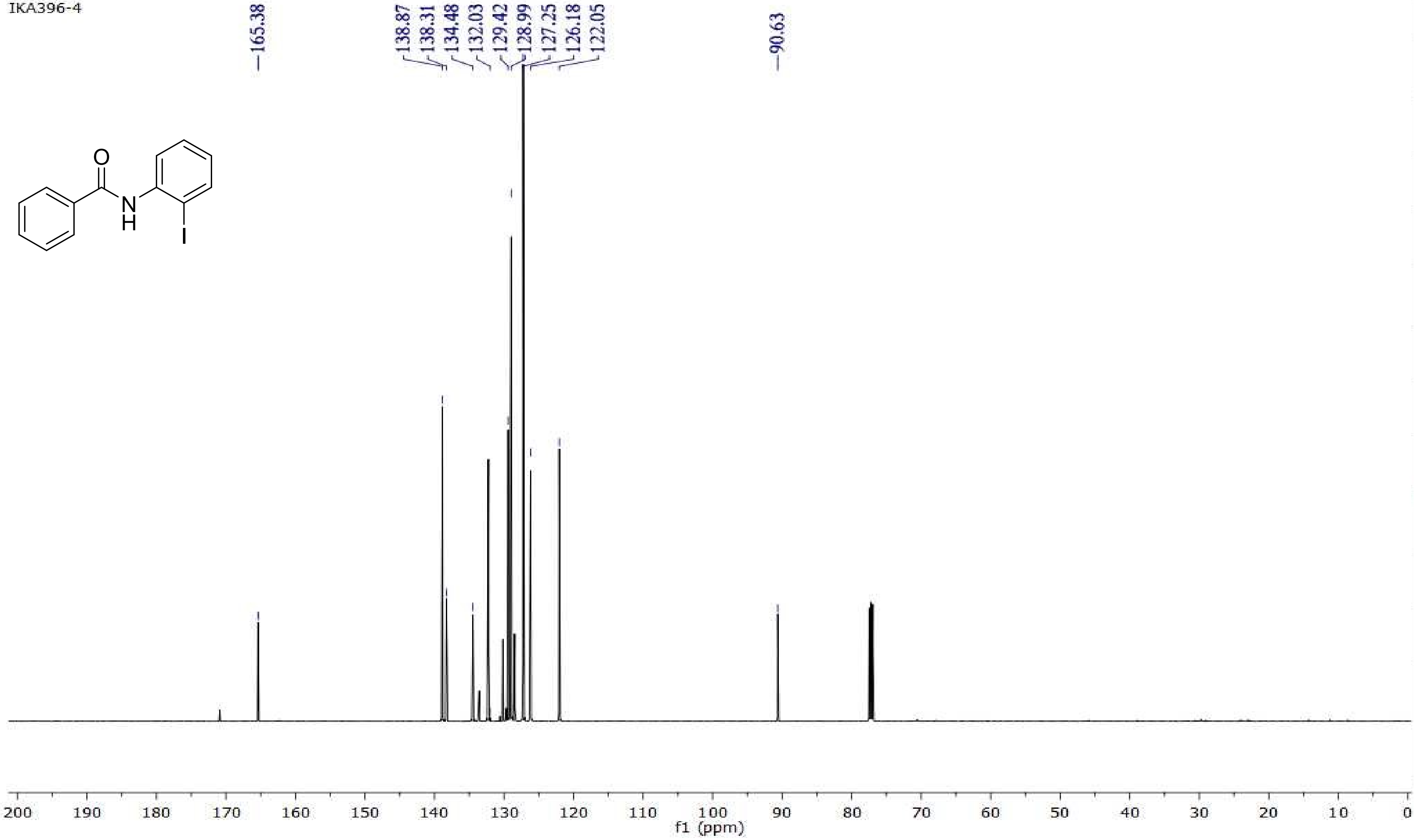

165.38
138.87
138.31
134.48
132.03
129.42
128.99
127.25
126.18
122.05
90.63
O
N
H
I
200 190 180 170 160 150 140 130 120 110 100 90 80 70 60 50 40 30 20 10 0
f1 (ppm)

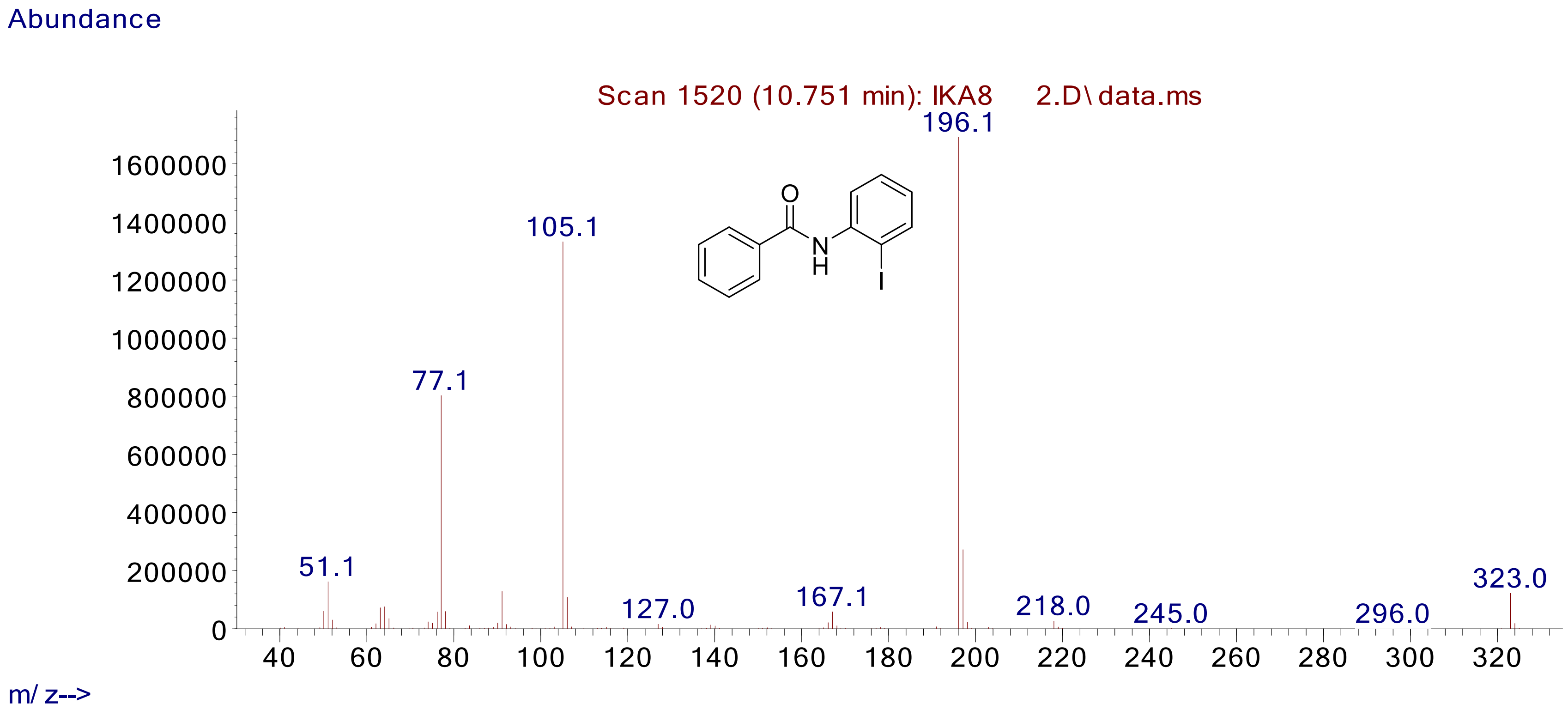
Abundance
Scan 1520 (10.751 min): IKA8   2.D\ data.ms
196.1
105.1
77.1
51.1
127.0
167.1
218.0
245.0
296.0
323.0
1600000
1400000
1200000
1000000
800000
600000
400000
200000
0
40
60
80
100
120
140
160
180
200
220
240
260
280
300
320
m/ z-->
O
N
H
I

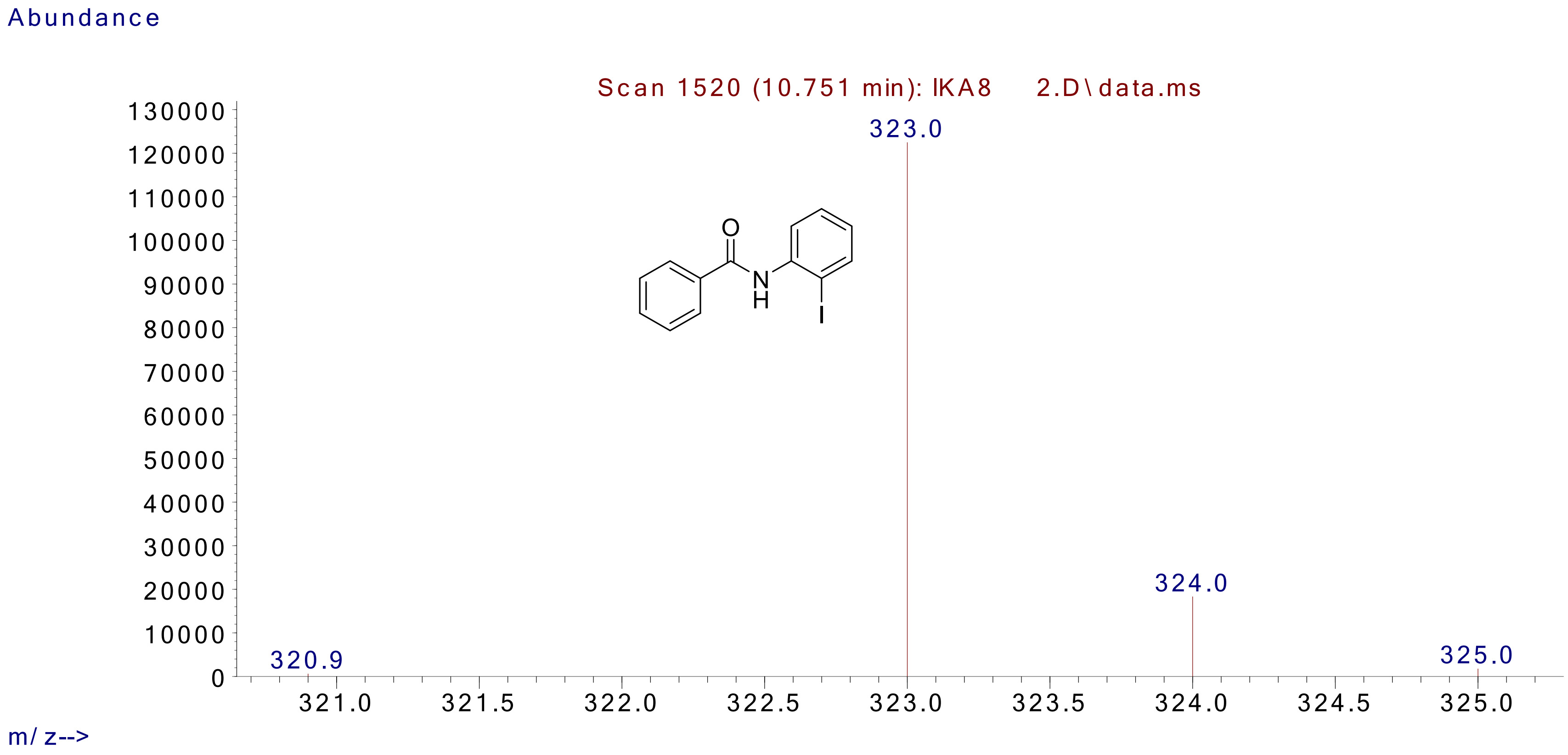

Abundance
Scan 1520 (10.751 min): IKA8 2.D\data.ms
130000
120000
110000
100000
90000
80000
70000
60000
50000
40000
30000
20000
10000
0
323.0
324.0
320.9
325.0
O
N
H
I
321.0
321.5
322.0
322.5
323.0
323.5
324.0
324.5
325.0
m/z-->

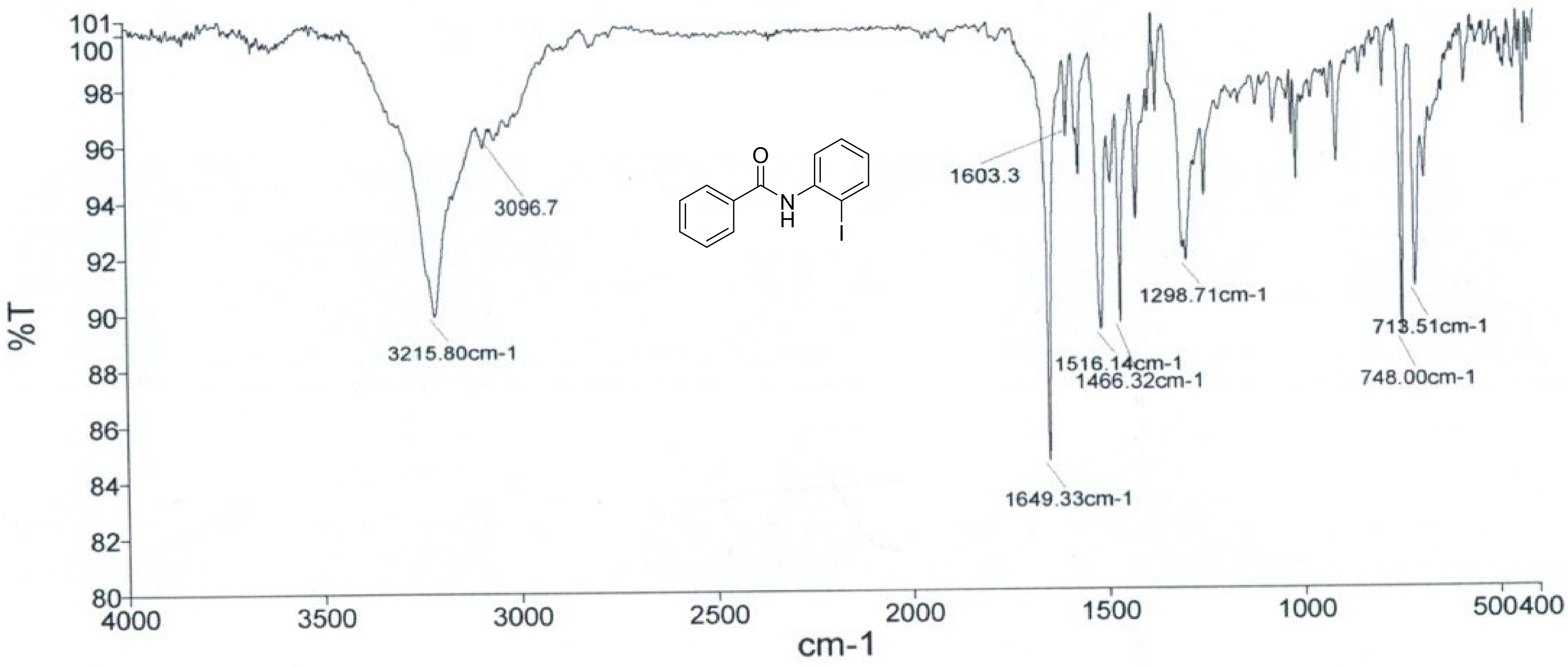
%T
cm-1
3215.80cm-1
3096.7
1603.3
1649.33cm-1
1516.14cm-1
1466.32cm-1
1298.71cm-1
713.51cm-1
748.00cm-1
O
N
H
I

^{1}H NMR, mass and IR spectrum of *N*-(4-chlorophenyl)benzamide (3j)

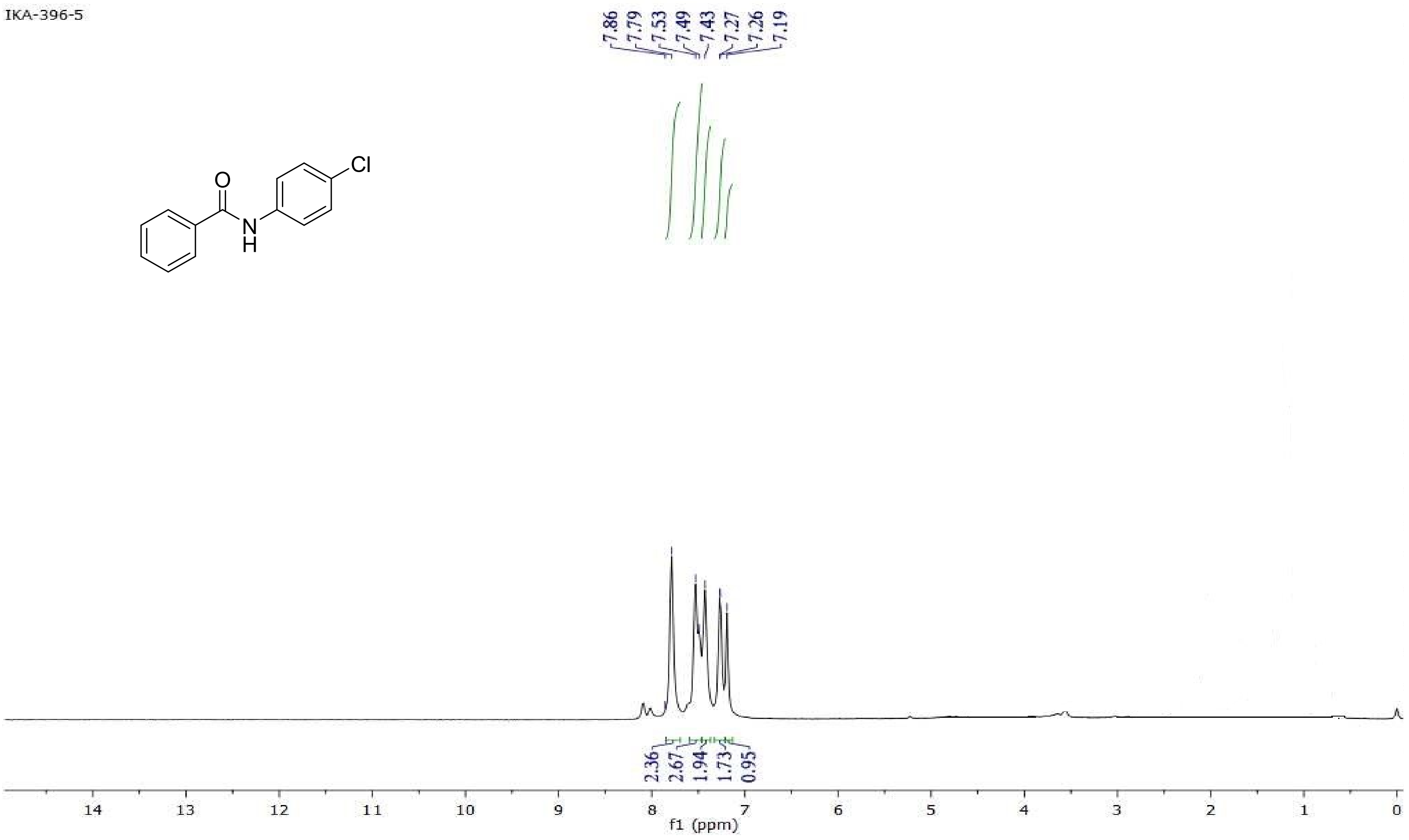

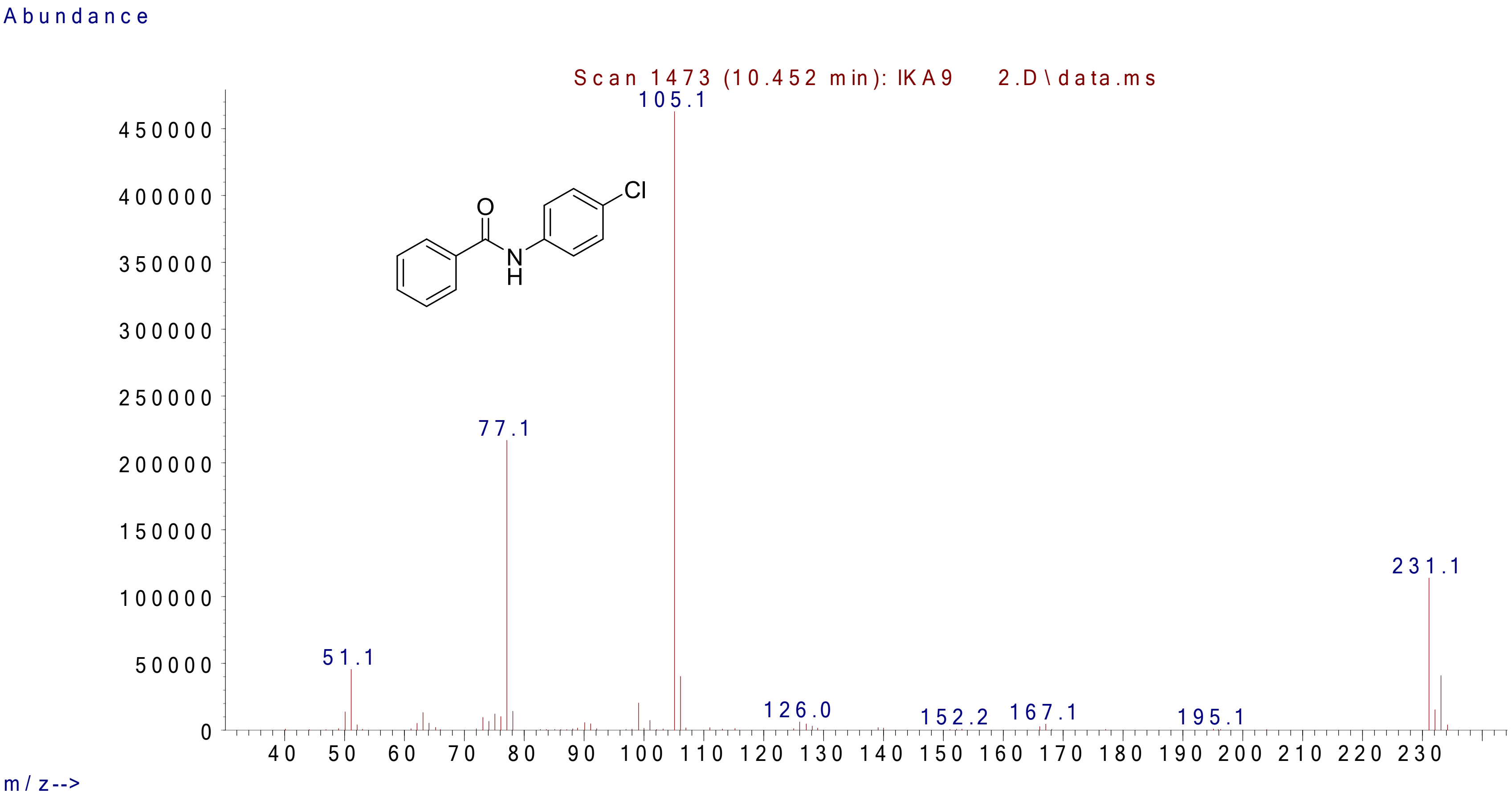
Abundance
Scan 1473 (10.452 min): IKA9 2.D\data.ms
105.1
77.1
231.1
51.1
126.0
152.2
167.1
195.1
450000
400000
350000
300000
250000
200000
150000
100000
50000
0
40 50 60 70 80 90 100 110 120 130 140 150 160 170 180 190 200 210 220 230
m/z-->

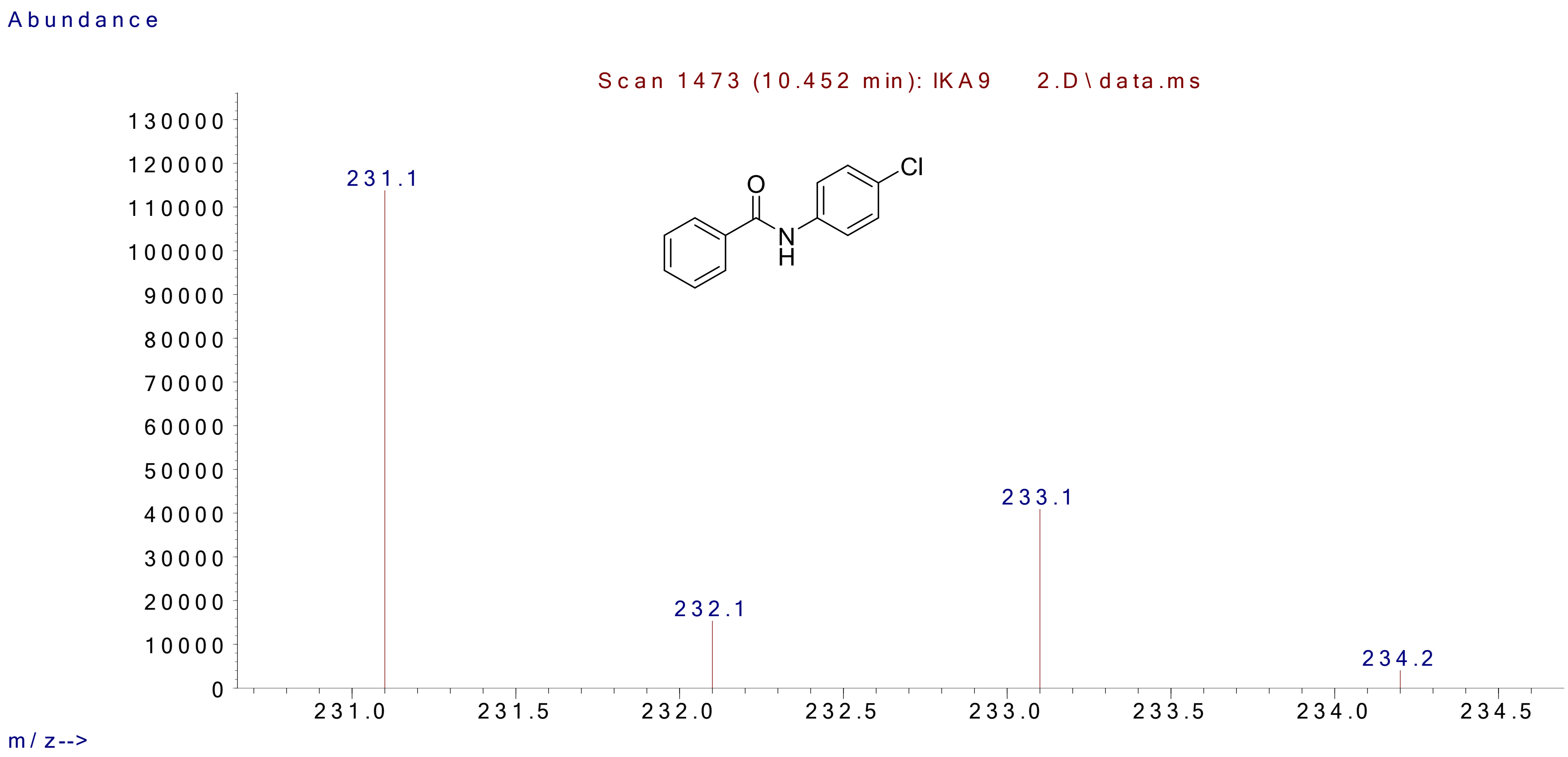
Abundance
Scan 1473 (10.452 min): IKA9 2.D\data.ms
130000
120000
110000
100000
90000
80000
70000
60000
50000
40000
30000
20000
10000
0
231.1
232.1
233.1
234.2
O
N
H
Cl
231.0
231.5
232.0
232.5
233.0
233.5
234.0
234.5
m/z-->

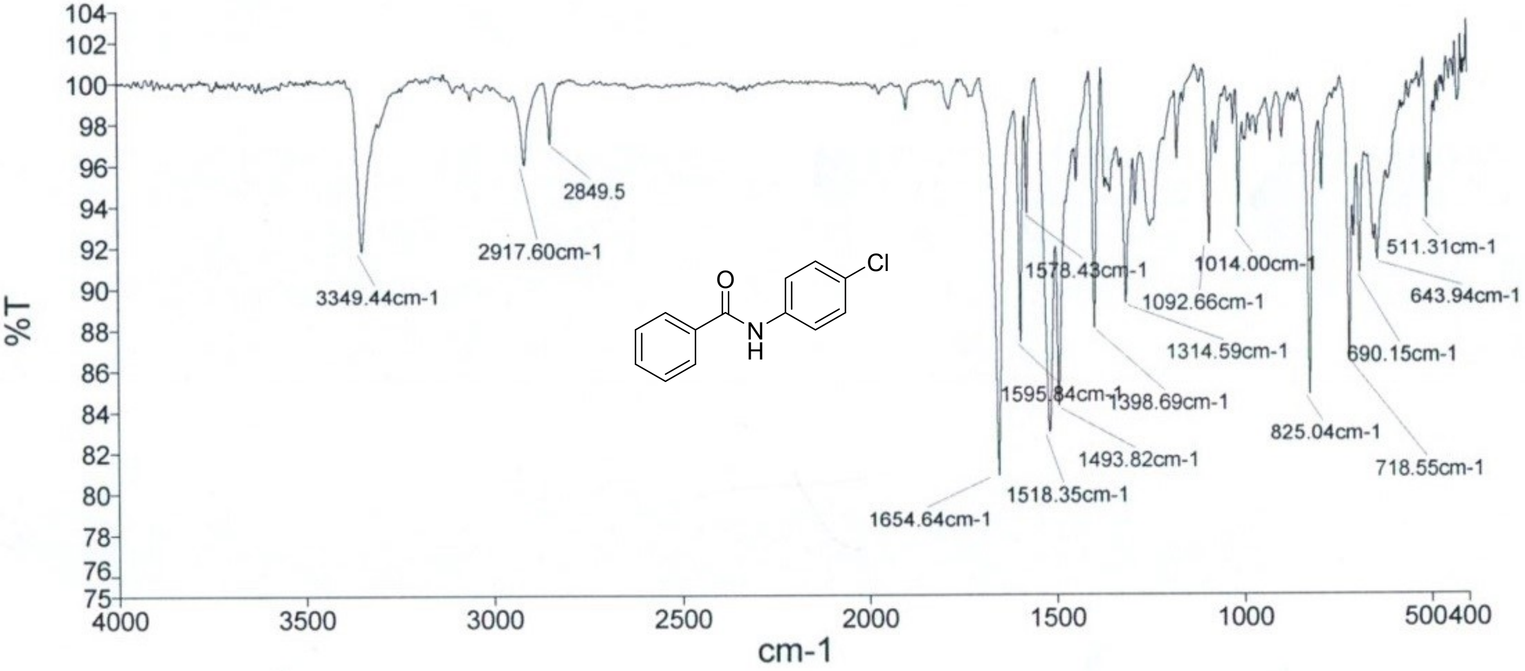

104
102
100
98
96
94
92
90
88
86
84
82
80
78
76
75
%T
4000
3500
3000
2500
2000
1500
1000
500400
cm-1
3349.44cm-1
2917.60cm-1
2849.5
1654.64cm-1
1595.84cm-1
1578.43cm-1
1518.35cm-1
1493.82cm-1
1398.69cm-1
1314.59cm-1
1092.66cm-1
1014.00cm-1
825.04cm-1
718.55cm-1
690.15cm-1
643.94cm-1
511.31cm-1
O
N
H
Cl

^{1}H NMR, ^{13}C NMR, mass and IR spectrum of *N*-(4-bromophenyl)benzamide (3i)

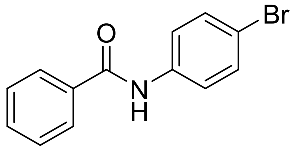

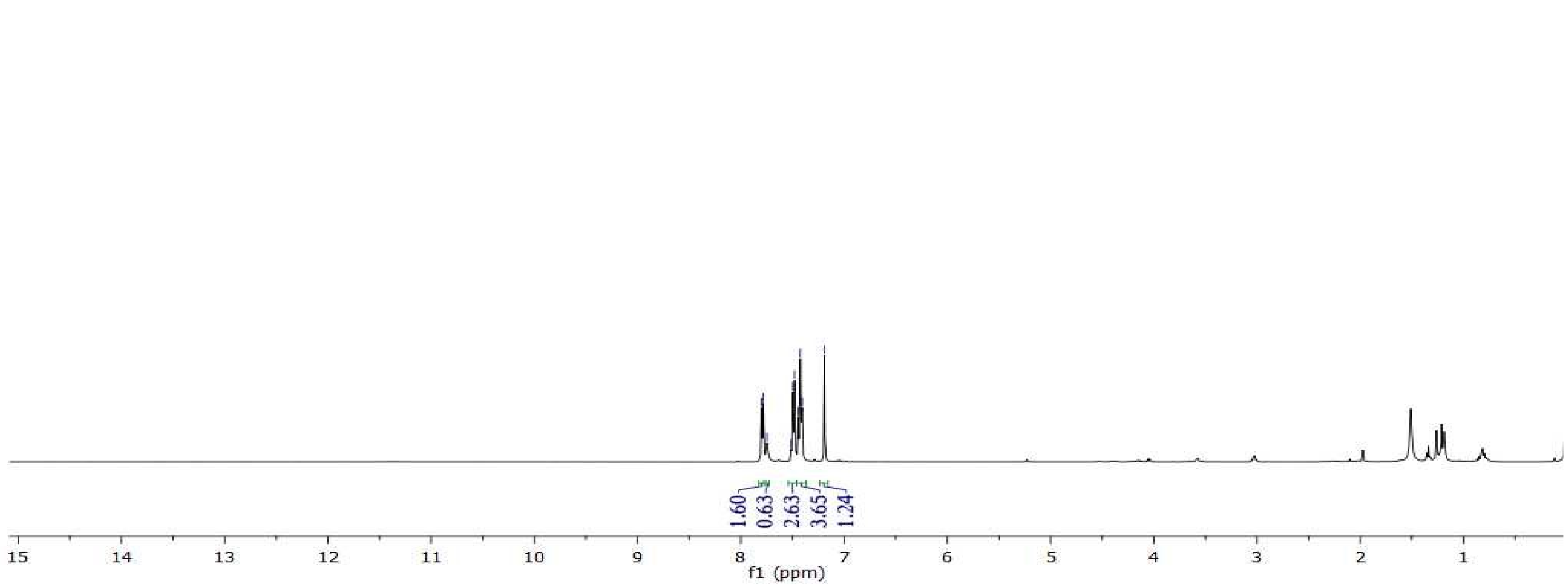

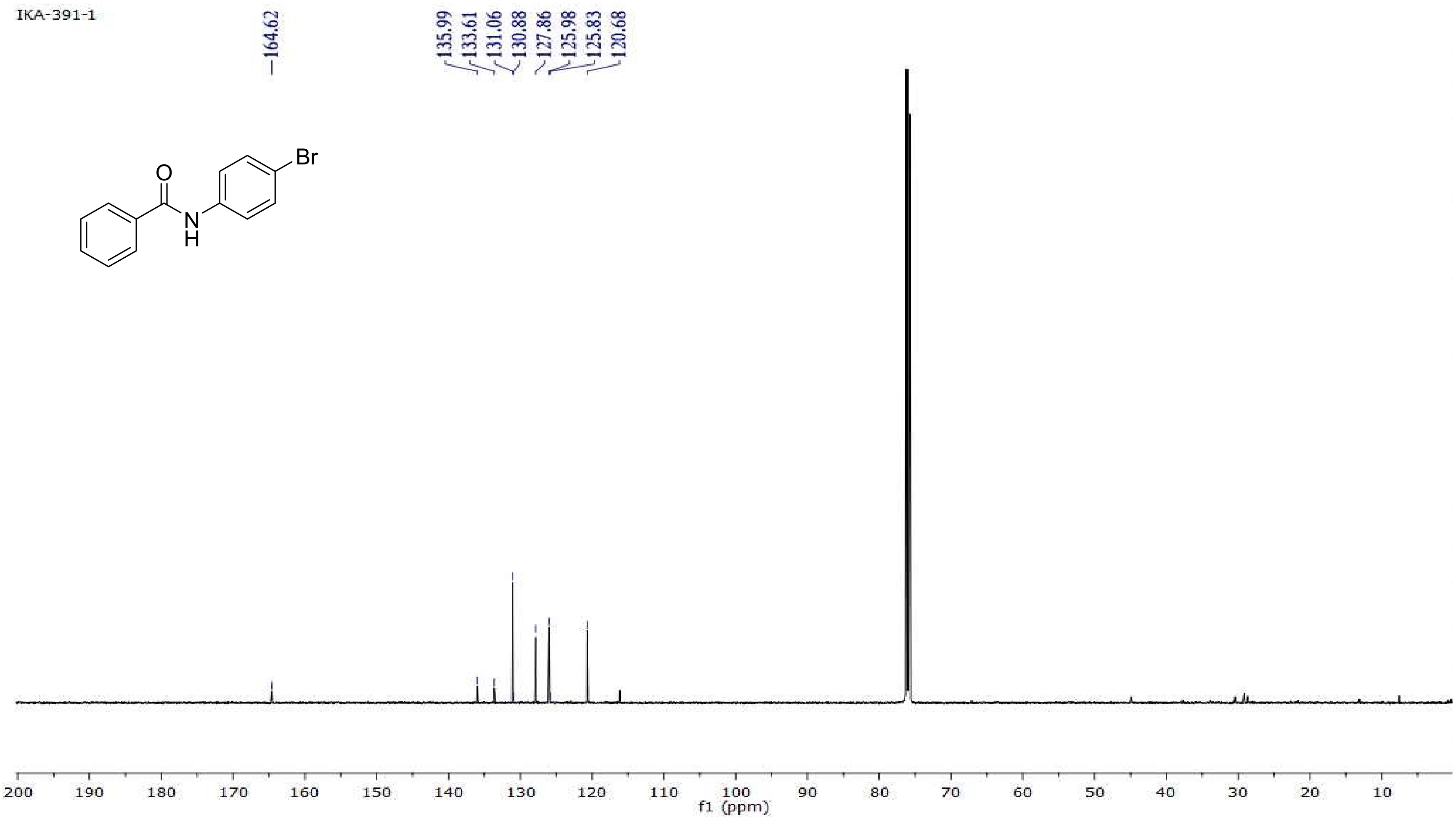
IKA-391-1
164.62
135.99
133.61
131.06
130.88
127.86
125.98
125.83
120.68
Br
O
N
H
200 190 180 170 160 150 140 130 120 110 100 90 80 70 60 50 40 30 20 10
f1 (ppm)

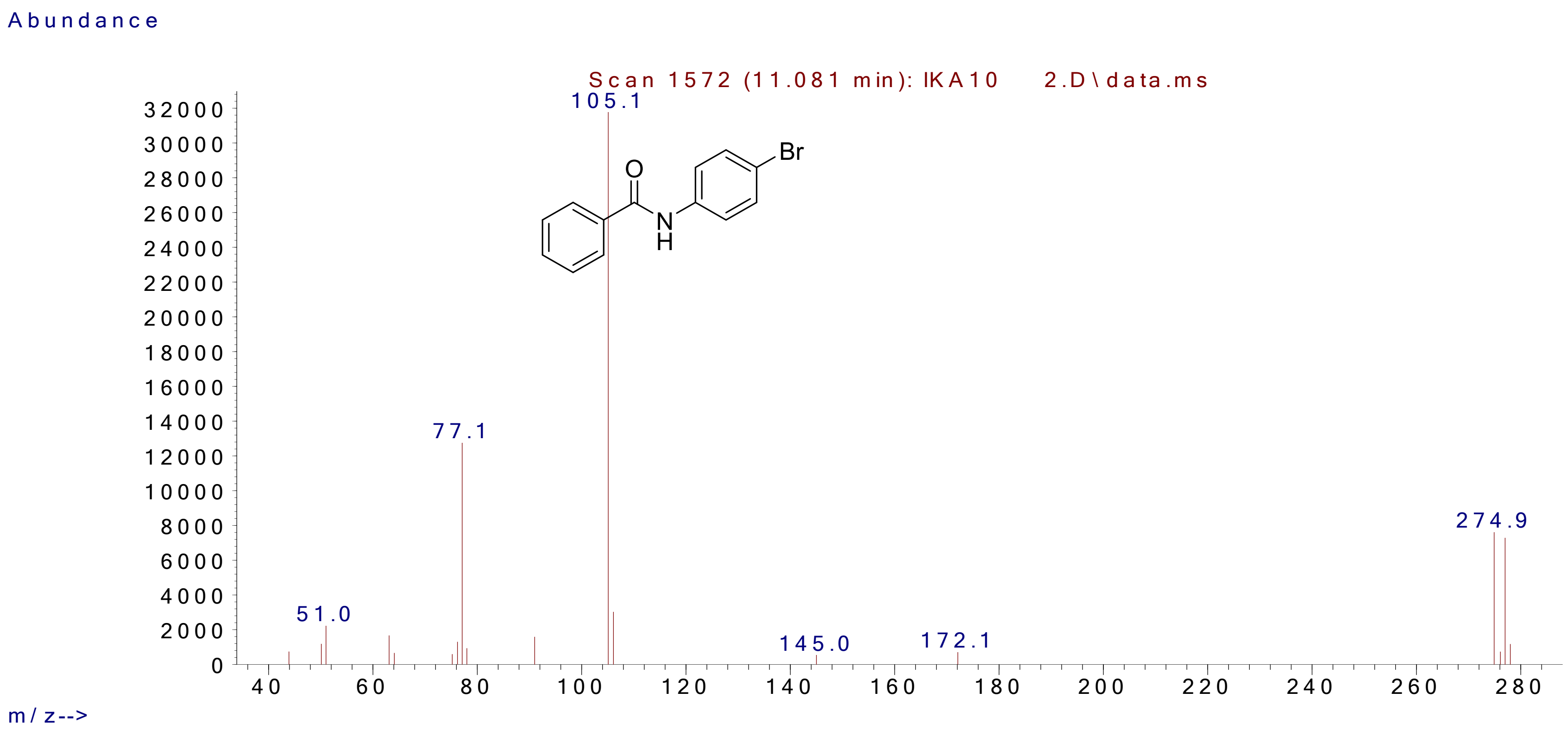
Abundance
Scan 1572 (11.081 min): IKA10 2.D\data.ms
105.1
77.1
274.9
51.0
145.0
172.1
32000
30000
28000
26000
24000
22000
20000
18000
16000
14000
12000
10000
8000
6000
4000
2000
0
40
60
80
100
120
140
160
180
200
220
240
260
280
m/z-->
O
N
H
Br

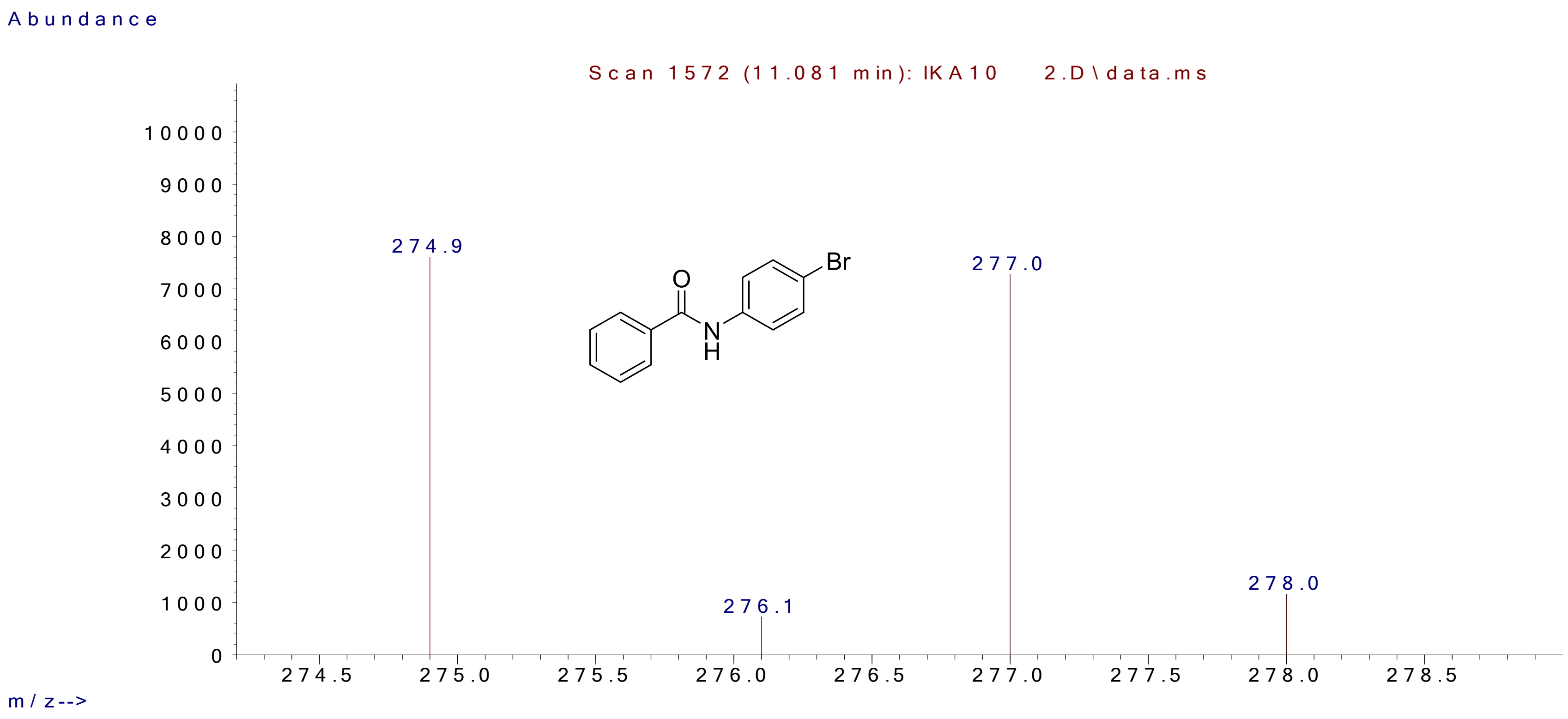
Abundance
Scan 1572 (11.081 min): IKA10 2.D\data.ms
10000
9000
8000
7000
6000
5000
4000
3000
2000
1000
0
274.9
277.0
276.1
278.0
O
N
H
Br
274.5
275.0
275.5
276.0
276.5
277.0
277.5
278.0
278.5
m/z-->

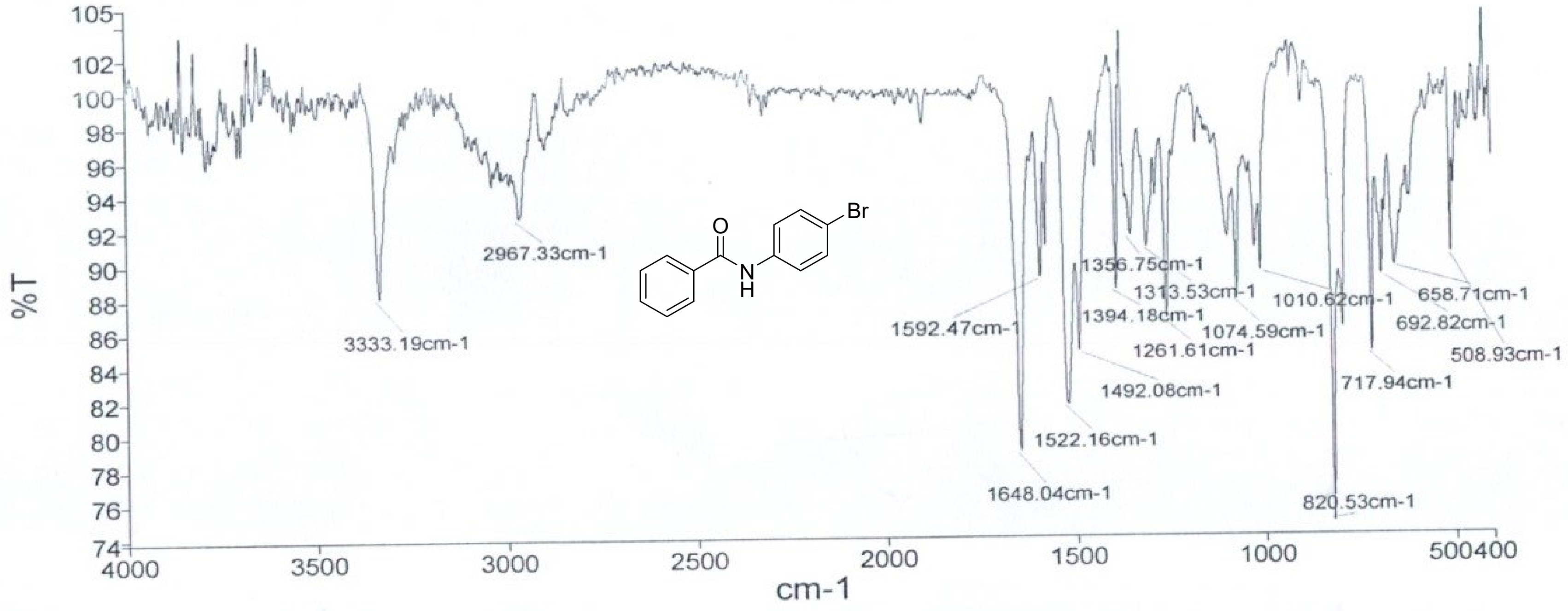
%T
cm-1
Br
O
N
H
3333.19cm-1
2967.33cm-1
1648.04cm-1
1592.47cm-1
1522.16cm-1
1492.08cm-1
1394.18cm-1
1356.75cm-1
1313.53cm-1
1261.61cm-1
1074.59cm-1
1010.62cm-1
820.53cm-1
717.94cm-1
692.82cm-1
658.71cm-1
508.93cm-1

^{1}H NMR, ^{13}C NMR, mass and IR spectrum of *N*-(2,6-dimethylphenyl)benzamide (3n)

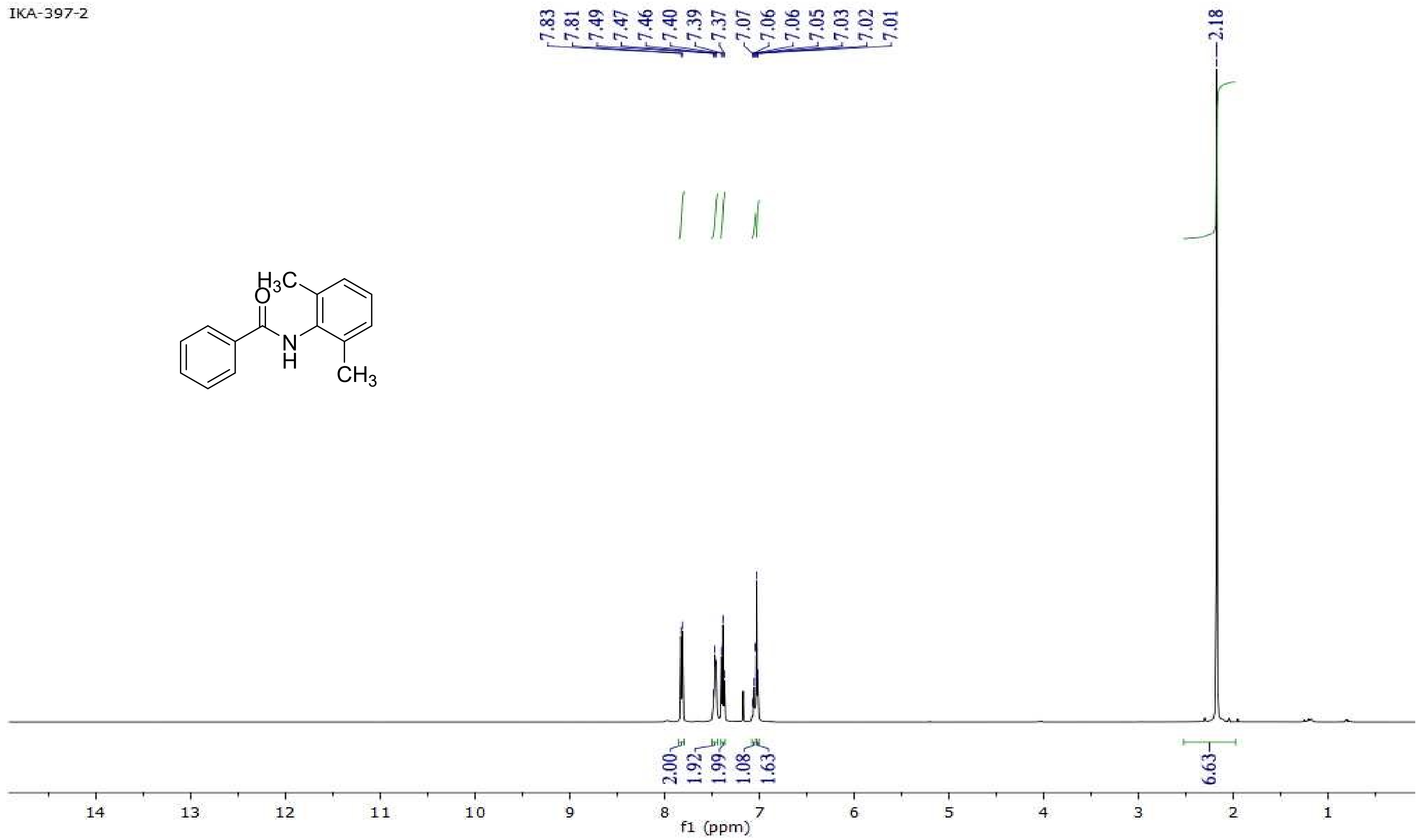

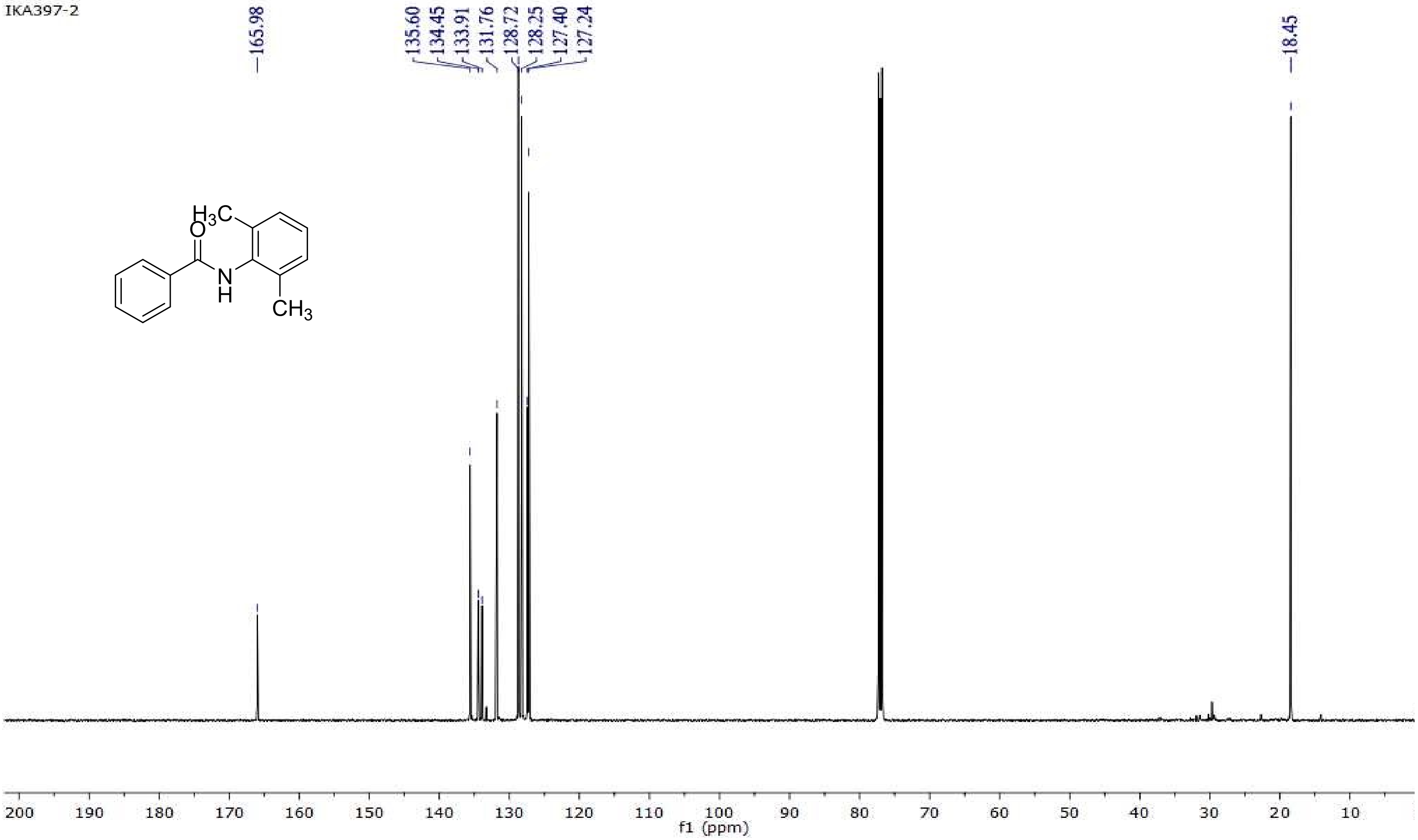

IKA397-2
165.98
135.60
134.45
133.91
131.76
128.72
128.25
127.40
127.24
18.45
H_3C
O
N
H
CH_3
200 190 180 170 160 150 140 130 120 110 100 90 80 70 60 50 40 30 20 10
f1 (ppm)

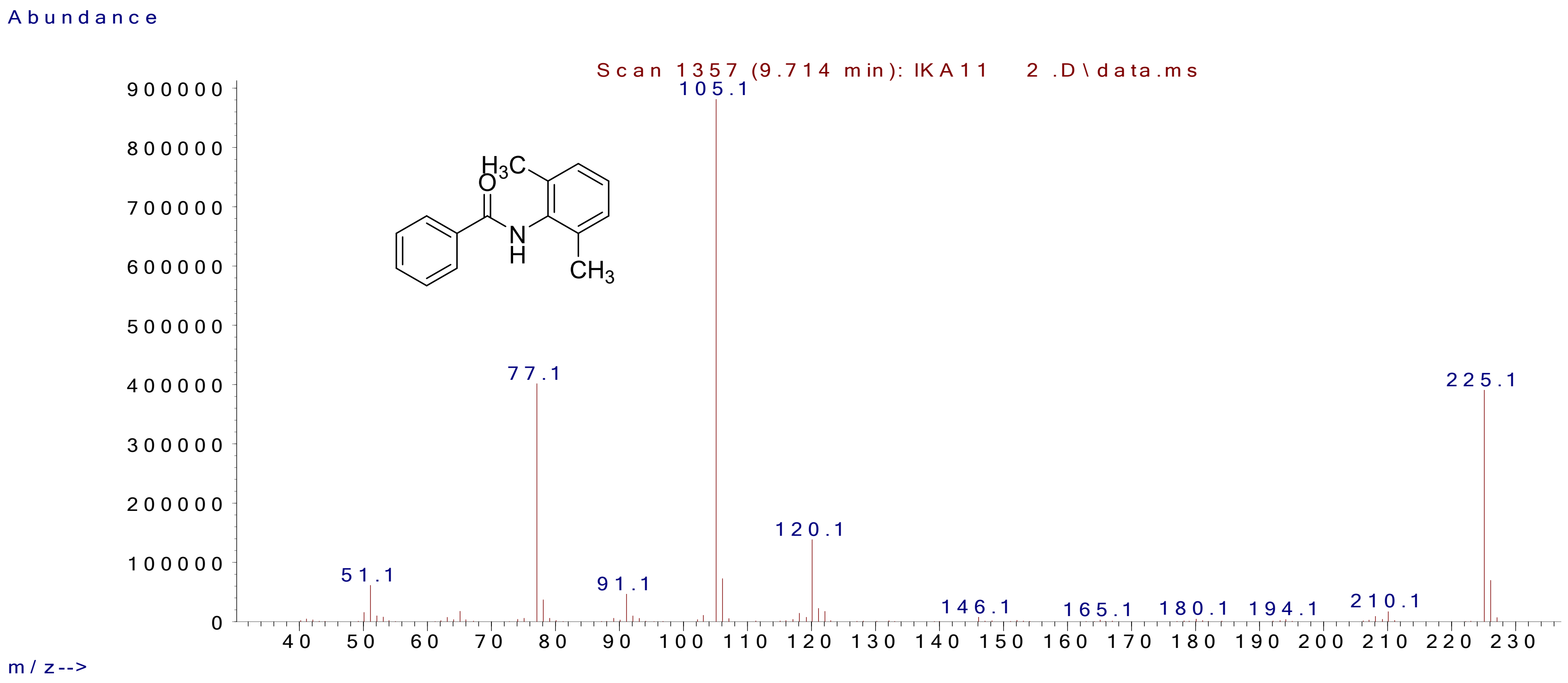

Abundance
Scan 1357 (9.714 min): IKA11 2 .D\data.ms
900000
800000
700000
600000
500000
400000
300000
200000
100000
0
105.1
77.1
225.1
120.1
51.1
91.1
146.1
165.1
180.1
194.1
210.1
40 50 60 70 80 90 100 110 120 130 140 150 160 170 180 190 200 210 220 230
m/z-->

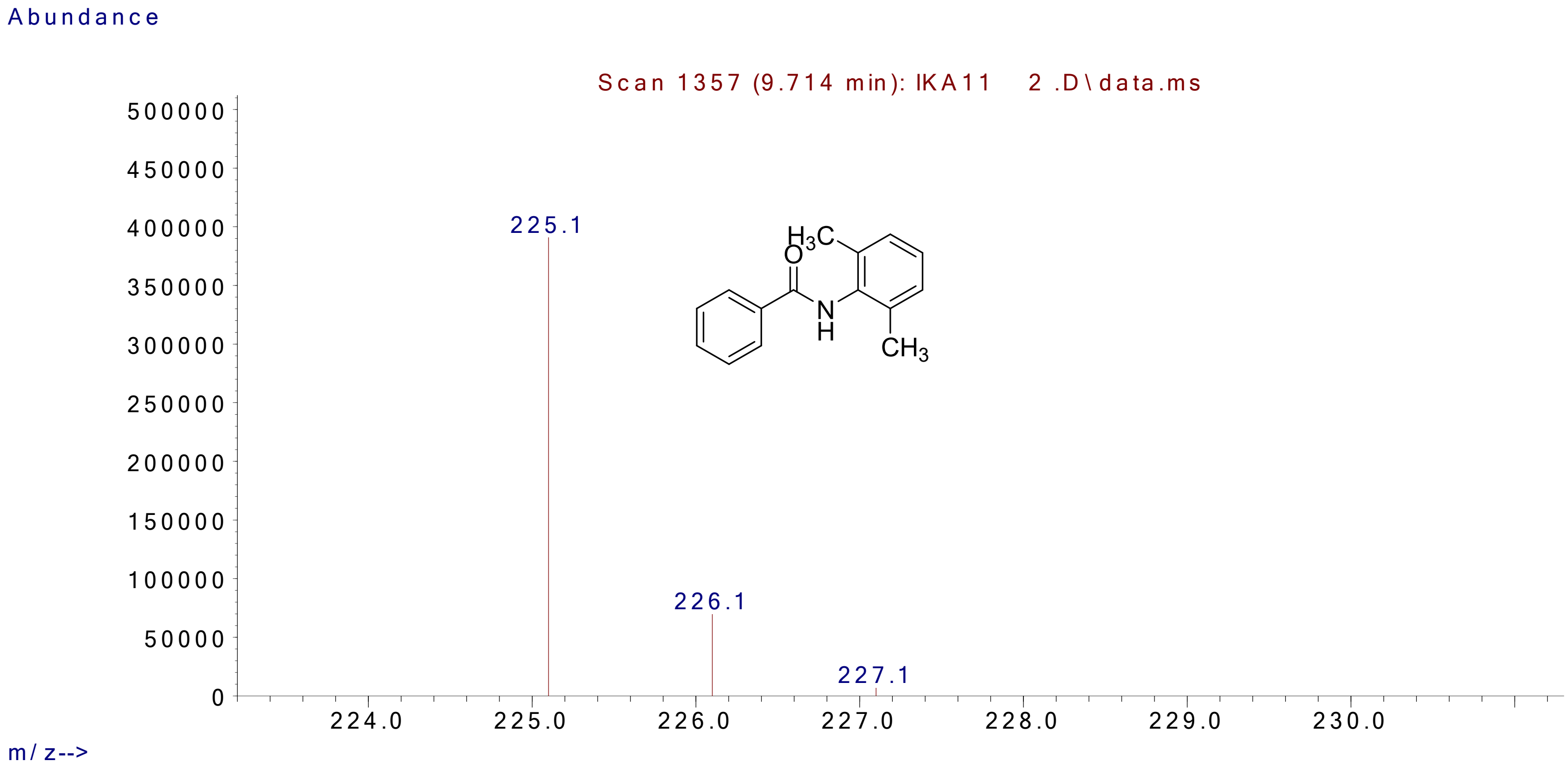
Abundance
Scan 1357 (9.714 min): IKA11 2 .D\data.ms
500000
450000
400000
350000
300000
250000
200000
150000
100000
50000
0
225.1
226.1
227.1
224.0
225.0
226.0
227.0
228.0
229.0
230.0
m/z-->
H3C
O
N
H
CH3

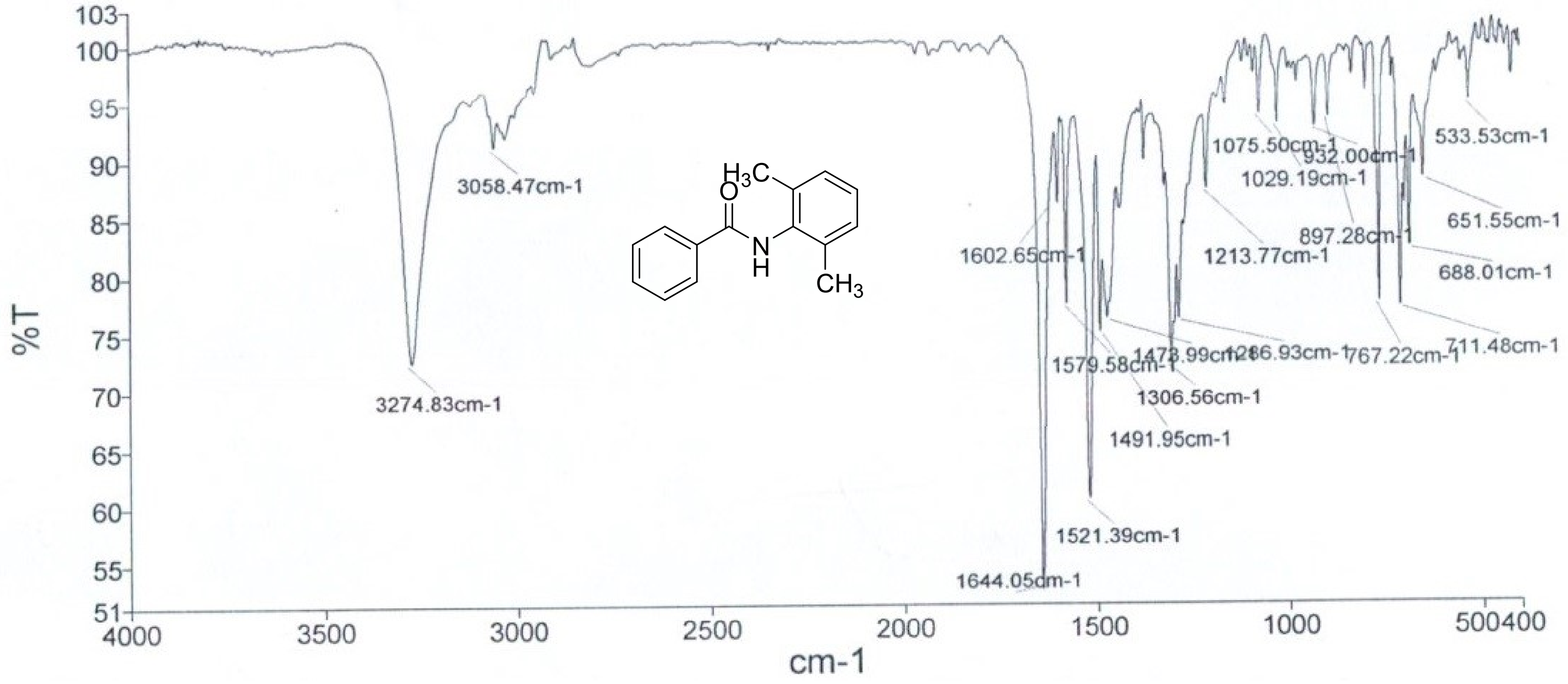

%T
cm-1
3274.83cm-1
3058.47cm-1
1644.05cm-1
1602.65cm-1
1579.58cm-1
1521.39cm-1
1491.95cm-1
1473.99cm-1
1306.56cm-1
1286.93cm-1
1213.77cm-1
1075.50cm-1
1029.19cm-1
932.00cm-1
897.28cm-1
767.22cm-1
711.48cm-1
688.01cm-1
651.55cm-1
533.53cm-1

^{1}H NMR, ^{13}C NMR, mass and IR spectrum of *N*-(4-chloro-3-nitrophenyl)benzamide (3m)

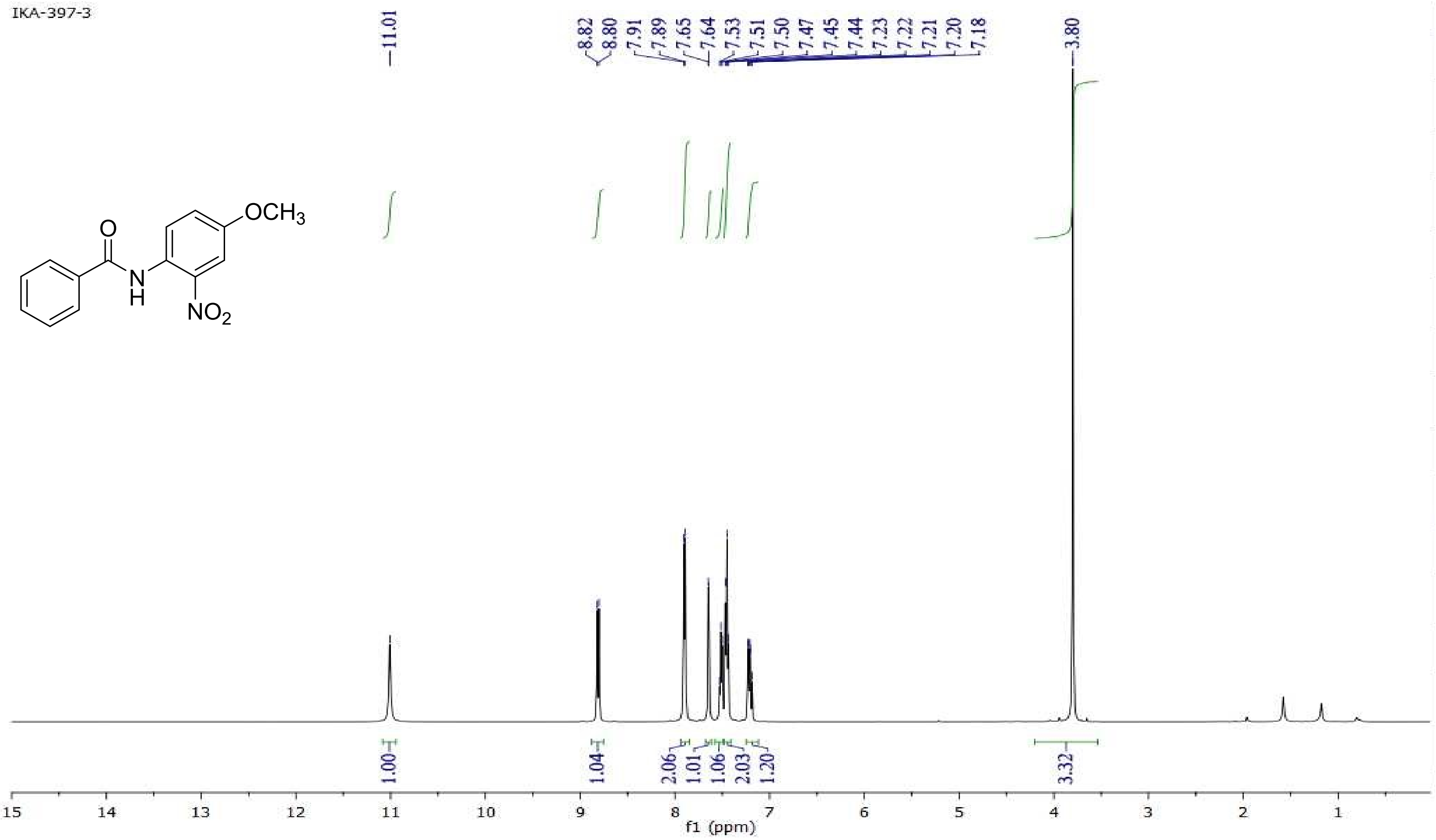

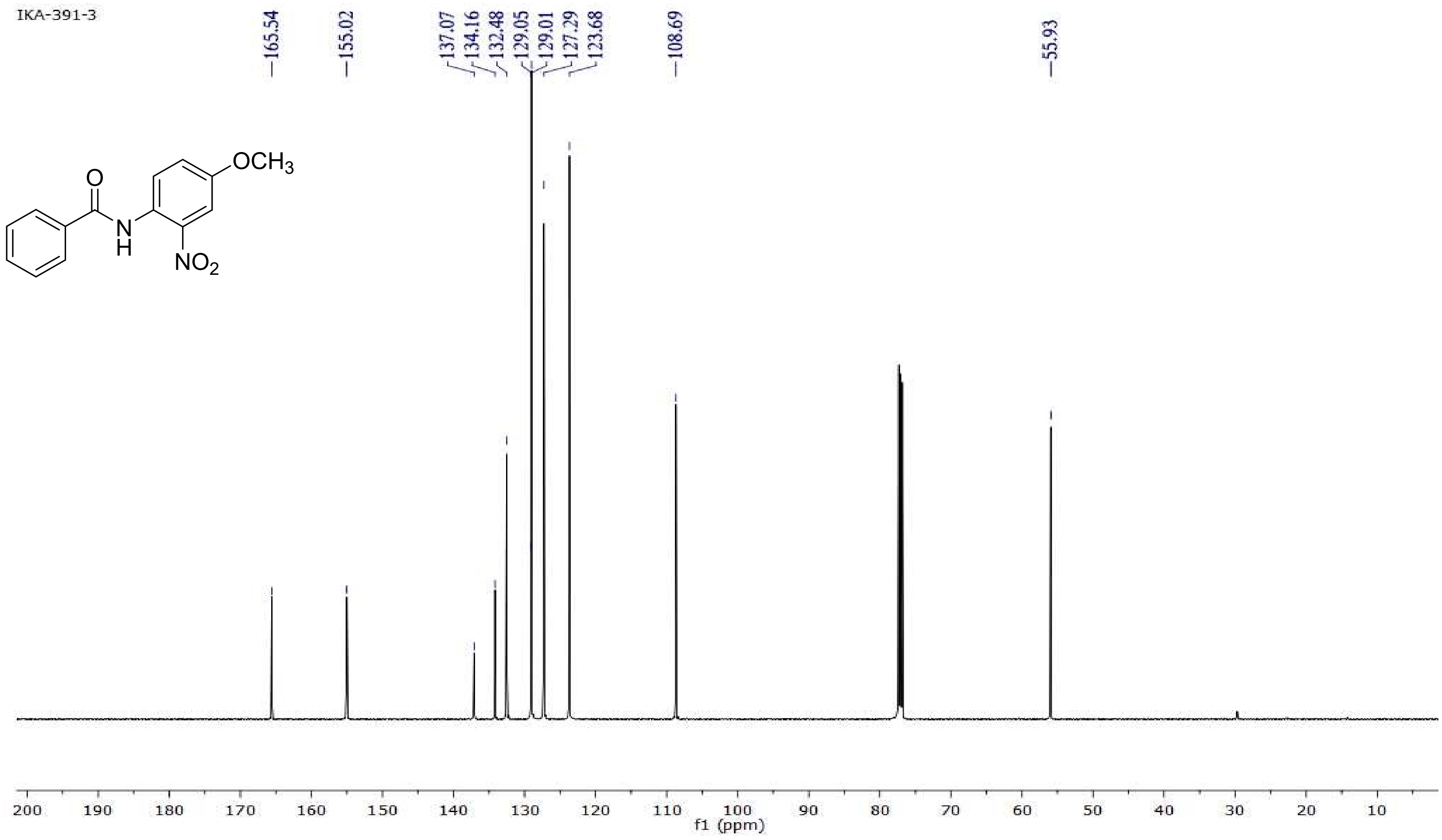
IKA-391-3
165.54
155.02
137.07
134.16
132.48
129.05
129.01
127.29
123.68
108.69
55.93
OCH3
O
N
H
NO2
200
190
180
170
160
150
140
130
120
110
100
90
80
70
60
50
40
30
20
10
f1 (ppm)

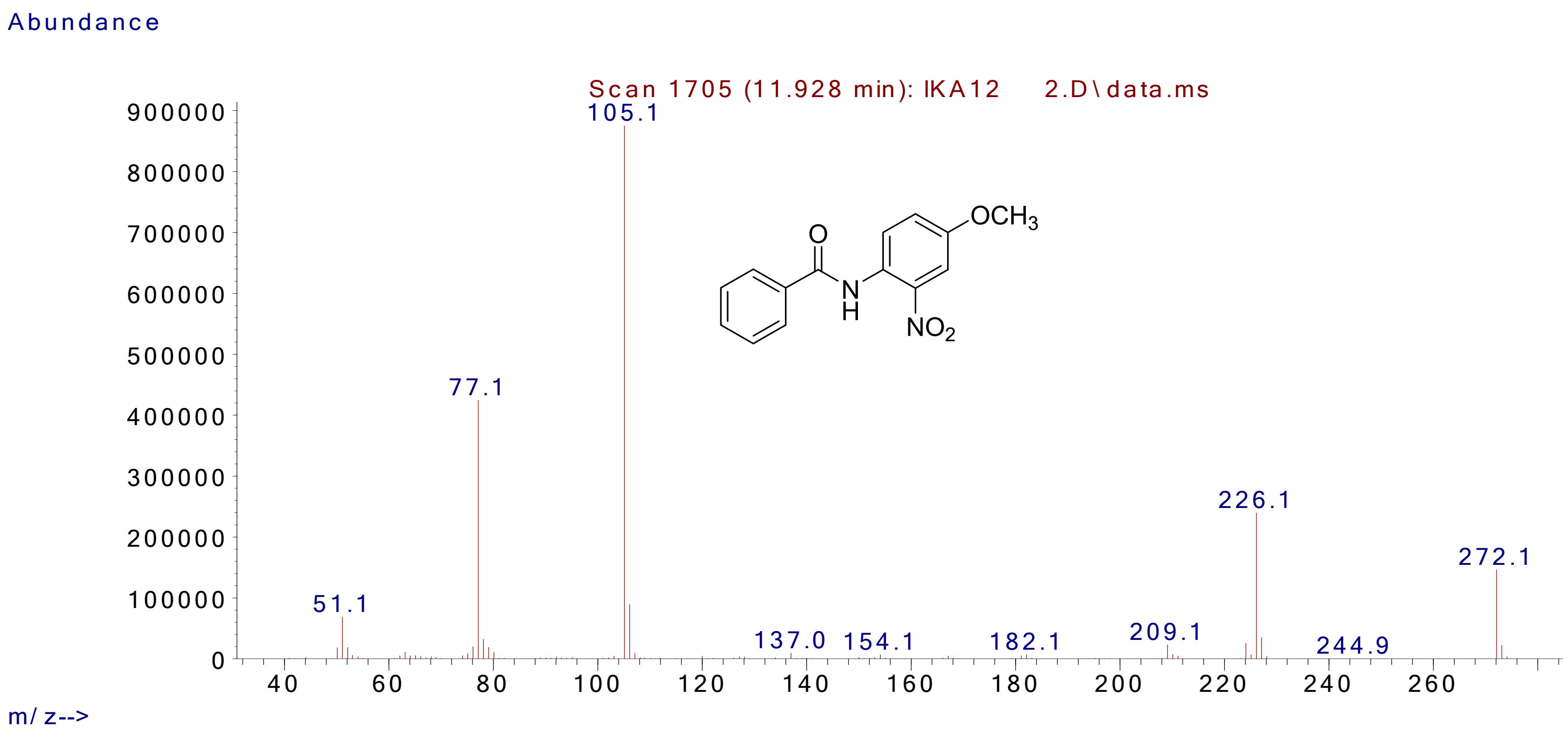
Abundance
Scan 1705 (11.928 min): IKA12 2.D\data.ms
900000
800000
700000
600000
500000
400000
300000
200000
100000
0
105.1
77.1
51.1
137.0
154.1
182.1
209.1
226.1
244.9
272.1
O
N
H
OCH3
NO2
40
60
80
100
120
140
160
180
200
220
240
260
m/z-->

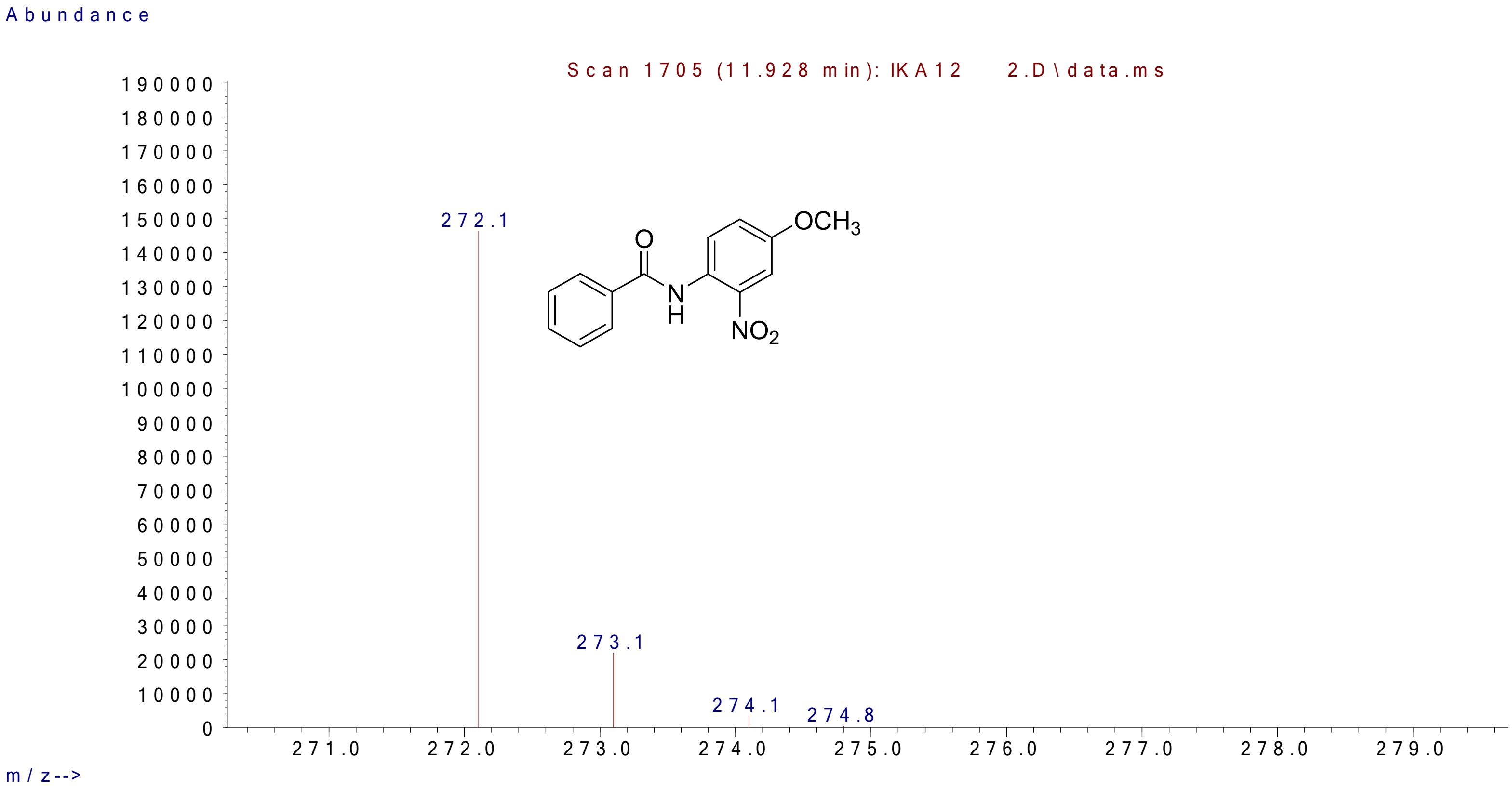
Abundance
Scan 1705 (11.928 min): IKA12 2.D\data.ms
190000
180000
170000
160000
150000
140000
130000
120000
110000
100000
90000
80000
70000
60000
50000
40000
30000
20000
10000
0
272.1
273.1
274.1
274.8
271.0
272.0
273.0
274.0
275.0
276.0
277.0
278.0
279.0
m/z-->
O
N
H
OCH3
NO2

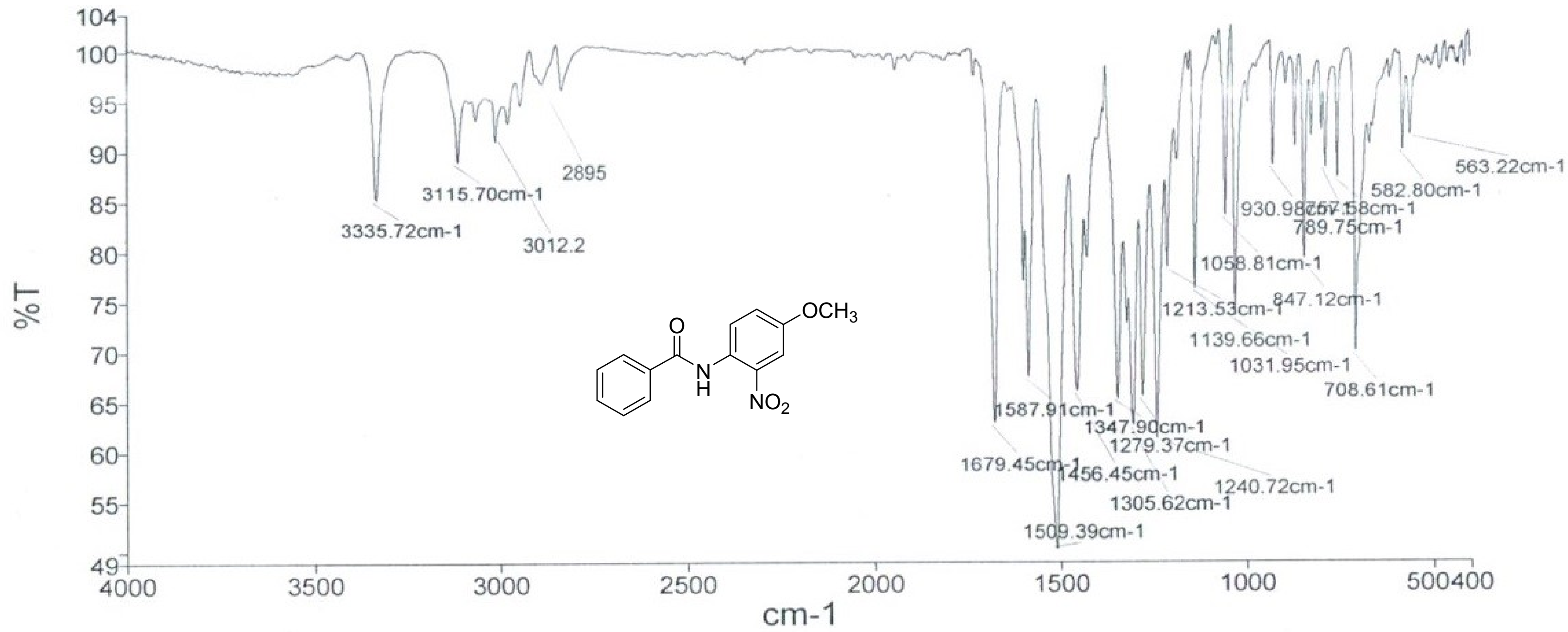
%T
cm-1
3335.72cm-1
3115.70cm-1
3012.2
2895
1679.45cm-1
1587.91cm-1
1509.39cm-1
1456.45cm-1
1347.90cm-1
1305.62cm-1
1279.37cm-1
1240.72cm-1
1213.53cm-1
1139.66cm-1
1058.81cm-1
1031.95cm-1
930.98cm-1
847.12cm-1
789.75cm-1
708.61cm-1
582.80cm-1
563.22cm-1
OCH3
NO2
NH
O

^{1}H NMR, ^{13}C NMR, mass and IR spectrum of *N*-(2-methyl-4-nitrophenyl)benzamide (3l)

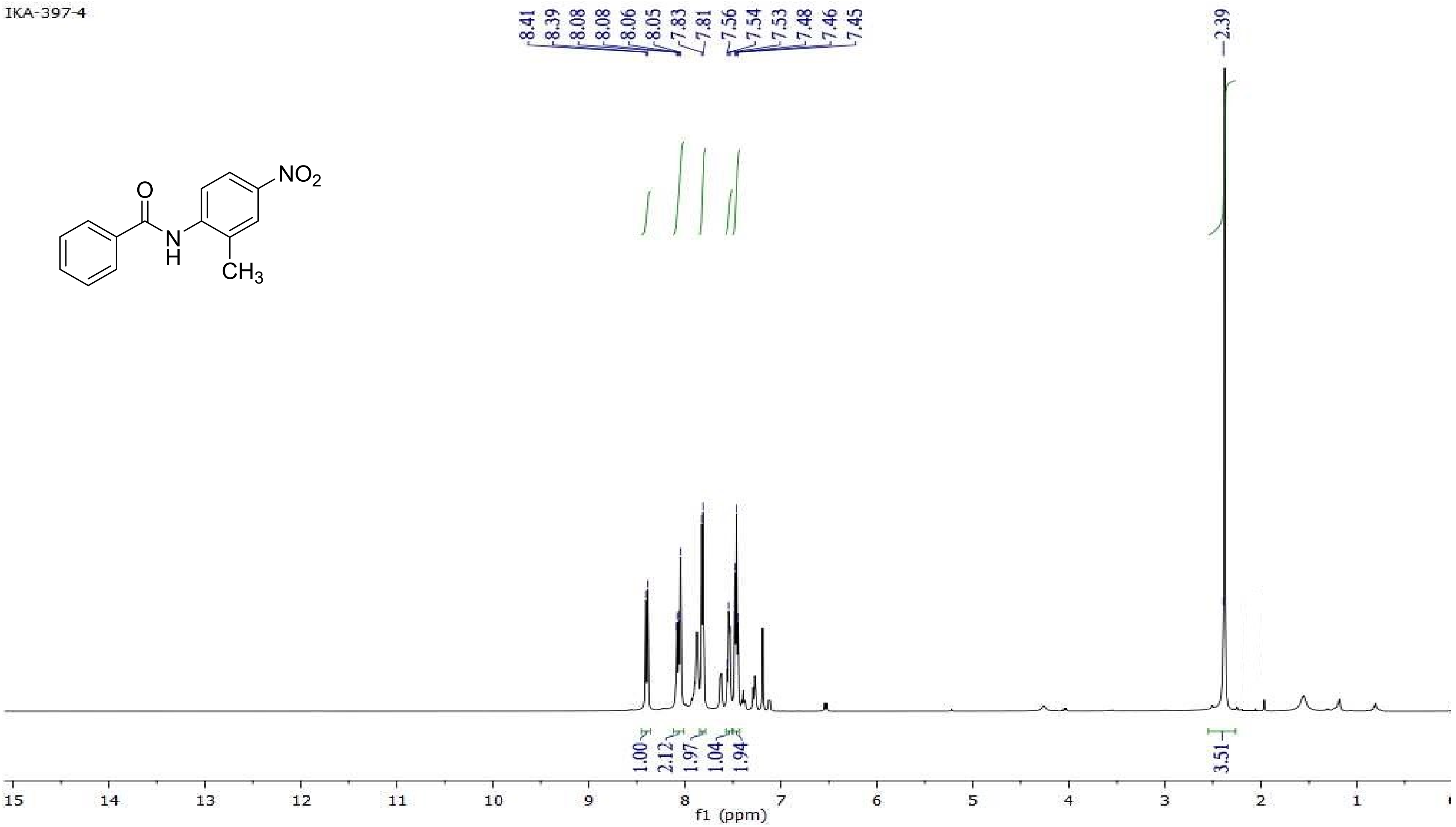

IKA-391-4

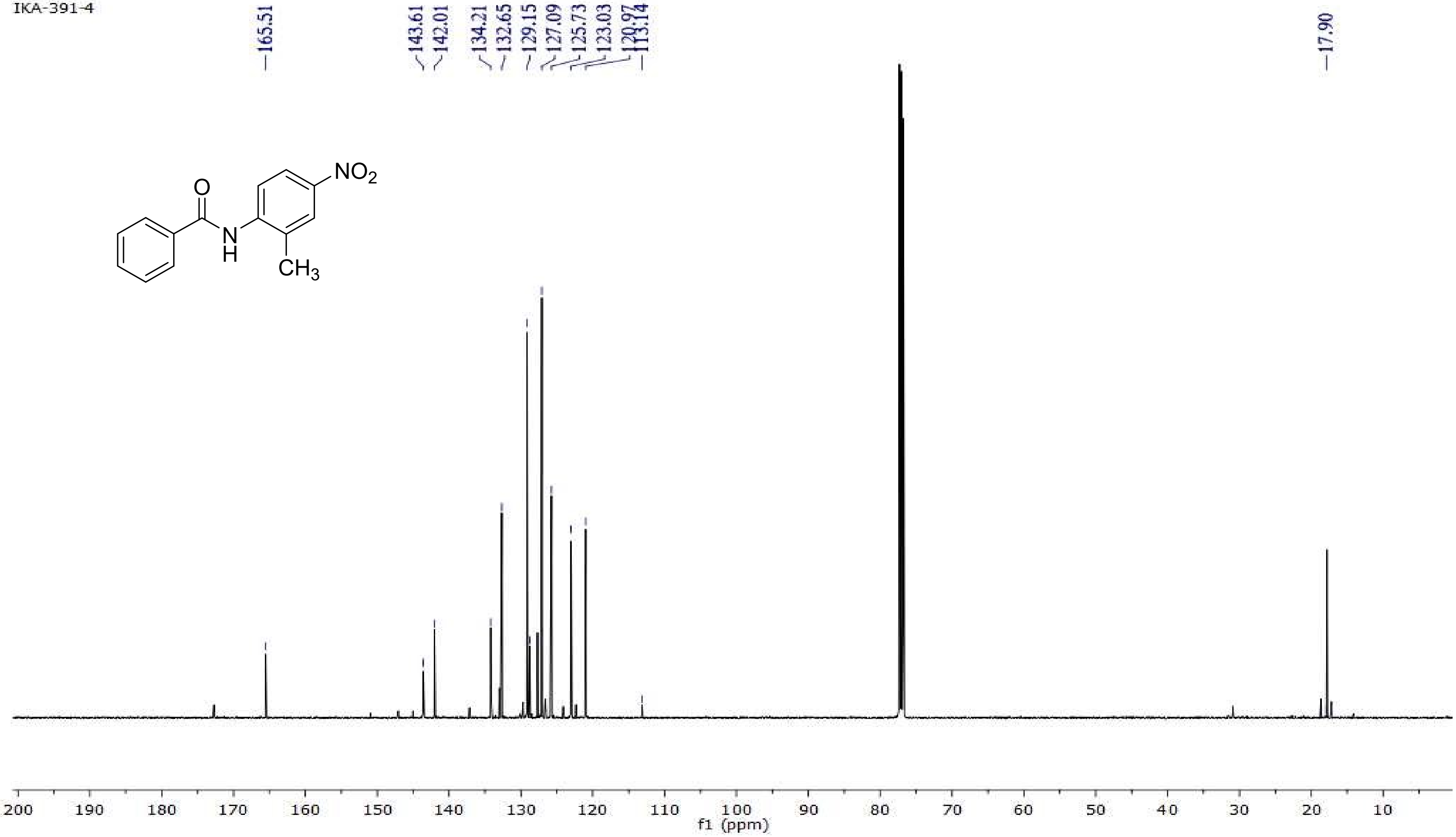

165.51
143.61
142.01
134.21
132.65
129.15
127.09
125.73
123.03
120.97
113.14
17.90
O
N
H
NO2
CH3
200 190 180 170 160 150 140 130 120 110 100 90 80 70 60 50 40 30 20 10
f1 (ppm)

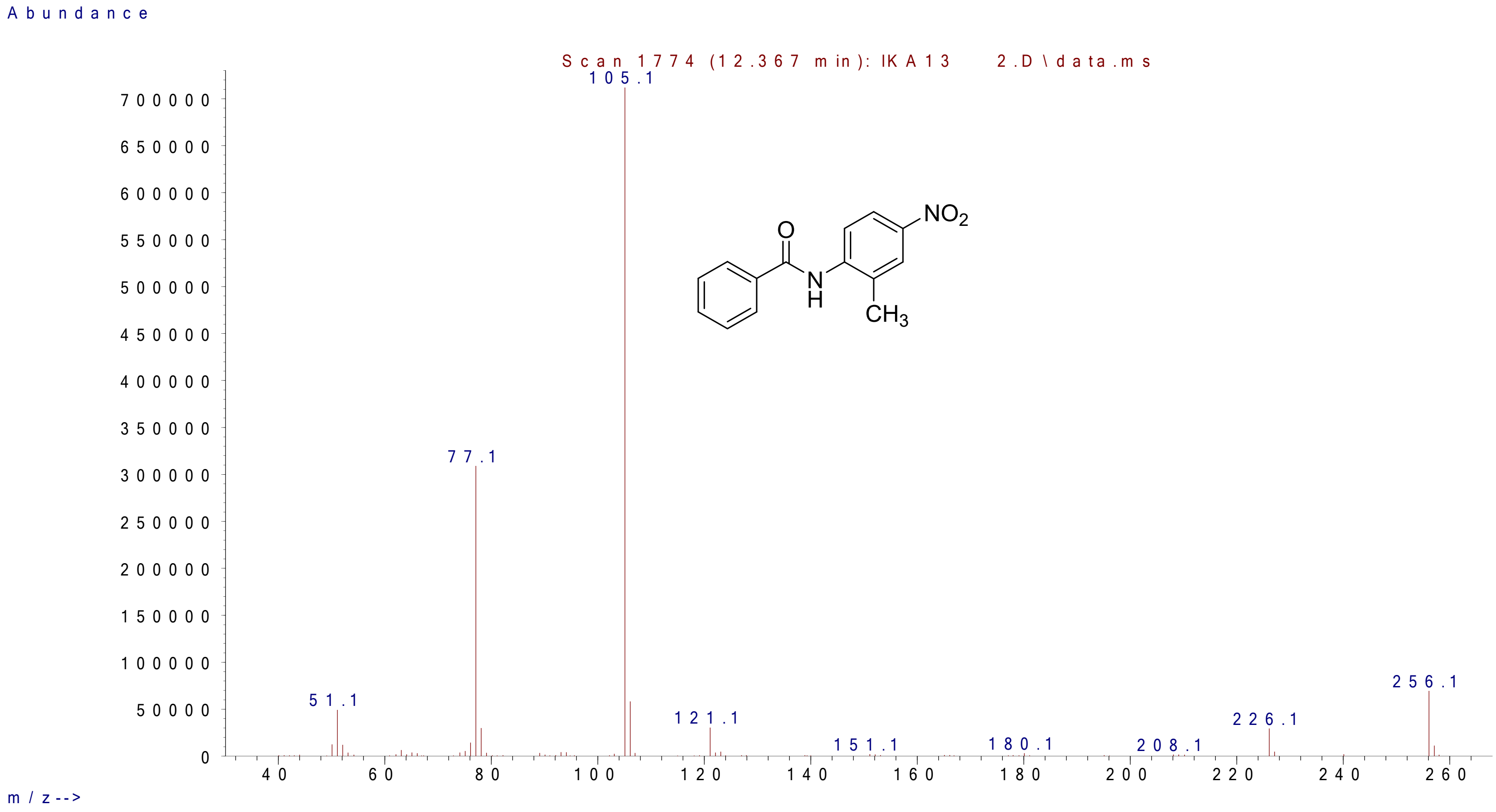
Abundance
Scan 1774 (12.367 min): IKA13 2.D\data.ms
105.1
77.1
51.1
121.1
151.1
180.1
208.1
226.1
256.1
700000
650000
600000
550000
500000
450000
400000
350000
300000
250000
200000
150000
100000
50000
0
40
60
80
100
120
140
160
180
200
220
240
260
m/z-->
NO2
O
N
H
CH3

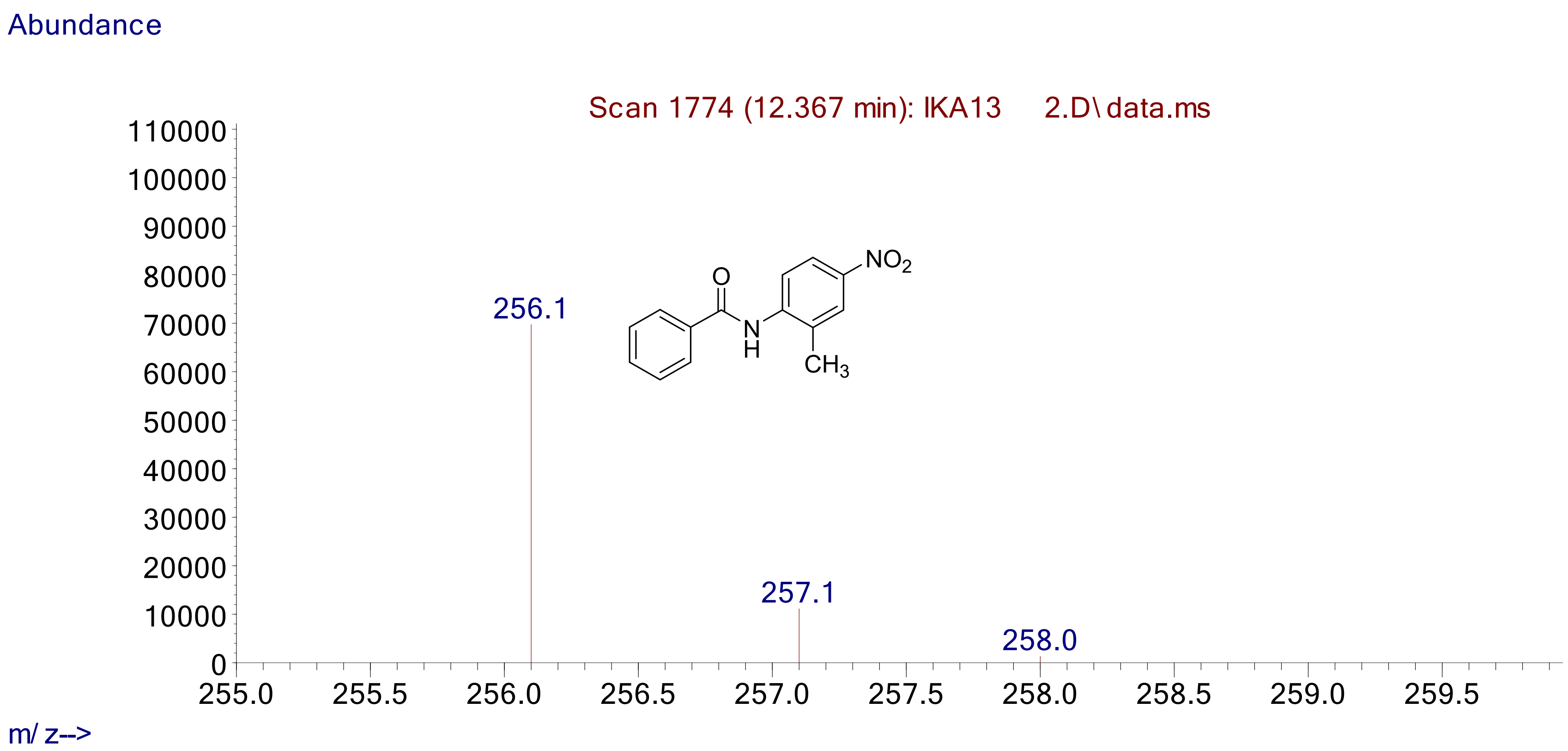

Abundance
Scan 1774 (12.367 min): IKA13 2.D\ data.ms
110000
100000
90000
80000
70000
60000
50000
40000
30000
20000
10000
0
256.1
257.1
258.0
255.0
255.5
256.0
256.5
257.0
257.5
258.0
258.5
259.0
259.5
m/ z-->
O
N
H
NO2
CH3

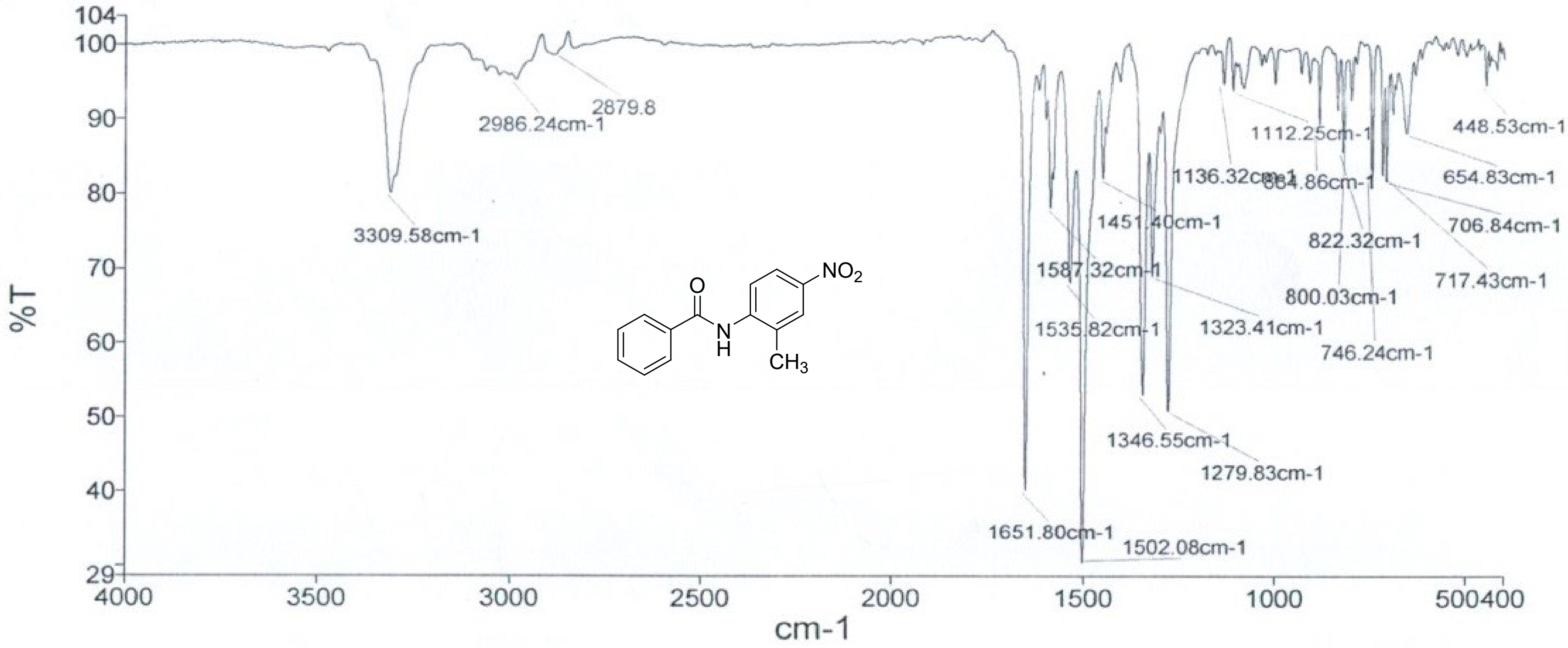

%T
cm-1
104
100
90
80
70
60
50
40
29
4000
3500
3000
2500
2000
1500
1000
500400
3309.58cm-1
2986.24cm-1
2879.8
1651.80cm-1
1587.32cm-1
1535.82cm-1
1502.08cm-1
1451.40cm-1
1346.55cm-1
1323.41cm-1
1279.83cm-1
1136.32cm-1
1112.25cm-1
884.86cm-1
822.32cm-1
800.03cm-1
746.24cm-1
717.43cm-1
706.84cm-1
654.83cm-1
448.53cm-1
O
N
H
NO2
CH3

^{1}H NMR, ^{13}C NMR, mass and IR spectrum of *N*-(naphthalen-1-yl)benzamide (3o)

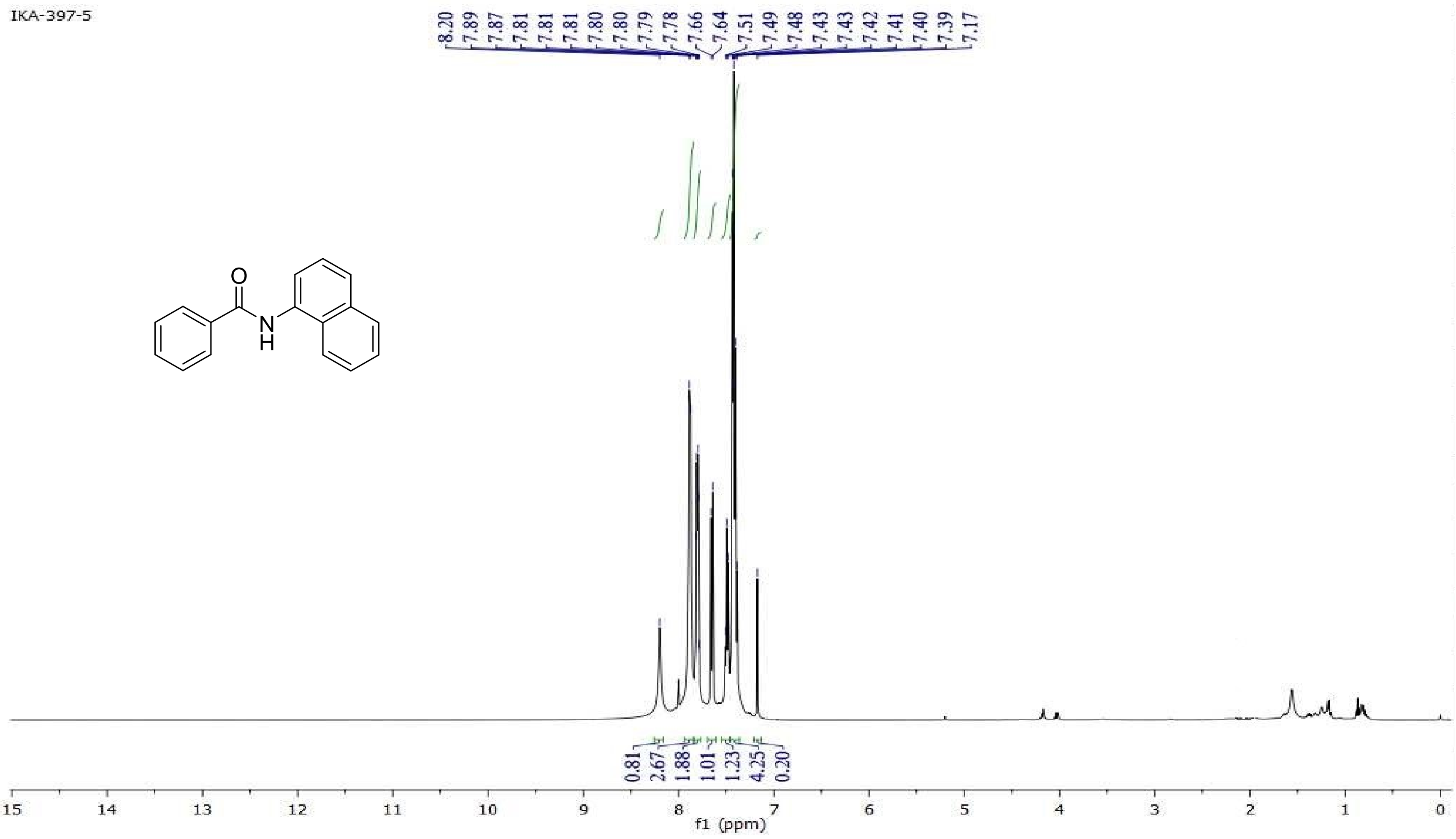

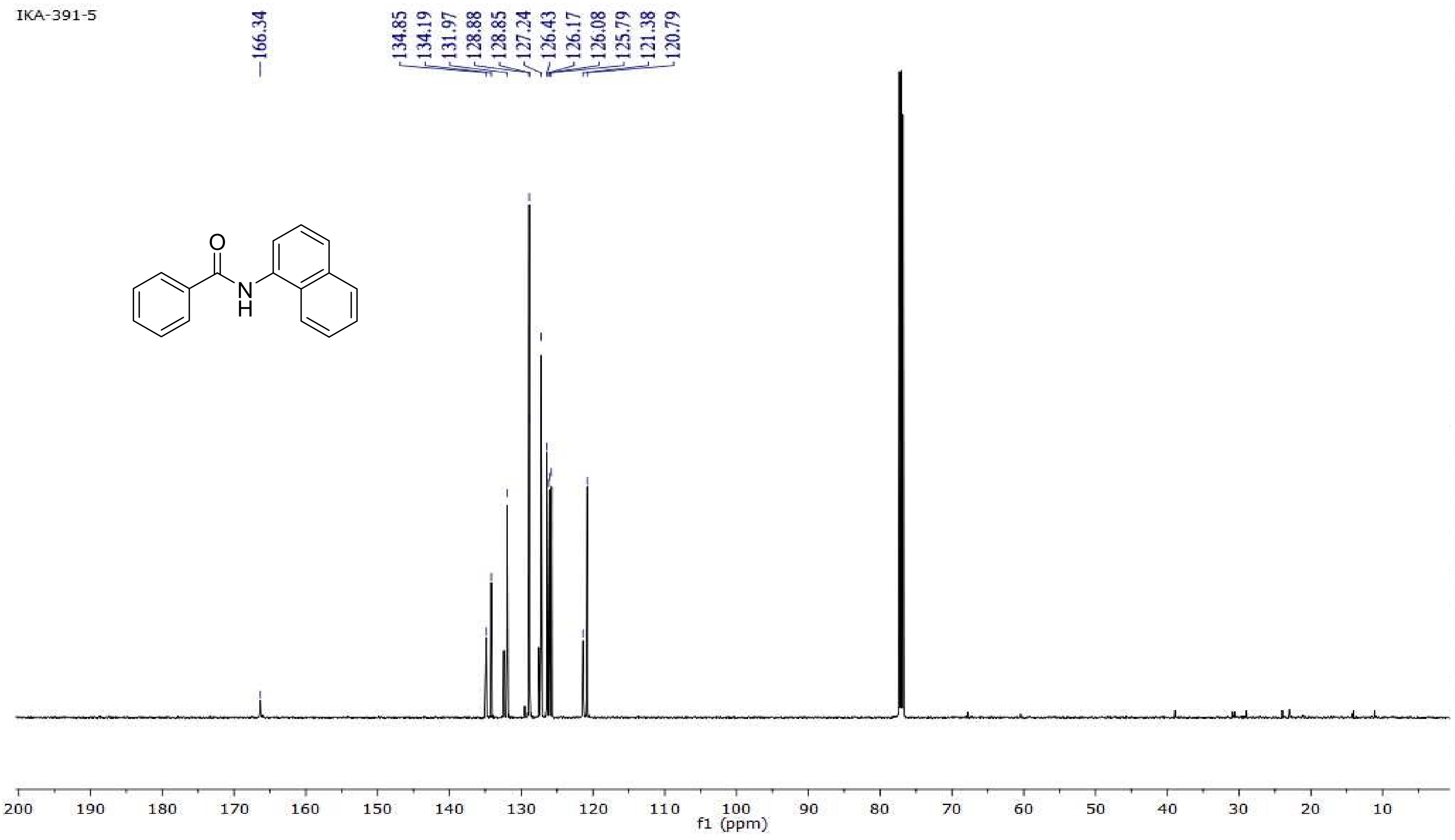

IKA-391-5
166.34
134.85
134.19
131.97
128.88
128.85
127.24
126.43
126.17
126.08
125.79
121.38
120.79
O
N
H
200
190
180
170
160
150
140
130
120
110
100
90
80
70
60
50
40
30
20
10
f1 (ppm)

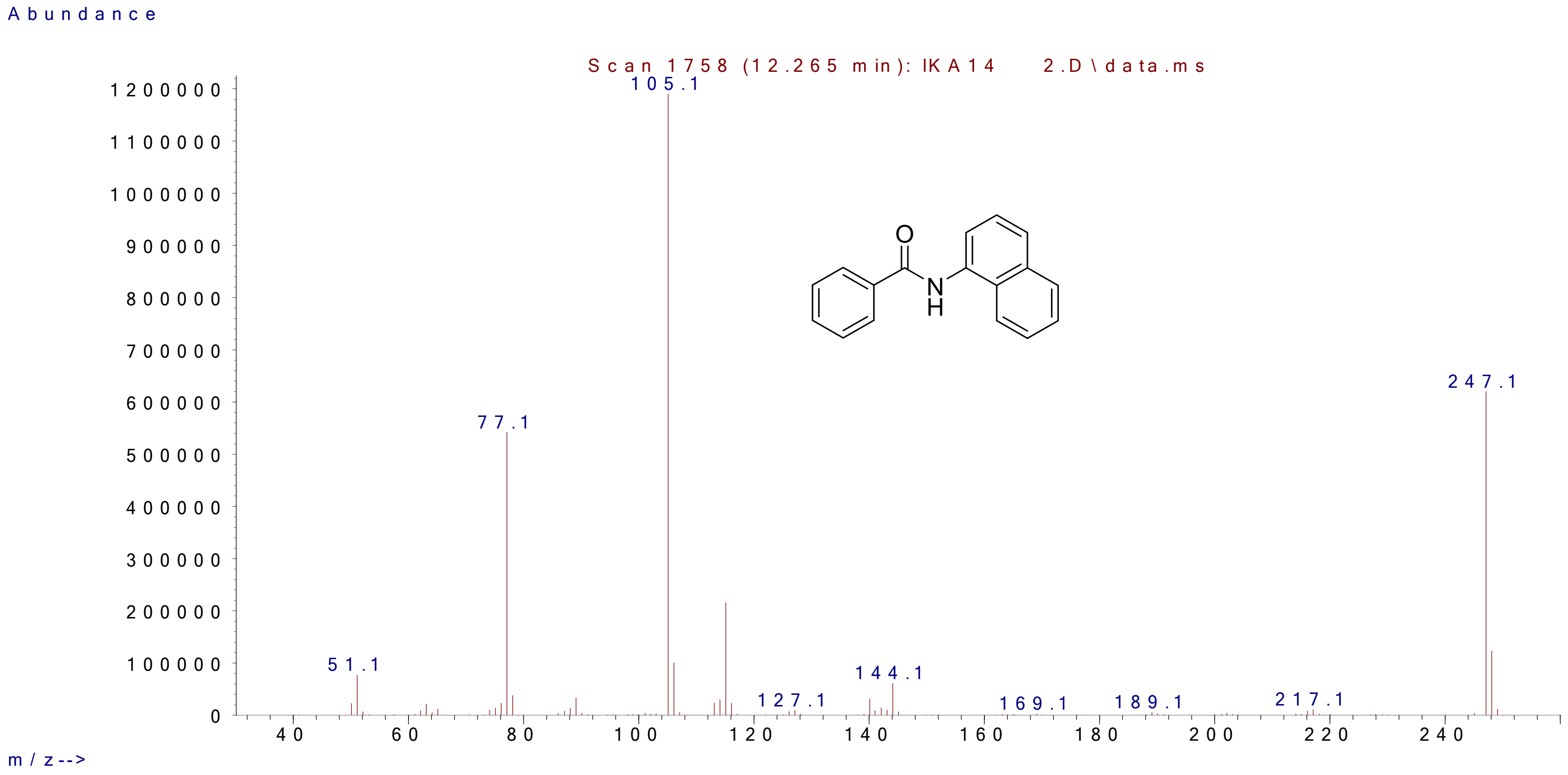

Abundance
Scan 1758 (12.265 min): IKA14 2.D\data.ms
105.1
77.1
247.1
51.1
127.1
144.1
169.1
189.1
217.1
m/z-->

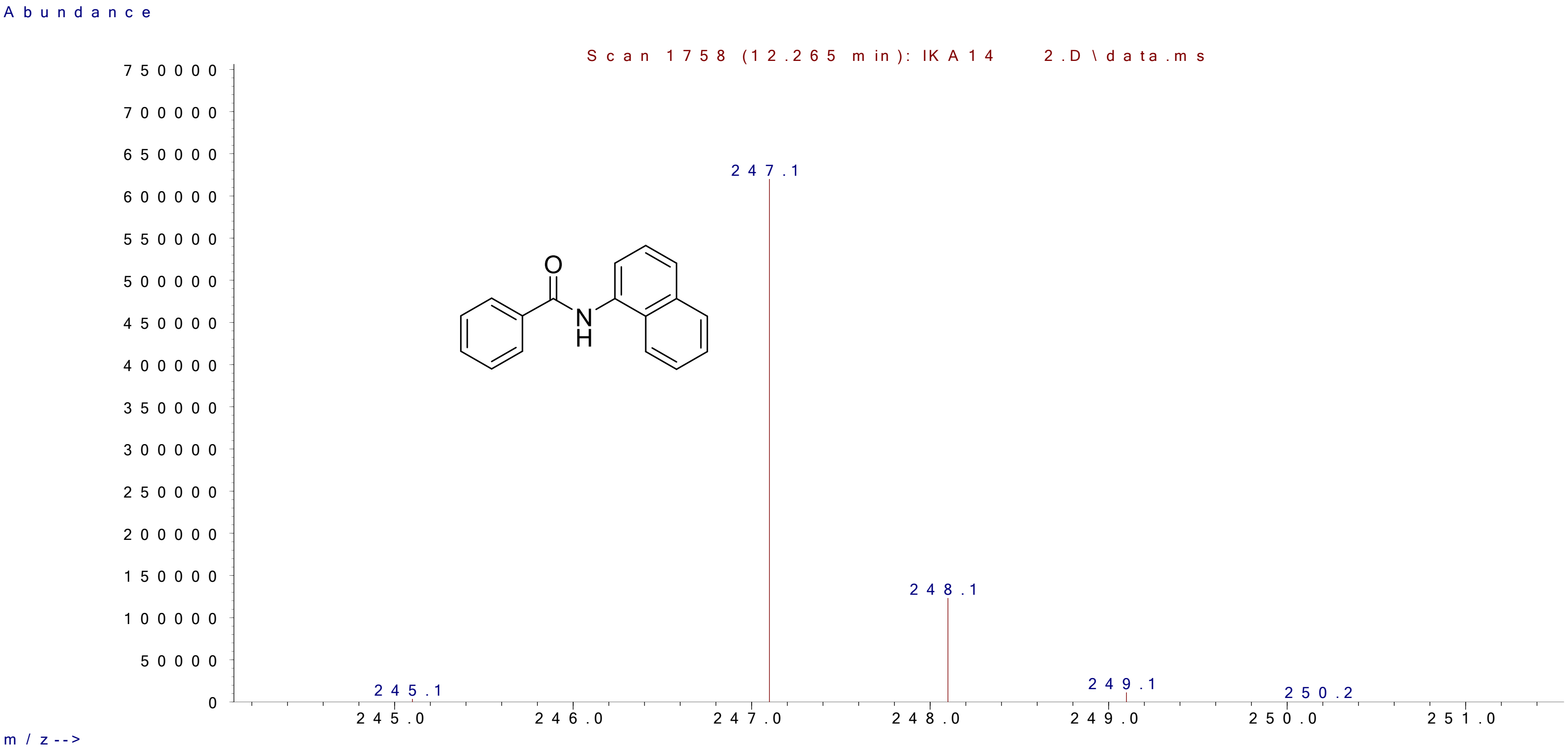
Abundance
Scan 1758 (12.265 min): IKA14 2.D\data.ms
750000
700000
650000
600000
550000
500000
450000
400000
350000
300000
250000
200000
150000
100000
50000
0
247.1
248.1
245.1
249.1
250.2
O
N
H
245.0
246.0
247.0
248.0
249.0
250.0
251.0
m/z-->

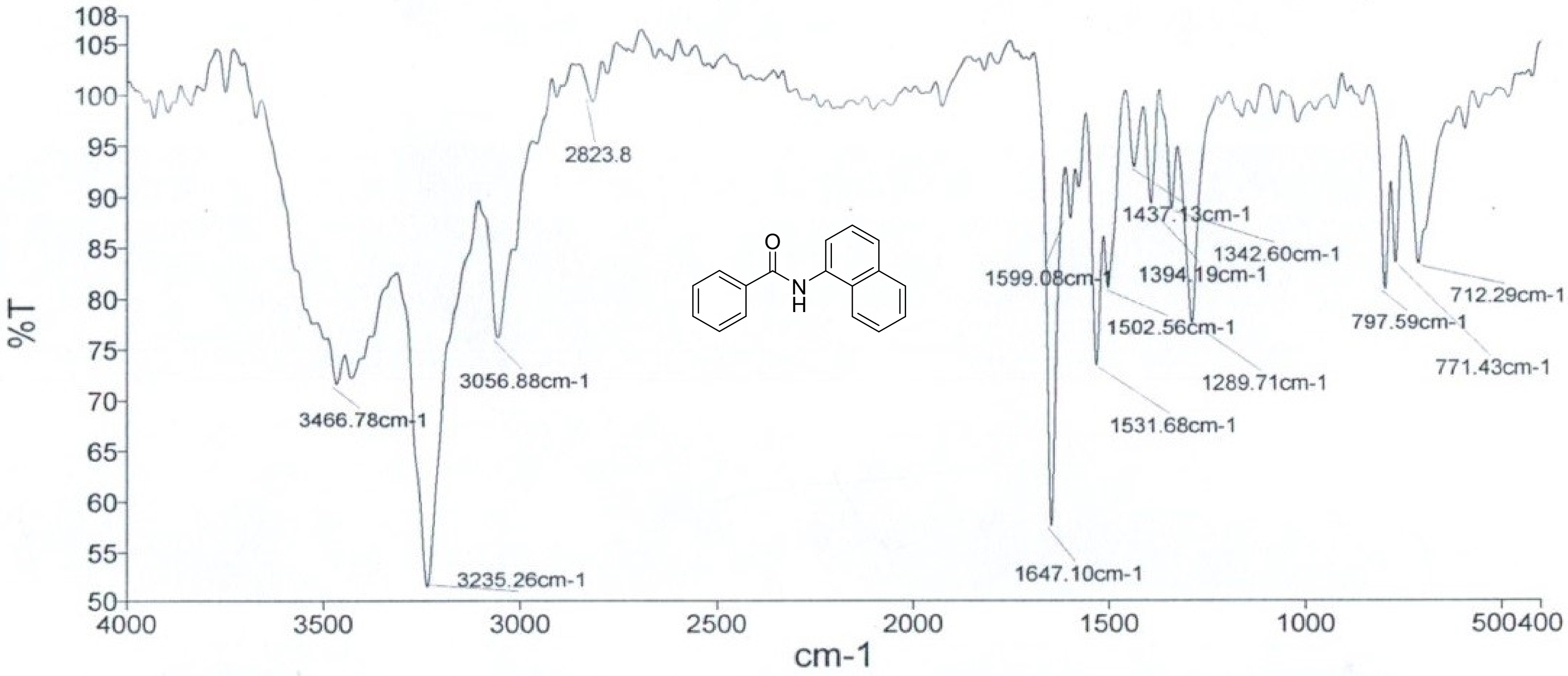

108
105
100
95
90
85
80
75
70
65
60
55
50
%T
4000
3500
3000
2500
2000
1500
1000
500400
cm-1
2823.8
3466.78cm-1
3235.26cm-1
3056.88cm-1
1647.10cm-1
1599.08cm-1
1531.68cm-1
1502.56cm-1
1437.13cm-1
1394.19cm-1
1342.60cm-1
1289.71cm-1
797.59cm-1
771.43cm-1
712.29cm-1
O
N
H
